# Aza-Oxa-Triazole Based Macrocycles with Tunable Properties: Design, Synthesis, and Bioactivity

**DOI:** 10.3390/molecules27113409

**Published:** 2022-05-25

**Authors:** Subba Rao Cheekatla, Liya Thurakkal, Anna Jose, Debashis Barik, Mintu Porel

**Affiliations:** 1 Department of Chemistry, Indian Institute of Technology Palakkad, Palakkad 678557, India; subbarao@chembiol.re.kr (S.R.C.); 201804101@smail.iitpkd.ac.in (L.T.); 201814001@smail.iitpkd.ac.in (A.J.); 202114005@smail.iitpkd.ac.in (D.B.); 2 Environmental Sciences and Sustainable Engineering Center, Indian Institute of Technology Palakkad, Palakkad 678557, India

**Keywords:** macrocycle, tunable properties, aza-oxa, click reaction, tunable ring size, bioactive, serum albumin

## Abstract

A modular platform for the synthesis of tunable aza-oxa-based macrocycles was established. Modulations in the backbone and the side-chain functional groups have been rendered to achieve the tunable property. These aza-oxa-based macrocycles can also differ in the number of heteroatoms in the backbone and the ring size of the macrocycles. For the proof of concept, a library of macrocycles was synthesized with various hanging functional groups, different combinations of heteroatoms, and ring sizes in the range of 17–27 atoms and was characterized by NMR and mass spectrometry. In light of the importance of the copper-catalyzed azide-alkyne cycloaddition (CuAAC) reaction and the significance of triazole groups for various applications, we employed the click-reaction-based macrocyclization. The competence of the synthesized macrocycles in various biomedical applications was proven by studying the interactions with the serum albumin proteins; bovine serum albumin and human serum albumin. It was observed that some candidates, based on their hanging functional groups and specific backbone atoms, could interact well with the protein, thus improving the bioactive properties. On the whole, this work is a proof-of-concept to explore the backbone- and side-chain-tunable macrocycle for different properties and applications.

## 1. Introduction

Macrocycles are a class of molecules with a remarkable impact on bioactive natural products, chemical biology, drug design and development, bioorganic chemistry, medicinal chemistry, host–guest chemistry, and materials chemistry [1,2,3,4,5]. The structural preorganization, conformational stability, and proficiency in molecular recognition of the macrocycles molded them into a competent class for broad applicability. Macrocyclic assembly is deliberated as a challenging task and a critical step in macrocycle synthesis. Several reports are available in the literature on the synthesis of macrocyclic scaffolds via various macrocyclization processes [6,7,8]. These include the metathesis (RCM) reactions [9], transition-metal-catalyzed coupling reaction [10], macrolactonization [11], CuAAC click chemistry [12], macrolactamization [13], thiol-ene photochemical approach [14], S_N_2 and S_N_2Ar reactions [15], palladium-catalyzed cross-coupling reactions [16], Horner−Emmons olefination [17], Pd(0)-mediated Larock indole annulation [18], photoinduced intramolecular radical macrocyclization [19], Ugi reaction [20], and intramolecular Diels–Alder reaction [21]. However, many reported macrocycles do not hold tunable properties. The slot for accommodating different functional groups in the backbone and side chain can modulate their properties significantly for various applications, depending on the need. There are a few reports on the macrocyclic systems where the focus has been given to tunable functional groups and ring size to modulate their utility [22,23]. However, most of them do not have the potential to tune the functional groups in the backbone of the macrocycle.

Aza-oxa-based macrocyclic systems are well known from crown ethers, the first synthetic macrocycles. All oxygen crown ethers and all nitrogen crown ethers showed different properties as receptors for molecular recognition processes [24]. The number of these heteroatoms in the cyclic backbone also impacts their binding properties. Undoubtedly, a modular platform with different types and a variable number of heteroatoms in the macrocyclic system can show diverse effects in the binding properties for various material and biomedical applications. Triazoles, five-membered nitrogen-containing heterocycles, are strategically incorporated into the macrocyclic scaffold by the copper-catalyzed azide-alkyne cycloaddition (CuAAC) reaction to enhance its versatility for biomedical applications. Interestingly, triazoles can form various non-covalent interactions, such as Van der Waals forces, hydrogen bonding, dipole–dipole, and hydrophobic interactions, with various biological targets with a high affinity. In this regard, these motifs are considered to be critical units for synthesizing various biologically active constructs, as they can also enhance water solubility. Macrocycles comprising triazole cores are worthwhile scaffolds in various fields, particularly in materials, pharmaceutical, medicinal, and supramolecular chemistry [25,26,27]. They can act as chemosensors, molecular receptors, peptidomimetics, drug carriers, and enzyme inhibitors and show exciting features, such as self-assembling and anion-binding properties [28,29,30,31,32]. These are derived from azide- and alkyne-based fragments via click fashion (CuAAC) by inter- and intramolecular cyclization. The CuAAC reaction mainly depends on the conformation of a substrate, solvent, and type of linker between azide and alkyne and its concentration.

The property of the macrocycle is solely dependent on the functionalities in the backbone and the pendant group. This report puts forth a novel design and synthesis of aza-oxa-triazole-based macrocycles with tunable properties. Click chemistry was utilized for the hassle-free macrocyclization and the introduction of an essential triazole moiety. Four classes of macrocycles were synthesized by varying the combination of the heteroatoms (oxygen and nitrogen) in the ring. The synthesized macrocycles were characterized using ^1^H and ^13^C nuclear magnetic resonance (NMR), liquid chromatography-mass spectrometry (LC-MS), and high-resolution mass spectrometry (HRMS), Infrared spectroscopy (IR). As a prefatory study towards biological application, physicochemical studies were carried out in silico to study its druggable nature. In addition, the interaction of each macrocycle with model proteins, bovine serum albumin and human serum albumin, was also investigated.

## 2. Results and Discussion

### 2.1. Synthesis and Characterization

**Scheme 1:**
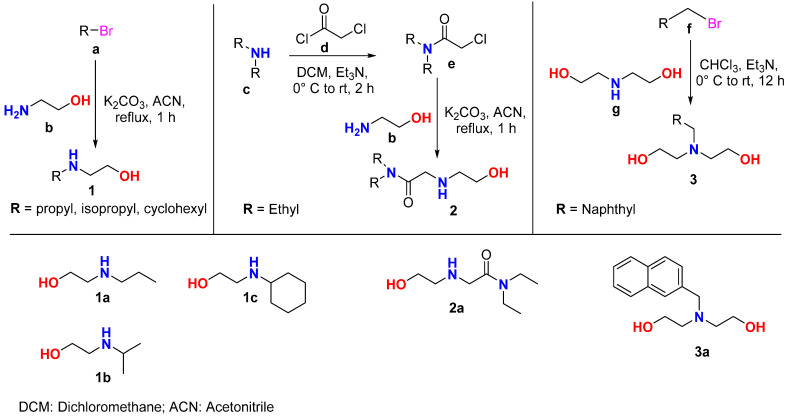
Synthesis of alkyne precursors (amine-hydroxyl derivatives) with N-O combination without amide (**1a**–**1c**), N-O combination with amide functional group (**2a**), and O-N-O combination (**3a**).

The structure of macrocycles has been designed in a way to effectuate functional tunability on the backbone and the pendant group. Subsequently, four classes of macrocycles have been synthesized by differing the N-O combination in the macrocyclic backbone. Intermolecular click reactions executed the macrocyclization by reacting bis-alkynes and bis-azides (macrocyclic precursors). The design of alkynes and azides provided the scope of diverse functional groups in their structure. In this report, we synthesized wide varieties of bis-alkynes, keeping the bis-azide constant for all. The synthetic strategy for bis-alkynes were newly designed, and the strategy to synthesize bis-azide was already reported [33]. Four types of alkyne precursors were synthesized, which were (i) an N-O combination; (ii) an N-N combination; (iii) an O-O combination; and (iv) an O-N-O combination. Precursors for alkynes of the N-N combinations and O-O combinations were commercially available. However, bis-alkynes with N-O and O-N-O combinations were strategically synthesized from amine-hydroxyl precursors. These amine-hydroxyl precursors with diverse functional groups were synthesized as per Scheme 1.

The amine-hydroxyl derivatives, which are the bis-alkyne precursors for N-O combinations, are of two types: (i) without an amide functional group (1, Scheme 1) and (ii) with a pendant amide functional group (2, Scheme 1). The first one was synthesized via the reaction of various alkyl bromides (a, Scheme 1) with ethanolamine (b, Scheme 1**)** in the presence of a base in a reflux condition in acetonitrile (ACN) as the solvent. The formed product (1, Scheme 1) proceeded to the next step after solvent extraction. The amine-hydroxyl derivative with the pendant amide functional group was synthesized by a two-step reaction as per the reported procedure [34]. The first step was the alkyl amine reaction with chloroacetyl chloride (d, Scheme 1) in the presence of triethylamine (TEA) as the base and dichloromethane (DCM) as the solvent to obtain e, Scheme 1. This reaction was followed by the ethanolamine reaction in ACN, and the product (2, Scheme 1) was taken over to the next step. The precursor for the O-N-O combination was synthesized by a reaction of 2-(bromomethyl) naphthalene f, Scheme 1) with diethanolamine (g, Scheme 1) in the presence of TEA. Thus, a naphthyl derivative of diethanolamine (3, Scheme 1) was obtained with a high purity. N, N′-substituted diamines and diols were the other precursors used for bis-alkynes.

**Figure 1:**
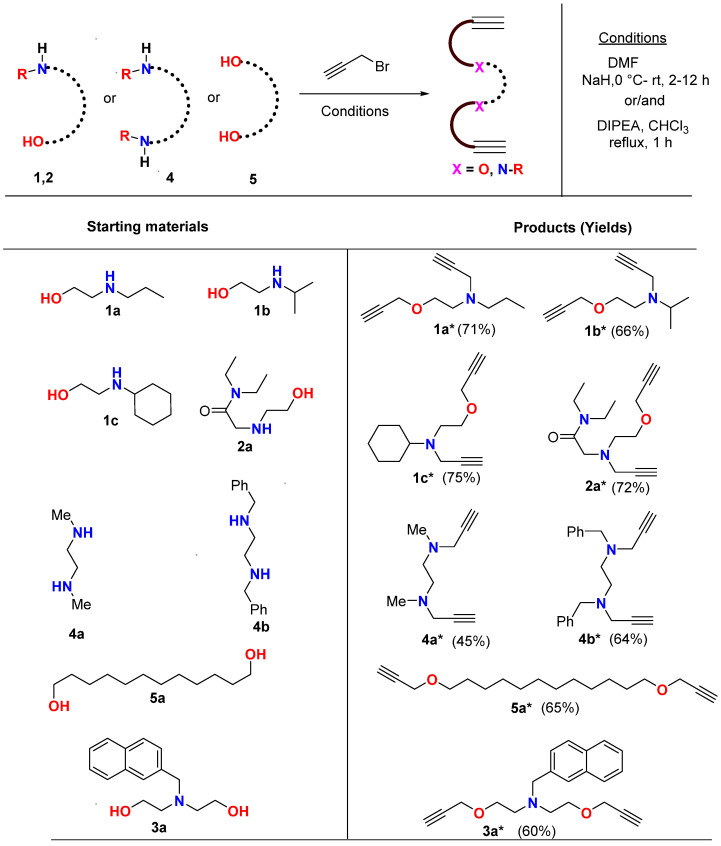
Synthesis of macrocyclic precursors (bis-alkynes).

Bis-alkynes were synthesized by the N-propargylation and/or O-propargylation of diamines, diols, and amine-hydroxyl derivatives, (1a*–1c*, 2a*, 3a*, 4a*, 4b*, and 5a*, Figure 1). N-propargylation was carried out by the reaction of the amine with propargyl bromide in the presence of a strong base, sodium hydride, in DMF. O-propargylation was carried out using NaH as the base for the reaction of diols of various chain lengths with propargyl bromide. This reaction was completed within 2–12 h, while the N-propargylation reaction took 1–2 h for completion in the presence of N,N-Diisopropylethylamine (DIPEA) as the base. The products were purified by column chromatography, and a moderate to good yield was obtained for all the bis-alkyne products (Figure 1). The characterizations of all the bis-alkynes were carried by ^1^H NMR and are given in Figures S7, S9, S11, S13, S15, S17, S19 and S21. Bis-azides are also equally crucial in click reactions; thus, it was synthesized from diethanolamine by reacting with thionyl chloride followed by sodium azide (Scheme S1, Figure S1). The azide used for all the macrocyclization was the same, and it possessed a free secondary amine. The addition of any functional group is conceivable with this amine, and it could also be exploited for post-synthetic modifications based on the requirements.

**Figure 2:**
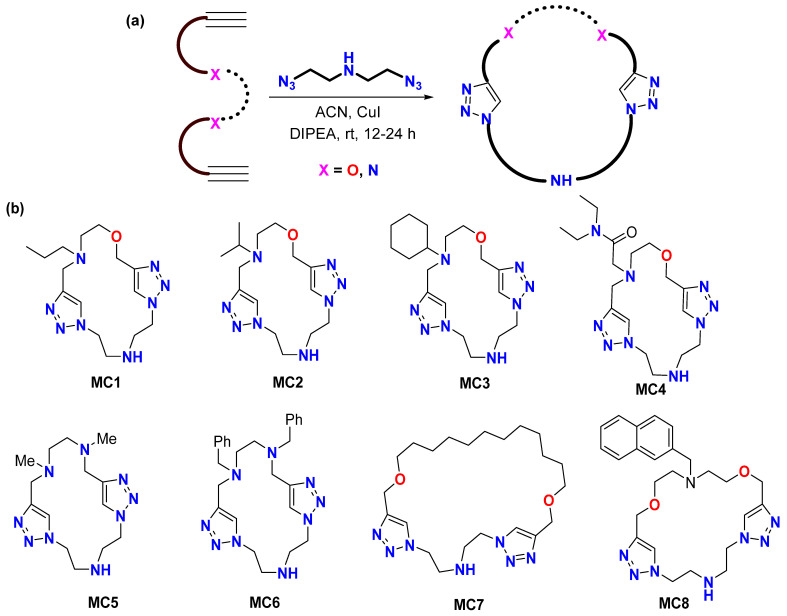

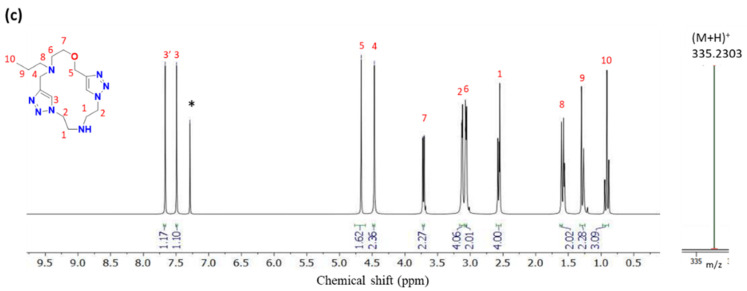
(**a**) The synthetic scheme for macrocyclization reaction; (**b**) Structure of the synthesized macrocycles (ACN: Acetonitrile, CuI: Copper iodide, DIPEA: Diisopropylethylamine, rt: Room temperature); (**c**) Characterization by ^1^H NMR and HRMS. Left, ^1^H NMR of MC1 (“*” is the residual proton signal of solvent CDCl_3_); right, High-resolution mass spectrum with the peak of [M+H]^+^. Calculated for C_15_H_26_N_8_O [M+H]^+^: 335.2308 Da; observed [M+H]^+^: 335.2303 Da.

The most crucial step in a macrocycle synthesis is the macrocyclization reaction, as it determines the efficacy of the total synthesis. Macrocycle formation is seemingly more challenging than the synthesis of linear molecules because of the requirement of overcoming the ring–chain equilibrium. Utmost care must be taken during the macrocycle synthesis to avoid chain formation. The simplest method to comply is to increase the dilution out of various techniques. Consequently, we followed intermolecular CuAAC in a dilute condition by considering the economic aspect as well. The catalytic amount of copper iodide was expended for this bis-alkyne–bis-azide reaction to result in triazole-based aza-oxa macrocycles. The experiment was carried out in ACN with DIPEA as the base and was completed within 12–24 h of stirring. As the proof-of-concept for diverse backbones, pendant functional groups, and ring sizes, a library of macrocycles (MC1–MC8, Figure 2) was synthesized and characterized. Various functional groups, such as propyl, isopropyl, cyclohexyl, methyl, benzyl, and naphthyl were incorporated in the aza-, oxa-, or aza-oxa-based macrocycles. The size of the ring is a crucial factor for a macrocycle when it is used with different applications. This macrocyclic scaffold is substantially tunable to achieve any ring size, according to the requirement. The ring sizes of the macrocycles in Figure 2b range from 17 to 27 atoms. All the macrocycles were well characterized using high-resolution mass spectrometry (HRMS), liquid chromatography-mass spectrometry (LC-MS), infrared (IR) spectroscopy, and nuclear magnetic resonance (NMR) spectroscopy (Figures S23–S54). The LC showed the purity of the compounds, MS showed the [M+H]^+^ peaks, and HRMS confirmed the mass data by [M+H]^+^ peaks up to the high degree of mass resolution. ^1^H-NMR showed the peaks of all significant protons of the macrocycles. A representative ^1^H-NMR and HRMS of MC1 is depicted in Figure 2c.

**Figure 3:**
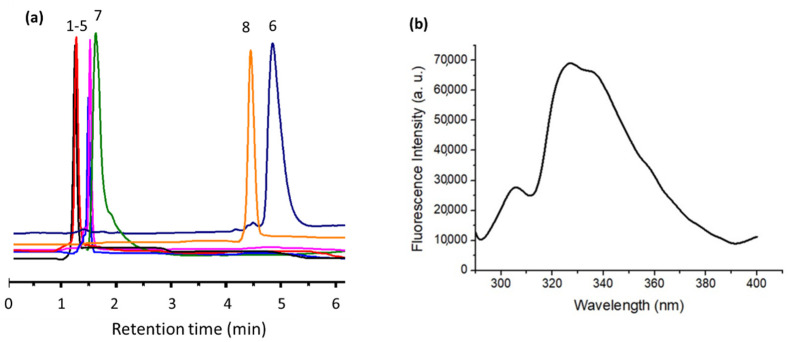
Properties of the macrocycles. (**a**). Reverse-phase high-performance liquid chromatography (RP-HPLC) traces of MC1–8. (Acetonitrile and water with 0.1% formic acid were used as the eluting solvent. The compounds were eluted over 15 min in 5–95 % acetonitrile in water.); (**b**). Fluorescence spectrum of 10 μM MC8 in methanol upon excitation with a wavelength of 278 nm.

The bulk properties of the macrocycles depend on the functional groups on the side chain and backbone and the ring size. The platform for the tunability in the macrocyclic scaffold aided in the synthesis of molecules with a wide range of properties. The hydrophilicity/hydrophobicity of the macrocycles is an important property, primarily for biological applications. The tunable property of the synthesized macrocycles is attributed to the different retention times in the reverse-phase high-performance liquid chromatogram (RP-HPLC). Hydrophilic compounds elute first through the column, thus lowering the retention time, whereas a higher retention time corresponds to the comparatively hydrophobic compounds. From Figure 3a, it could be inferred that the macrocycles MC1–5 and MC7 are hydrophilic, whereas MC8 and MC6 are hydrophobic, as depicted by the higher retention times. The higher hydrophobicity is due to the aromatic rings, two benzyl groups in MC6 and a naphthyl group in MC8. Another property achieved by one of the macrocycles is the addition of fluorophoric group as the side chain. The resultant fluorescence could be exploited for various photophysical applications, including the development of fluorimetric sensors (Figure 3b).

### 2.2. Bioactive Properties of the Macrocycles

**Figure 4:**
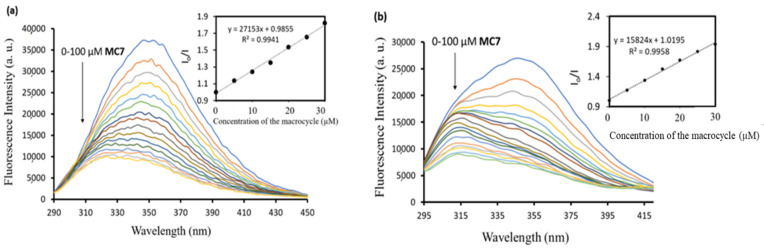
Fluorescence quenching of protein by MC7 at excitation wavelength 280 nm, (**a**) BSA and (**b**) HSA. To 10 µM protein solution in phosphate buffer (pH 7.4), 2–100 µM macrocycle solution was added at 25 °C. Inset: Stern–Volmer Plot; I_o_ and I are the fluorescence intensity at 0 µM and the given concentration of the macrocycle, and the slope is considered as the binding constant.

Macrocycles are intriguing candidates for biological applications due to their enhanced binding with the targets. This property of the macrocycles makes them druggable with many of the target diseases. The preliminary investigation of the druggable nature of a candidate could be predicted by its physicochemical properties. Thus, the druggable behavior by the physicochemical properties of all the synthesized macrocycles was evaluated according to Lipinski’s rule [35,36]. The lipophilicity and physicochemical properties, such as the molecular weight, total polar surface area, number of hydrogen bond acceptors, number of hydrogen bond donors, and number of rotatable bonds, suggested that all the compounds show favorable drug-like properties with no violations of Lipinski’s rule (Table S1). In addition, the druggability of the macrocycles was further studied for protein interaction. Serum albumin is a blood plasma protein in wide occurrence, and it acts as the carrier to transport many drugs through the bloodstream [37]. Hence, we performed a study on the interaction of macrocycles with bovine serum albumin (BSA) and human serum albumin (HSA). Considering the intrinsic fluorescence of BSA due to its tyrosine and tryptophan residues, fluorescence studies were carried out for all the macrocycles. Each macrocycle showed interactions with the proteins to a different extent. Fluorescence quenching was observed for the molecules that interacted well with the protein. The binding constants for the interaction of macrocycles and BSA were calculated using the Stern–Volmer equation (Table 1). It was observed that MC7 showed the highest binding with BSA protein, with a binding constant of 27.1 × 10^3^ M^−1^ (Figure 4a). While MC5 and MC6 showed negligible binding with the protein, MC1, MC2, MC3, and MC4 showed moderate binding (Figures S55–S58). From these results, it could be inferred that oxa macrocycles have the highest binding and aza macrocycles have the least. At the same time, the aza-oxa macrocycles showed a moderate effect. It could also be observed that the amide group as the pendant functional moiety has an added interaction with the protein, as expected from the literature [38], provided by a comparatively higher binding constant for MC4 (Figure S58). The protein interaction study of MC8 would not be conclusive using a fluorescence study, as the peak corresponding to the intrinsic fluorescence of BSA and that of the naphthyl group coincide.

The results were reiterated by studying the interaction of the macrocycles with human serum albumin (HSA), which has a 76% structural similarity with BSA [39]. Comparable results were observed in the fluorescence studies for all the macrocycles. Similar to that of the BSA studies, MC7 displayed the highest interaction and quenching (Figure 4b), and MC6 showed the lowest. Whereas MC1–3,5 resulted in a moderate result, MC4 with the amide group showed a greater extent of interaction, depicted by a higher binding constant. Overall, the change in the functional group in the hanging chain and backbone resulted in a low to moderate to high interaction with the protein, which could be exploited as per the prerequisites of applications. The lower interaction of drug molecules with the protein is unfavorable due to weak binding, and higher binding fails to release the drug as well. Thus, a moderate interaction is favored, and an altered level of serum albumin interaction varies their drug delivery action by changing the pharmacokinetics and pharmacodynamics.

As NMR is a potent technique to scrutinize the kinds of protein–drug molecule interactions, we utilized ^1^H NMR analysis for the same. A shift in the NMR signals of the macrocycle–protein complex when compared to the unbound BSA and macrocycle confirmed the interaction of BSA and the macrocycle (Figure S68). A computational study was also performed to confirm that the change in the backbone, pendant functional groups, and the size of the macrocycle could change the extent of interaction with the proteins. A molecular docking technique using Autodock Vina was used to calculate the lowest binding energy with which the macrocycles bind with the proteins BSA and HSA. As shown in Table S2, MC7 showed highest binding with BSA and HSA with binding energies of −9.2 and −10.4 kcal/mol, respectively, which was expected from the binding constant calculated from the fluorescence study. MC1–MC3, which showed moderate binding in the experimental studies, also showed moderate binding in the computational studies, with binding energy ranging from −7.3 to −8.7 kcal/mol with BSA and HSA. MC5 showed a lower binding interaction with both BSA and has, as expected from the experimental studies. Nevertheless, MC6, which was expected to show lower binding, showed an exceptional behavior with both BSA and HSA in silico by giving low binding energies of −9.3 and −9.1 kcal/mol. The 2D interaction plots given in Figures S69–S82 portrayed the various secondary interactions, such as hydrogen bonding, the Van-der-Waals interaction, the pi-alkyl interaction, etc., made by the macrocycles with the amino acid residues in the proteins.

## 3. Materials and Methods

### 3.1. Synthesis of Bis-Azide

The desired bis-azide was prepared in two steps: chlorination followed by azidation via the reported method [33]. The commercially available amino diol was subjected to thionyl chloride in DCM, stirring for 24 h to deliver the dichloramine, and later, this chloro derivative was subjected to sodium azide in water at 80–90 °C to give the bis-azide as a colorless oil in good yield (70%).

### 3.2. Synthesis of Alkyne Precursors (Amino-Alcohols)

To a stirred solution of substituted halides *(a,* 24 mmol) and chloro-amides *(e*, 24 mmol, 1 equiv.) in ACN (30 mL) at room temperature, the ethanolamine (2.1 g, 2 mL, 1.5 equiv.) and K_2_CO_3_ (5 g, 1.5 equiv.) were added. Later on, the resulting reaction mixture was refluxed for 1 h. The reaction progress was monitored by thin-layer chromatography (TLC), and the crude material was passed through the filter paper and washed with ethyl acetate (2–3 times, 20 mL). The filtrate was evaporated under reduced pressure, followed by a propargylation reaction of crude derivatives (amino-alcohols) without purifications.

### 3.3. Preparation of Chloro-Amides

To a solution of substituted amines (1 mmol) in DCM, Et_3_N was added, and the resulting mixture was stirred for 10 min at room temperature. Later on, chloroacetyl chloride (170 mg, 0.120 mL, 1.5 equiv.) was added dropwise at 0 °C over 10 min. Furthermore, the reaction mixture was brought to room temperature and stirred for 2 h at the same temperature. The completeness of the reaction was based on the TLC analysis. The reaction mixture was diluted with DCM and washed with water, NaHCO_3_, and brine solution. The organic layer (DCM) was dried over anhydrous Na_2_SO_4_, and the product was concentrated under the reduced pressure. The obtained crude chloro-amides were used directly without any purification in further reactions.

### 3.4. Preparation of Mono/di-Alkynes: General Procedure for N-Propargylation

To stirred solutions of amines/aminols **1a, 1b, 1c, 2a, 4a,** and **4b** (500 mg to 1.0 g, 3.49–9.70 mmol) in CHCl_3_ (10–20 mL), DIPEA was added (1.20–3.60 mL, 6.98–22.68 mmol, 2–4 equiv.), followed by propargyl bromide (0.50–1.70 mL, 6.98–22.68 mmol, 2–4 equiv.). Next, the reaction mixture was stirred for 1 h in reflux. The reaction progress was monitored by TLC, and the reaction mixture was quenched with water and extracted with CHCl_3_. The organic layers were washed with brine, dried over anhydrous Na_2_SO_4_, and evaporated under a vacuum. The crude mixture was purified by column chromatography using 60–120-mesh silica gel with 20–40% EtOAc/hexane as a solvent system to afford the di-alkynes.

### 3.5. Preparation of Bis-Alkynes: General Procedure for O-Propargylation

To a suspension of NaH (80–270 mg, 3.30–11.41 mmol, 2–4 equiv.) in DMF (1–5 mL), diols and amino-alcohols **1a, 1b, 1c, 2a, 3a,** and **5a** (300–700 mg, 1.65–3.54 mmol, 1 equiv.) were added at 0 °C. Then, the reaction mixture was stirred for 10 min at room temperature. Later on, propargyl bromide (0.3–0.9 mL, 3.30–11.41 mmol, 2–4 equiv.) was added dropwise and continued the reaction until 2-12 hrs. After the completion of the reaction by TLC analysis, a saturated solution of NH_4_Cl was added to quench the reaction mixture, and the resulting aqueous layer was extracted with EtOAc. The combined organic layers were washed with brine solution, and anhydrous Na_2_SO_4_ was used to make it moisture-free. The solvent was evaporated under reduced pressure and subjected to column chromatography with 60–120-mesh silica gel using 0–40% EtOAc/petroleum ether as an eluent to afford pure di-alkynes.

### 3.6. Synthesis of Triazole-based Macrocycles: General Procedure for Azide-Alkyne Click (CuAAC) Cyclization

To a stirred solution of diazide (180–560 mg, 1.19–3.62 mmol, 1 equiv.) and bis-alkyne (200–800 mg, 1.19–3.62 mmol, 1 equiv.) derivatives in degassed ACN (25–100 mL), CuI (0.12–0.36 mmol, 0.1 equiv.) and DIPEA (3.57–10.86 mmol, 3 equiv.) in degassed ACN (25 mL) were added dropwise over 10 min at room temperature under nitrogen atmosphere. The resulting mixture was stirred at this temperature for 16–24 h. The resulting reaction mixture was extracted with CH_2_Cl_2_/CHCl_3_. The brine solutions were used to wash the organic layers, anhydrous Na_2_SO_4_ was used for drying, and the mixture was subjected to vacuum for concentration. The crude residue was purified through a column chromatography with a 60–120-mesh silica gel by elution with 0–10% MeOH in EtOAc to yield the respective pure macrocycles MC1–8 containing bis-triazoles with good yields.

### 3.7. Protein Interaction Studies by Fluorescence Spectroscopy

All the fluorescence spectra were recorded on a Perkin Elmer (Waltham, MA, USA) FL 6500 at 25 °C. The excitation wavelength of 280 nm and a slit width of 5 nm were used for excitation and emission. Next, 6.6 mg of BSA/HSA was dissolved in 10 mL of pH 7.4 phosphate buffer to make 10 μM BSA/HSA. The stock solutions of macrocycles were of 2 mM concentration dissolved in DMSO. The titrations were carried out in varied concentrations of macrocycles from 2 μM to 100 μM. Three minutes were given as the equilibration time after the addition of the solution. The Stern–Volmer plot was used to calculate the binding constant (slope) by plotting I_o_/I vs. the macrocycle concentration.

### 3.8. LC-MS, HRMS, and NMR

A Shimadzu (Kyoto, Japan) LC-MS-8045 with a Sprite TARGA C18 column (40 × 2.1 mm, 5 µm) was used for the LC-MS analysis. The spectra were monitored at 254 nm and 210 nm with a positive mode for mass detection. Water and acetonitrile with 0.1% formic acid were used as eluting solvents A and B. A gradient of 5%, 60%, 90%, and again 5% of acetonitrile with 0.5 mL/min over the time of 15 min was used for eluting the compounds. HRMS was carried out in a Bruker, Maxis Impact and Waters (Billerica, MA, USA) ACQUITY H-CLASS + UPLC/XevoG2 XS QTOF instrument. A 500 MHz Bruker AV III was used to record the ^1^H NMR spectrum. The data were analyzed by MestReNova (version 8.1.1). ^1^H NMR shifts are reported in units of ppm relative to tetramethyl silane. The data are presented in the order: chemical shift, peak multiplicity (s = singlet, d = doublet, t = triplet, m = multiplet), and proton number.

### 3.9. Molecular Docking Studies

Autodock Vina [40] was used to perform the molecular docking studies with a Perl Script for integration executables. The pdb structure of the proteins BSA (4F5V, Chain A) and HSA (1BM0, Chain A) were retrieved from the protein data bank on 12 May 2022 (http://www.rcsb.org). Autodock tools 4 [41] was used to add hydrogen atoms and to compute Gasteiger charges. The grid used for the docking was of the size (60 × 60 × 60) and centered at (x52.09, y22.81, z66.1) for BSA and size (60 × 60 × 60) and centered at (x29.60, y31.78, z23.48) for HSA. The ligands used were energy-minimized using Argus lab 4.0.1 [42], the docked structures were visualized in Pymol, and the amino acid residue interactions were studied using Discovery studio [43].

## 4. Conclusions

This work provides an extension to the important triazole-based macrocycles by introducing tunability in the properties and functions. This is achieved by various combinations of aza-oxa groups in the backbone of the macrocycles with different pendant functional groups. The CuAAC reaction was employed for the crucial macrocycle synthesis and resulted in products in good yields. The synthesized macrocycles showed favorable physicochemical properties to function as druggable candidates and showed good interactions with the serum albumin as well. It was also validated that the binding constant of the macrocycles with the serum albumin proteins can be modulated via tuning of the functional group in the backbone and side chain. On the whole, this synthetic strategy for tunable aza-oxa-based macrocycles could be widely exploited for obtaining candidates with different properties and functions for diverse applications.

## Figures and Tables

**Table 1:** The binding constant of the macrocycles with the proteins BSA and HSA.

Macrocycle	Binding Constant (×10^3^ M^−1^)	
	BSA	HSA
MC1	4.6	4.2
MC2	4.0	4.4
MC3	4.2	3.5
MC4	9.1	8.4
MC5	1.4	3.2
MC6	0.8	0.93
MC7	27.1	15.8

## Data Availability

Not applicable.

## Supplementary Materials

The following supporting information can be downloaded at: https://www.mdpi.com/article/10.3390/molecules27113409/s1, Figure S1–S22: the characterization of precursors, Figure S23–S54: the characterization of macro-cycles, Figure S55–S67: fluorescence studies of macrocycles with BSA and HSA, Figure S68: NMR of macrocycle with protein, Figure S69–S82: docking results, Table S1: physicochemical studies of the macrocycle and Table S2: Molecular docking of macrocycles with BSA and HAS, Scheme S1: Synthesis of bis-azide diethanolamine (DCM: Dichloromethane), Scheme S2: The synthesis of mono alkyne from amine hydroxyl propyl derivative, Scheme S3: The synthesis of mono alkyne from amine hydroxyl isopropyl derivative, Scheme S4: The synthesis of mono alkyne from amine hydroxyl cyclohexyl derivative, Scheme S5: The synthesis of mono alkyne from amine hydroxyl diethyl amide derivative, Scheme S6: The synthesis of bis-alkyne from N, N’-dimethyl ethylene diamine, Scheme S7: The synthesis of bis-alkyne from N, N’-dibenzyl ethylene diamine, Scheme S8: The synthesis of bis-alkyne from propyl substituted mono alkyne, Scheme S9: The synthesis of bis-alkyne from isopropyl substituted mono alkyne, Scheme S10: The synthesis of bis-alkyne from cyclohexyl substituted mono alkyne, Scheme S11: The synthesis of bis-alkyne from diethyl amide substituted mono alkyne, Scheme S12: The synthesis of bis-alkyne from dodecane 1,12-diol, Scheme S13: The synthesis of bis-alkyne from 2-methyl naphthalene substituted.

## Sample Availability

May be available upon request from corresponding authors.

## References

[1] Yu J., Qi D., Li J. (2020). Design, synthesis and applications of responsive macrocycles. Commun. Chem..

[2] Marti-Centelles V., Pandey M.D., Burguete M.I., Luis S.V. (2015). Macrocyclization Reactions: The Importance of Conformational, Configurational, and Template-Induced Preorganization. Chem. Rev..

[3] Mallinson J., Collins I. (2012). Macrocycles in new drug discovery. Futur. Med. Chem..

[4] Yu X., Sun D. (2013). Macrocyclic Drugs and Synthetic Methodologies toward Macrocycles. Molecules.

[5] Frank A.T., Farina N.S., Sawwan N., Wauchope O.R., Qi M., Brzostowska E.M., Chan W., Grasso F.W., Haberfield P., Greer A. (2007). Natural macrocyclic molecules have a possible limited structural diversity. Mol. Divers..

[6] Mortensen K.T., Osberger T.J., King T.A., Sore H.F., Spring D.R. (2019). Strategies for the Diversity-Oriented Synthesis of Macro-cycles. Chem. Rev..

[7] Chen C.-F., Han Y. (2018). Triptycene-Derived Macrocyclic Arenes: From Calixarenes to Helicarenes. Accounts Chem. Res..

[8] Park C., Burgess K. (2001). Facile Macrocyclizations to β-Turn Mimics with Diverse Structural, Physical, and Conformational Prop-erties. J. Comb. Chem..

[9] Gradillas A., Pérez-Castells J. (2006). Macrocyclization by Ring-closing Metathesis in the Total Synthesis of Natural Products: Reaction Conditions and Limitations. Angew. Chem. Int. Ed..

[10] Rivera D.G., Ojeda-Carralero G.M., Reguera L., van der Eycken E.V. (2020). Peptide Macrocyclization by Transition Metal Catalysis. Chem. Soc. Rev..

[11] Parenty A., Moreau X., Campagne J.-M. (2006). Macrolactonizations in the Total Synthesis of Natural Products. Chem. Rev..

[12] Pasini D. (2013). The Click Reaction as an Efficient Tool for the Construction of Macrocyclic Structures. Molecules.

[13] White C.J., Yudin A.K. (2011). Contemporary strategies for peptide macrocyclization. Nat. Chem..

[14] Aimetti A.A., Shoemaker R.K., Lin C.-C., Anseth K.S. (2010). On-resin peptide macrocyclization using thiol–ene click chemistry. Chem. Commun..

[15] Fitzgerald M.E., Mulrooney C.A., Duvall J.R., Wei J., Suh B.-C., Akella L.B., Vrcic A., Marcaurelle L.A. (2012). Build/Couple/Pair Strategy for the Synthesis of Stereochemically Diverse Macrolactams via Head-to-Tail Cyclization. ACS Comb. Sci..

[16] Nicolaou K.C., Bulger P.G., Sarlah D. (2005). Palladium-Catalyzed Cross-Coupling Reactions in Total Synthesis. Angew. Chem. Int. Ed..

[17] Larsen B.J., Sun Z., Nagorny P. (2013). Synthesis of Eukaryotic Translation Elongation Inhibitor Lactimidomycin via Zn(II)-Mediated Horner–Wadsworth–Emmons Macrocyclization. Org. Lett..

[18] Breazzano S.P., Poudel Y.B., Boger D.L. (2013). A Pd(0)-Mediated Indole (Macro)cyclization Reaction. J. Am. Chem. Soc..

[19] Nishikawa K., Yoshimi Y., Maeda K., Morita T., Takahashi I., Itou T., Inagaki S., Hatanaka M. (2012). Radical Photocyclization Route for Macrocyclic Lactone Ring Expansion and Conversion to Macrocyclic Lactams and Ketones. J. Org. Chem..

[20] Abdelraheem E.M.M., Khaksar S., Kurpiewska K., Kalinowska-Tłuścik J., Shaabani S., Dömling A. (2018). Two-Step Macrocycle Synthesis by Classical Ugi Reaction. J. Org. Chem..

[21] Zapf C.W., Harrison B.A., Drahl C., Sorensen E.J. (2005). A Diels-Alder Macrocyclization Enables an Efficient Asymmetric Synthesis of the Antibacterial Natural Product Abyssomicin C. Angew. Chem..

[22] Thurakkal L., Nanjan P., Porel M. (2022). Design, Synthesis and Bioactive Properties of a Class of Macrocycles with Tunable Func-tional Groups and Ring Size. Sci. Rep..

[23] Madhavachary R., Abdelraheem E.M.M., Rossetti A., Twarda-Clapa A., Musielak B., Kurpiewska K., Kalinowska-Tłuścik J., Holak T.A., Dömling A. (2017). Two-Step Synthesis of Complex Artificial Macrocyclic Compounds. Angew. Chem. Int. Ed..

[24] Krakowiak K.E., Bradshaw J.S., Zamecka-Krakowiak D.J. (1989). Synthesis of aza-crown ethers. Chem. Rev..

[25] Hänni K.D., Leigh D.A. (2010). The Application of CuAAC ‘Click’Chemistry to Catenane and Rotaxane Synthesis. Chem. Soc. Rev..

[26] Tron G.C., Pirali T., Billington R., Canonico P.L., Sorba G., Genazzani A. (2007). Click chemistry reactions in medicinal chemistry: Applications of the 1,3-dipolar cycloaddition between azides and alkynes. Med. Res. Rev..

[27] Agrahari A.K., Bose P., Jaiswal M.K., Rajkhowa S., Singh A.S., Hotha S., Mishra N., Tiwari V.K. (2021). Cu(I)-Catalyzed Click Chemistry in Glycoscience and Their Diverse Applications. Chem. Rev..

[28] García F., Torres M.R., Matesanz E., Sánchez L. (2011). Open aryl triazole receptors: Planar sheets, spheres and anion binding. Chem. Commun..

[29] Wang Y., Bie F., Jiang H. (2010). Controlling Binding Affinities for Anions by a Photoswitchable Foldamer. Org. Lett..

[30] Kumar A., Pandey P.S. (2008). Anion Recognition by 1, 2, 3-Triazolium Receptors: Application of Click Chemistry in Anion Recog-nition. Org. Lett..

[31] Lau Y.H., Rutledge P.J., Watkinson M., Todd M.H. (2011). Chemical sensors that incorporate click-derived triazoles. Chem. Soc. Rev..

[32] Campo V.L., Carvalho I., Da Silva C.H.T.P., Schenkman S., Hill L., Nepogodiev S.A., Field R.A. (2010). Cyclooligomerisation of azido-alkyne-functionalised sugars: Synthesis of 1,6-linked cyclic pseudo-galactooligosaccharides and assessment of their sialylation by Trypanosoma cruzi trans-sialidase. Chem. Sci..

[33] Romański J., Jaworski P. (2016). Synthesis of the novel crown and lariat ethers with integrated 1,2,3-triazole ring. Phosphorus Sulfur Silicon Relat. Elem..

[34] Nanjan P., Jose A., Thurakkal L., Porel M. (2020). Sequence-Defined Dithiocarbamate Oligomers via a Scalable, Support-Free, It-erative Strategy. Macromolecules.

[35] Lipinski C.A., Lombardo F., Dominy B.W., Feeney P.J. (1997). Experimental and computational approaches to estimate solubility and permeability in drug discovery and development settings. Adv. Drug Deliv. Rev..

[36] Daina A., Michielin O., Zoete V. (2017). SwissADME: A free web tool to evaluate pharmacokinetics, drug-likeness and medicinal chemistry friendliness of small molecules. Sci. Rep..

[37] Peters T. (1970). Serum Albumin. Adv. Clin. Chem..

[38] Kumaran R., Ramamurthy P. (2014). Photophysical studies on the interaction of amides with Bovine Serum Albumin (BSA) in aqueous solution: Fluorescence quenching and protein unfolding. J. Lumin..

[39] Steinhardt J., Krijn J., Leidy J.G. (1971). Differences between bovine and human serum albumins. Binding isotherms, optical rotatory dispersion, viscosity, hydrogen ion titration, and fluorescence effects. Biochemistry.

[40] Trott O., Olson A.J. (2010). AutoDock Vina: Improving the speed and accuracy of docking with a new scoring function, efficient optimization, and multithreading. J. Comput. Chem..

[41] Morris G.M., Huey R., Lindstrom W., Sanner M.F., Belew R.K., Goodsell D.S., Olson A.J. (2009). AutoDock4 and AutoDockTools4: Automated docking with selective receptor flexibility. J. Comput. Chem..

[42] Ranjith D. (2019). Molecular Docking Studies of Aloe Vera for Their Potential Antibacterial Activity Using Argus Lab 4.0. 1. Pharma Innov. J..

[43] Biovia D.S. (2019). Discovery Visualizer Studio.

