# Supplementary material for: Aza-Oxa-Triazole Based Macrocycles with Tunable Properties: Design, Synthesis, and Bioactivity
Source: Molecules. 2022 May 25;27(11):3409. doi: 10.3390/molecules27113409 (PMC9182012; doi:10.3390/molecules27113409)
Supplement: Supplementary file 1 [file molecules-27-03409-s001.zip › molecules-1719184-supplementary.pdf]

Supporting Information

Aza-oxa-triazole based Macrocycles with Tunable Properties: Design, Synthesis and Bioactivity

Subba Rao Cheekatla[1,#], Liya Thurakkal[1,#], Anna Jose[1], Debashis Barik[1] and Mintu Porel[1,2,*]

[1]Department of Chemistry

[2]Environmental Sciences and Sustainable Engineering Center

Indian Institute of Technology Palakkad
Kerala 678557, India

[#]Both authors contributed equally

*mintu@iitpkd.ac.in

Contents

1. Materials and Methods S1
2. Synthesis of bis-azide S2
3. Synthesis of alkyne precursors (amino-alcohols) S3
4. Preparation of chloro-amides S3
5. Preparation of mono/di-alkynes: General procedure for N-propargylation S3
6. Preparation of bis-alkynes: General procedure for O-propargylation S10
7. Synthesis of triazole based macrocycles: General procedure for azide-alkyne click S18
8. Bioactive properties of the macrocycles S40

1. Materials and Methods

All the chemicals were purchased from Sigma Aldrich, Alfa Aeser, Spectrochem, Merck and TCI and used without further purification. LC-MS experiments were carried out on a Shimadzu LC-MS-8045 with a Sprite TARGA C18 column (40 × 2.1 mm, 5 µm) monitoring at 210 nm and 254 nm with positive mode for mass detection. Solvents for LC-MS were water with 0.1% formic acid (solvent A) and acetonitrile with 0.1% formic acid (solvent B). Compounds were eluted at a flow rate of 0.5 mL/min with a gradient of 5%, 60%, 90% and again 5% of acetonitrile over the time of 15 minutes. The purification by HPLC is performed on Shimadzu HPLC-20AP instrument by using the same solvent system as that of LC-MS. Compounds were eluted at a flow rate of 19 mL/min with a gradient of 20%, 60%, 75%, 90% and 20% of acetonitrile over 26 minutes. ^{1}H NMR spectra were recorded on Bruker AV III 500 MHz. The data were analyzed by MestReNova (version 8.1.1) (https://mestrelab.com/software/mnova/nmr/). ^{1}H NMR shifts are reported in units of ppm relative to tetramethyl silane. The data are presented in the order: chemical shift, peak

multiplicity (s=singlet, d=doublet, t=triplet, m=multiplet) and proton number. HRMS was measured in Bruker, Maxis Impact and Waters ACQUITY H-CLASS + UPLC/XevoG2 XS QTOF instrument. Fluorescence was recorded on Perkin Elmer FL 6500. All fluorescence spectra are recorded at 25 °C with an excitation wavelength of 278 nm and slit width of 5 nm for excitation and emission. The fluorescence spectra were plotted in OriginPro 8.5.1 (https://www.originlab.com/).

2. Synthesis of bis-azide

The desired bis-azide was prepared in two steps via chlorination followed by azidation via reported method [39]. The commercially available amino diol was subjected to thionylchoride in DCM stirring for 24 h to deliver the dichloroamine and later this chloro derivative was subjected to sodium azide in water at 80-90 °C gave the bis-azide as a colourless oil in good yield.

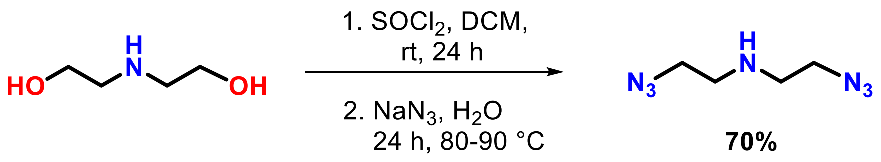

Scheme S1. Synthesis of bis-azide diethanolamine (DCM: Dichloromethane).

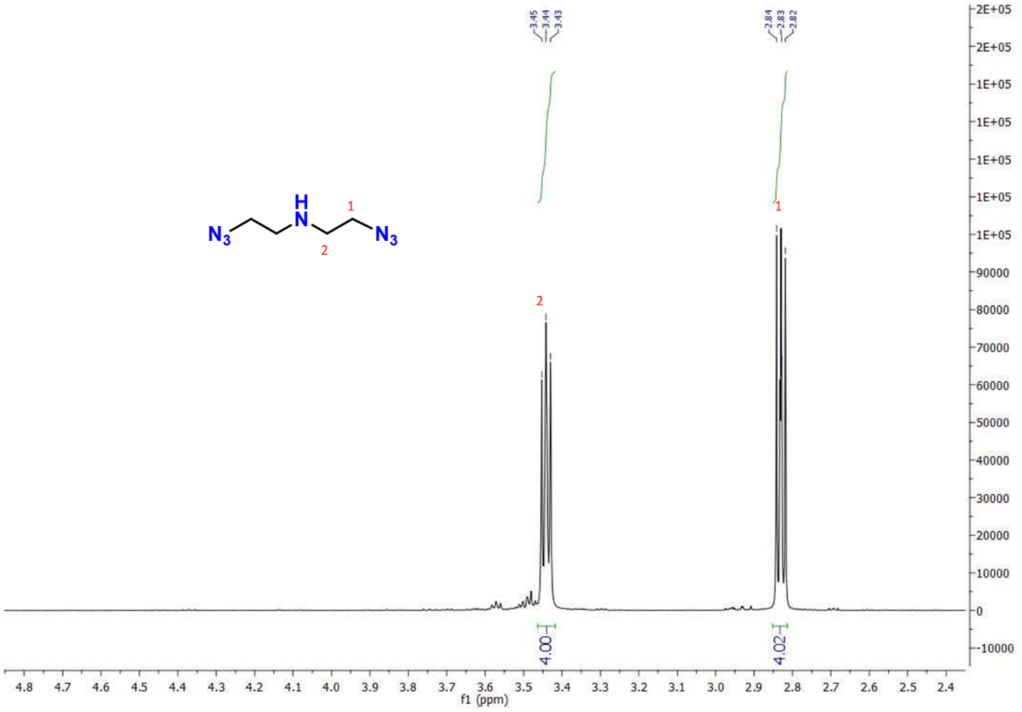

Figure S1. ^{1}H-NMR (500 MHz) of **3a*** in $CDCl_3$: δ (ppm) 2.83 (t, 3H), 3.44 (t, 3H).

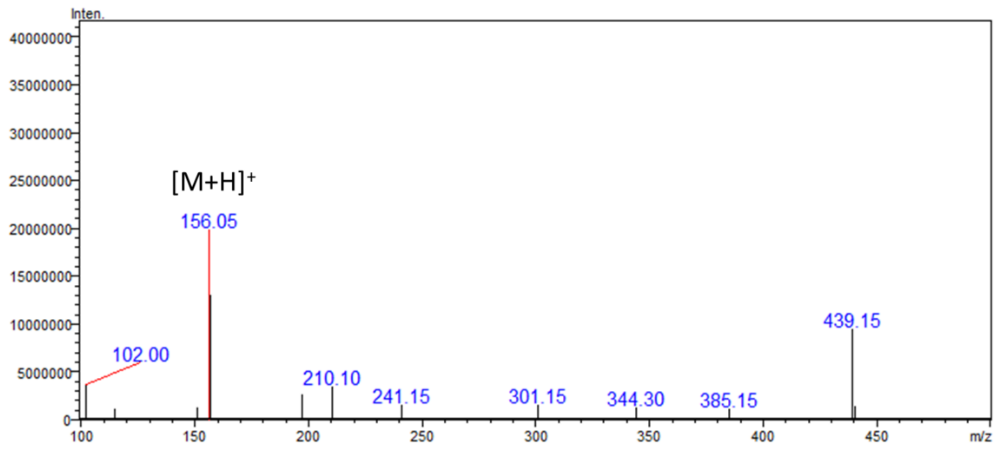

Figure S2. MS of bis-azide. MS calculated $[M+H]^+$: 156.09 Da, observed $[M+H]^+$: 156.05 Da.

3. Synthesis of alkyne precursors (amino-alcohols 1a, 1b and 1c)

To a stirred solution of substituted amines (a) and chloro-amides (d), (24 mmol, 1equiv.) in ACN (30 mL) at room temperature, ethanolamine (2.1 g, 2 mL, 1.5 equiv.) and K_2CO_3 (5 g, 1.5 equiv.) were added. Later on, the resulting reaction mixture was refluxed for 1 h. The reaction progress was monitored by TLC, the crude material was passed through filter paper and washed with ethyl acetate (2-3 times, 20 mL). The filtrate was evaporated under reduced pressure and then crude derivatives (amino-alcohols) were proceeded to propargylation reaction without purifications.

4. Preparation of chloro-amides

A solution of substituted amines (1 mmol) in DCM was added to Et_3N and the resulting mixture was stirred for 10 min at room temperature. Later on, chloroacetyl chloride (170 mg, 0.120 mL, 1.5 equiv.) was added dropwise at 0 °C over 10 min. Further, the reaction mixture was brought to rt and stirred for 2 h at the same temperature. The completeness of the reaction was monitored by TLC analysis, diluted the reaction mixture with DCM and washed with the water, $NaHCO_3$, and brine solution. The organic layer (DCM) was dried over anhydrous Na_2SO_4 and the product was concentrated under the reduced pressure. The obtained crude chloro-amides were used directly without any purification in further reactions.

5. Preparation of mono/di-alkynes (1a#, 1b#, 1c#, 2a#, 4a* and 4b*): General procedure for N-propargylation

To a stirred solution of amines/aminols **1a, 1b, 1c, 2a, 4a** and **4b** (500 mg to 1.0 g, 3.49-9.70 mmol) in $CHCl_3$ (10-20 mL), DIPEA (1.20-3.60 mL, 6.98-22.68 mmol, 2-4 equiv.) and

propargyl bromide (0.50-1.70 mL, 6.98-22.68 mmol, 2-4 equiv.) were added. Next, the reaction mixture was placed to stir at reflux for 1 h. The progress of the reaction was monitored by TLC, the crude reaction mixture was quenched and extracted with $CHCl_3$. The organic layers were washed with brine, dried over anhydrous Na_2SO_4 and evaporated under vacuo. The crude mixture was purified by column chromatography using 60-120 mesh silica gel with 20-40% EtOAc/hexane as solvent system to afford the di-alkynes.

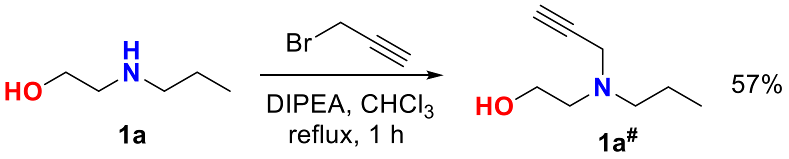

Scheme S2. The synthesis of mono alkyne from amine hydroxyl propyl derivative.

Based on above general procedure,

2-(propylamino)ethan-1-ol (**1a**) (500 mg, 4.84 mmol), DIPEA (1.7 mL, 9.68 mmol, 2 equiv.), propargyl bromide (0.8 mL, 9.68 mmol, 2 equiv.) afford mono-alkyne (**1a**#); colourless viscous liquid; yield: 390 mg (57%).

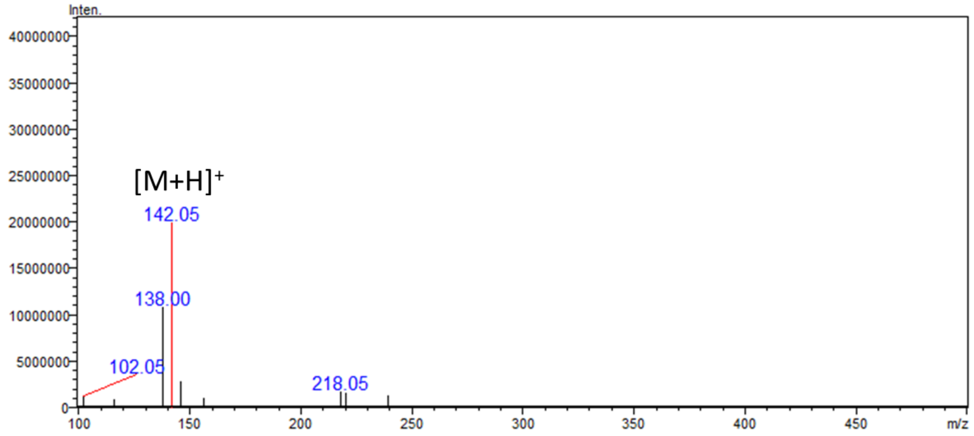

Figure S3. MS of **1a**#. MS calculated $[M+H]^+$: 142.11 Da, observed $[M+H]^+$: 142.05 Da.

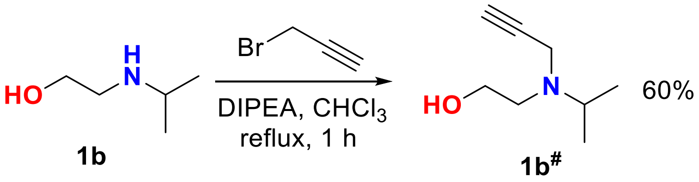

Scheme S3. The synthesis of mono alkyne from amine hydroxyl isopropyl derivative.

Based on above general procedure,

2-(isopropylamino)ethan-1-ol **1b** (1.0 g, 9.69 mmol), DIPEA (3.4 mL, 19.38 mmol, 2 equiv.), propargyl bromide (1.5 mL, 19.38 mmol, 2 equiv.) afford mono-alkyne **1b**#; colourless viscous liquid; yield: 830 mg (60%).

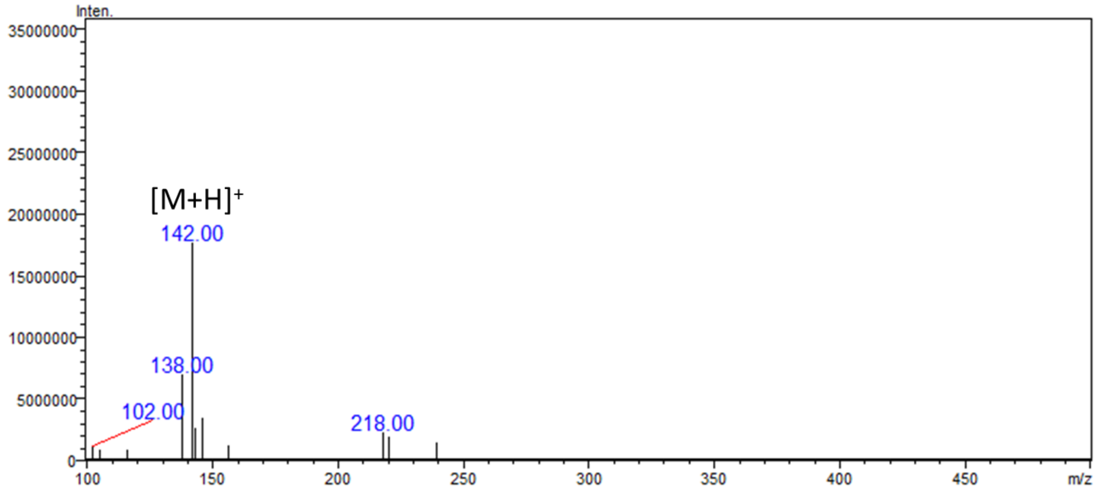

Figure S4. MS of **1b**#. MS calculated $[M+H]^+$: 142.11 Da, observed $[M+H]^+$: 142.00 Da.

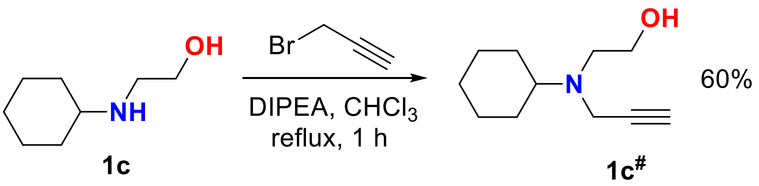

Scheme S4. The synthesis of mono alkyne from amine hydroxyl cyclohexyl derivative.

Based on above general procedure,

2-(cyclohexylamino)ethan-1-ol **1c** (500 mg, 3.49 mmol), DIPEA (1.2 mL, 6.98 mmol, 2 equiv.), propargyl bromide (0.5 mL, 6.98 mmol, 2 equiv.) afford mono-alkyne **1c**#. colourless viscous liquid; yield: 380 mg (60%).

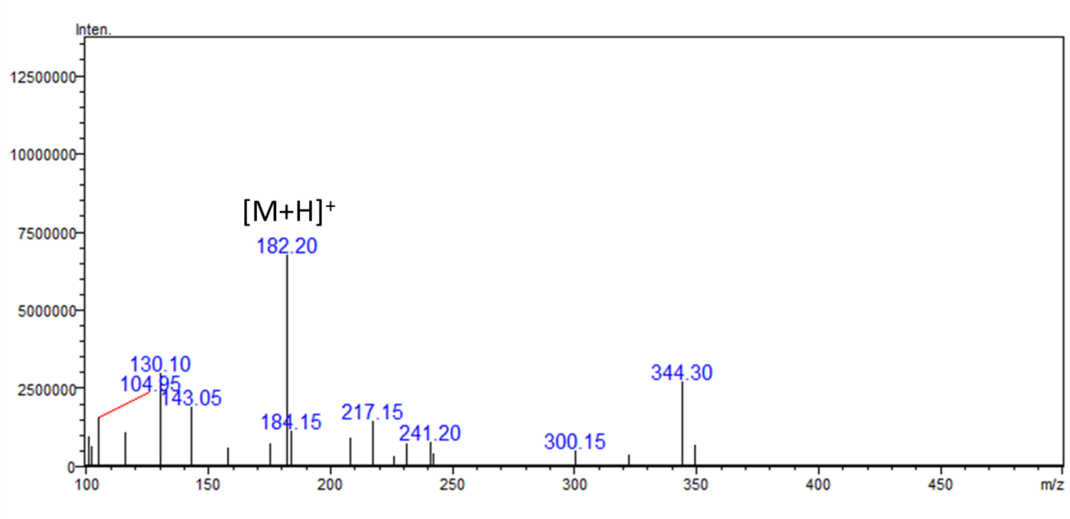

Figure S5. MS of **1c**#. MS calculated $[M+H]^+$: 182.14 Da, observed $[M+H]^+$: 182.20 Da.

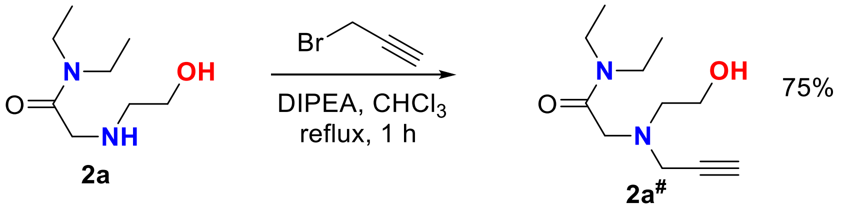

Scheme S5. The synthesis of mono alkyne from amine hydroxyl diethyl amide derivative.

Based on above general procedure,

N,N-diethyl-2-((2-hydroxyethyl)amino)acetamide **2a** (1.0 g, 5.73 mmol), DIPEA (2.0 mL, 11.47 mmol, 2 equiv.), propargyl bromide (0.9 mL, 11.47 mmol, 2 equiv.) afford mono-alkyne **2a#;** brown viscous liquid; yield: 920 mg (75%).

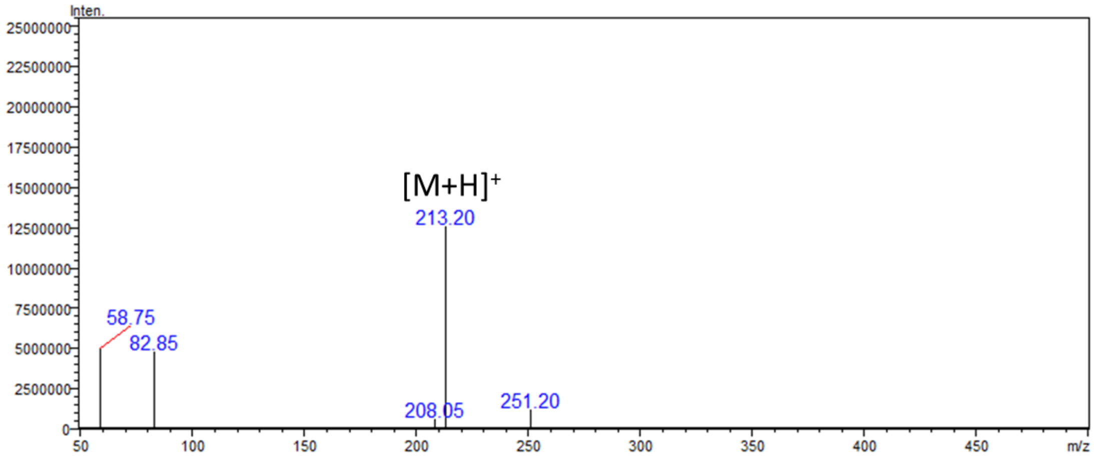

Figure S6. MS of **2a**#. MS calculated $[M+H]^+$: 213.15 Da, observed $[M+H]^+$: 213.20 Da.

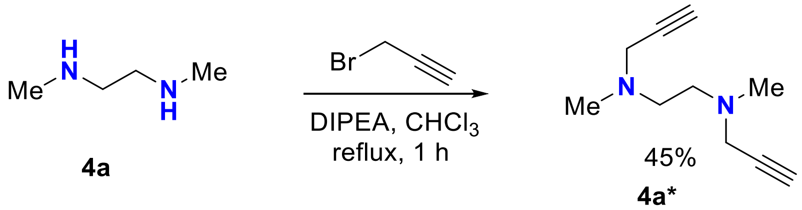

Scheme S6. The synthesis of bis-alkyne from N, N'-dimethyl ethylene diamine.

Based on above general procedure,

N,*N*′-Dimethylethylenediamine **4a** (500 mg, 5.67 mmol), DIPEA (3.6 mL, 22.68 mmol, 4 equiv.), propargyl bromide (1.7 mL, 22.68 mmol, 4 equiv.) afford di-alkyne **4a***; dark brown viscous liquid; yield: 420 mg (45%).

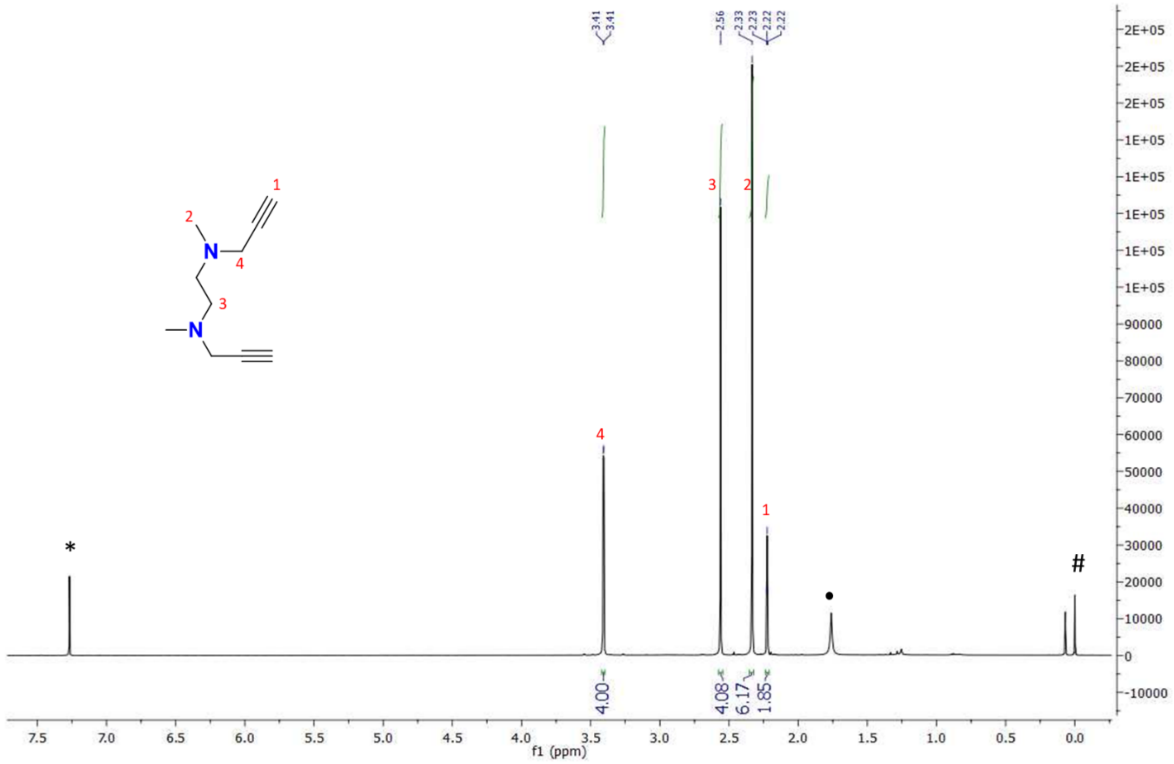

Figure S7. ^{1}H-NMR (500 MHz) of **4a*** in $CDCl_3$: δ (ppm) 2.22 (t, 2H), 2.33 (s, 6H), 2.56 (s, 4H), 3.41 (d, 4H), "#", "•" and "*" represent the residual proton of internal standard tetramethyl silane, THF and $CDCl_3$ respectively.

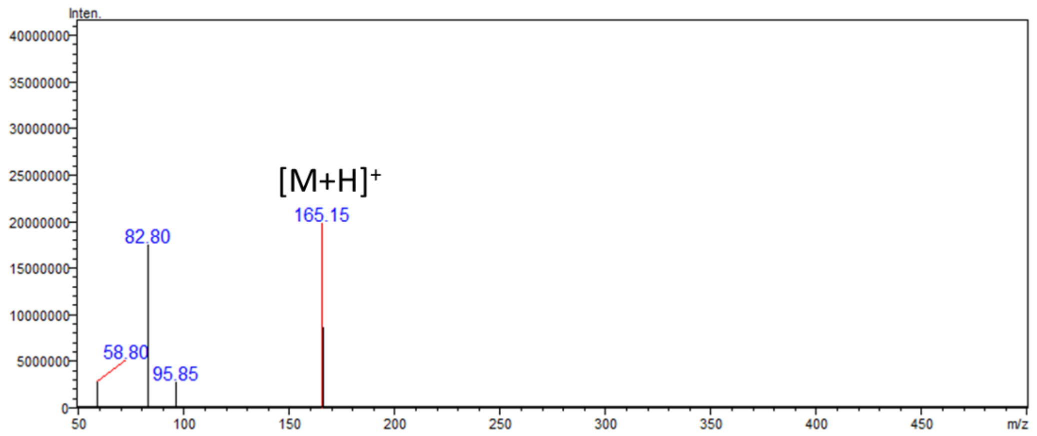

Figure S8. MS of **4a***. MS calculated $[M+H]^+$: 165.13 Da, observed $[M+H]^+$: 165.15 Da.

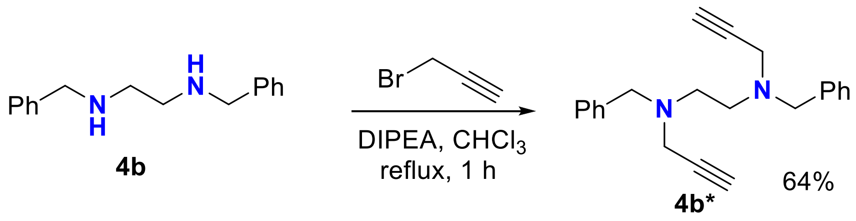

Scheme S7. The synthesis of bis-alkyne from N, N'-dibenzyl ethylene diamine.

Based on above general procedure,

N,*N'*-Dibenzylethylenediamine **4b** (1.0 g, 4.16 mmol), DIPEA (2.9 mL, 16.64 mmol, 4 equiv.), propargyl bromide (1.2 mL, 16.64 mmol, 4 equiv.) afford di-alkyne **4b*;** dark brown viscous liquid; yield: 850 mg (64%).

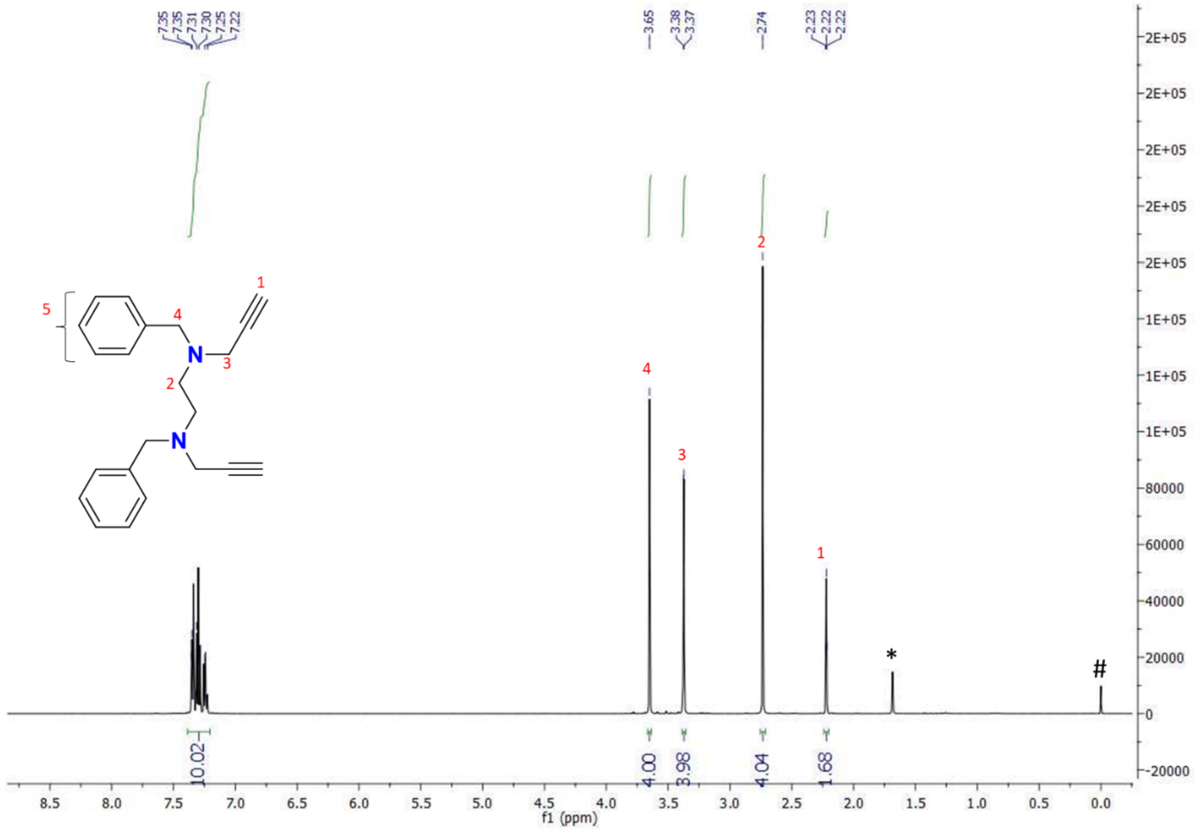

Figure S9. ^{1}H-NMR (500 MHz) of **4b*** in $CDCl_3$: δ (ppm) 2.22 (t, 2H), 2.74 (s, 4H), 3.38 (d, 4H), 3.65 (s, 4H), 7.30 (m, 10H), "#" and "*" represent the residual proton of internal standard tetramethyl silane and THF respectively.

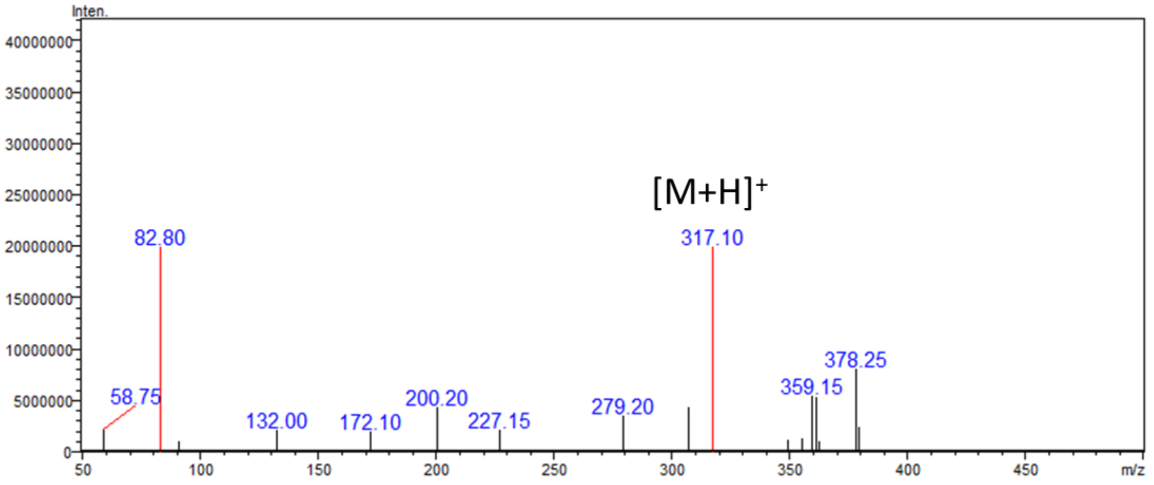

Figure S10. MS of **4b***. MS calculated $[M+H]^+$: 317.19 Da, observed $[M+H]^+$: 317.10 Da.

6. Preparation of bis-alkynes: General procedure for O-propargylation

To a suspension of NaH (80-270 mg, 3.30-11.41 mmol, 2-4 equiv.) in DMF (1-5 mL), diols and amino-alcohols **1a, 1b, 1c, 3a** and **5a** (300-700 mg, 1.65-3.54 mmol, 1 equiv.) were added at 0 °C. Then the reaction mixture was stirred for 10 min at room temperature. Later on, propargyl bromide (0.3-0.9 mL, 3.30-11.41 mmol, 2-4 equiv.) was added dropwise and then stirring was continued for 2-12 h. At the end of the reaction by TLC analysis, the reaction mixture was quenched with saturated aq. NH_4Cl and the resulting aqueous layer was extracted with EtOAc. The combined organic layers were washed with brine and dried over anhydrous Na_2SO_4. The solvent was evaporated under reduced pressure and the resulting crude residue was subjected to silica gel column chromatography using 0-40% EtOAc/petroleum ether as an eluent to afford pure di-alkynes.

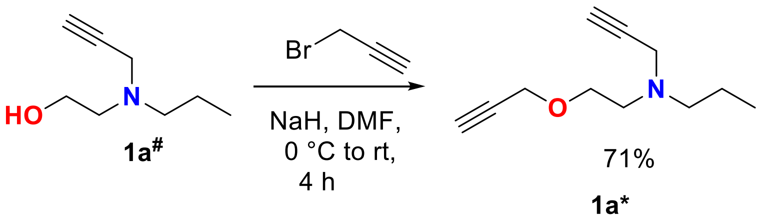

Scheme S8. The synthesis of bis-alkyne from propyl substituted mono alkyne.

Based on above general procedure,

2-(prop-2-yn-1-yl(propyl)amino)ethan-1-ol **1a#** (300 mg, 2.12 mmol), NaH (102 mg, 4.24 mmol, 2 equiv.), propargyl bromide (0.4 mL, 4.24 mmol, 2 equiv.) afford bis-alkyne **1a***. colourless viscous liquid; yield: 270 mg (71%).

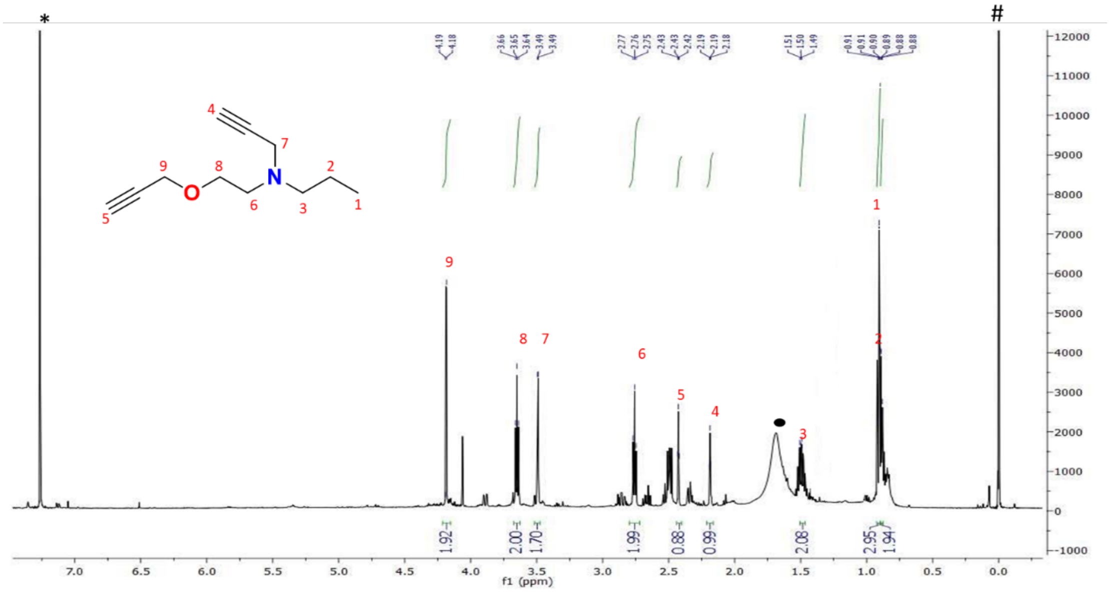

Figure S11. ^{1}H-NMR (500 MHz) of **1a*** in $CDCl_3$: δ (ppm) 0.88 (t, 3H), 0.91 (m, 2H), 1.50 (t, 2H), 2.19 (t, 1H), 2.43 (t, 1H), 2.76 (t, 2H), 3.49 (d, 2H), 3.65 (t, 2H), 4.18 (d, 2H), "#", "•" and "*" represent the residual proton of internal standard tetramethyl silane, THF and $CDCl_3$ respectively.

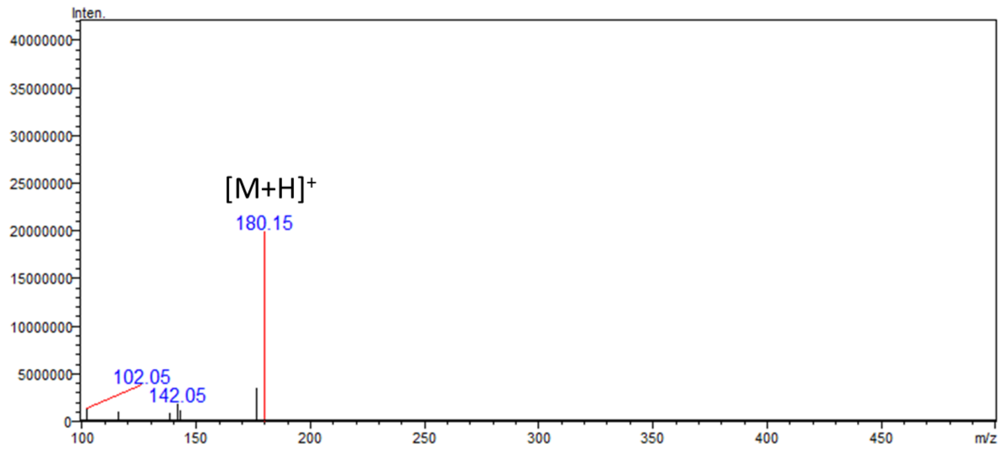

Figure S12. MS of **1a***. MS calculated $[M+H]^+$: 180.13 Da, observed $[M+H]^+$: 180.15 Da.

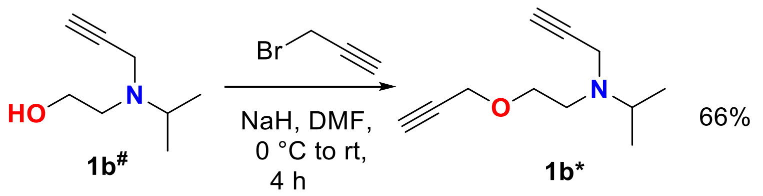

Scheme S9. The synthesis of bis-alkyne from isopropyl substituted mono alkyne.

Based on above general procedure,

2-(isopropyl(prop-2-yn-1-yl)amino)ethan-1-ol **1b**# (500 mg, 3.54 mmol), NaH (170 mg, 7.08 mmol, 2 equiv.), propargyl bromide (0.6 mL, 7.08 mmol, 2 equiv.) afford bis-alkyne **1b***. colourless viscous liquid; yield: 420 mg (66%).

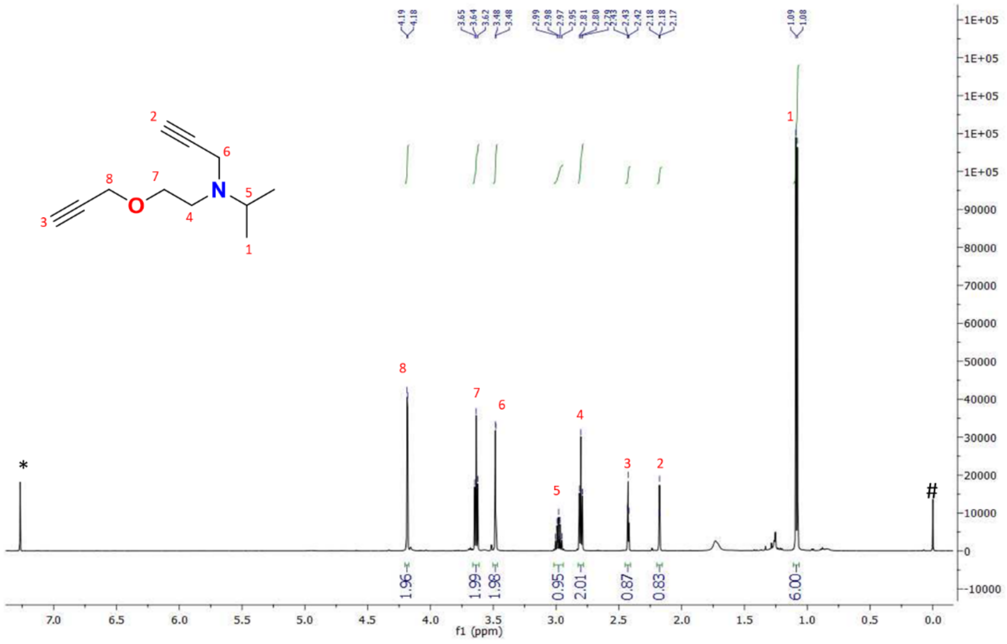

Figure S13. ^{1}H-NMR (500 MHz) of **1b*** in $CDCl_3$: δ (ppm) 1.09 (d, 6H), 2.18 (t, 2H), 2.43 (t, 3H), 2.80 (t, 4H), 2.97 (m, 5H), 3.48 (d, 2H), 3.64 (t, 2H), 4.18 (d, 2H), "#" and "*" represent the residual proton of internal standard tetramethyl silane and $CDCl_3$ respectively.

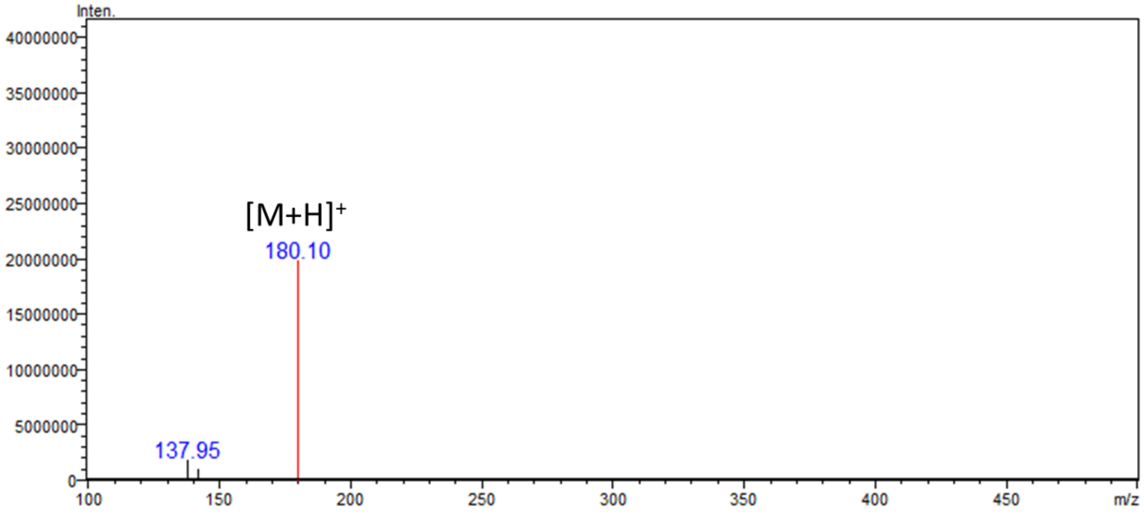

Figure S14. MS of **1b***. MS calculated $[M+H]^+$: 180.13 Da, observed $[M+H]^+$: 180.10 Da.

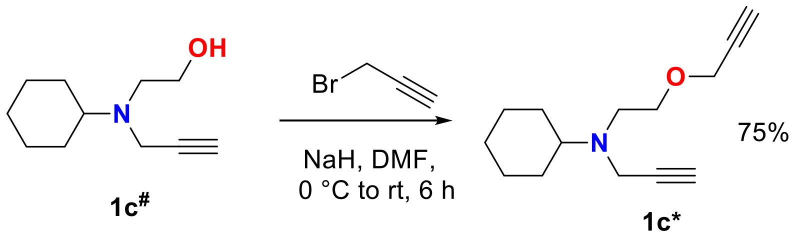

Scheme S10. The synthesis of bis-alkyne from cyclohexyl substituted mono alkyne.

Based on above general procedure,

2-(cyclohexyl(prop-2-yn-1-yl)amino)ethan 1-ol **1c#** (300 mg, 1.65 mmol), NaH (80 mg, 3.30 mmol, 2 equiv.), propargyl bromide (0.3 mL, 3.30 mmol, 2 equiv.) afford bis-alkyne **1c***. colourless viscous liquid; yield: 270 mg (75%).

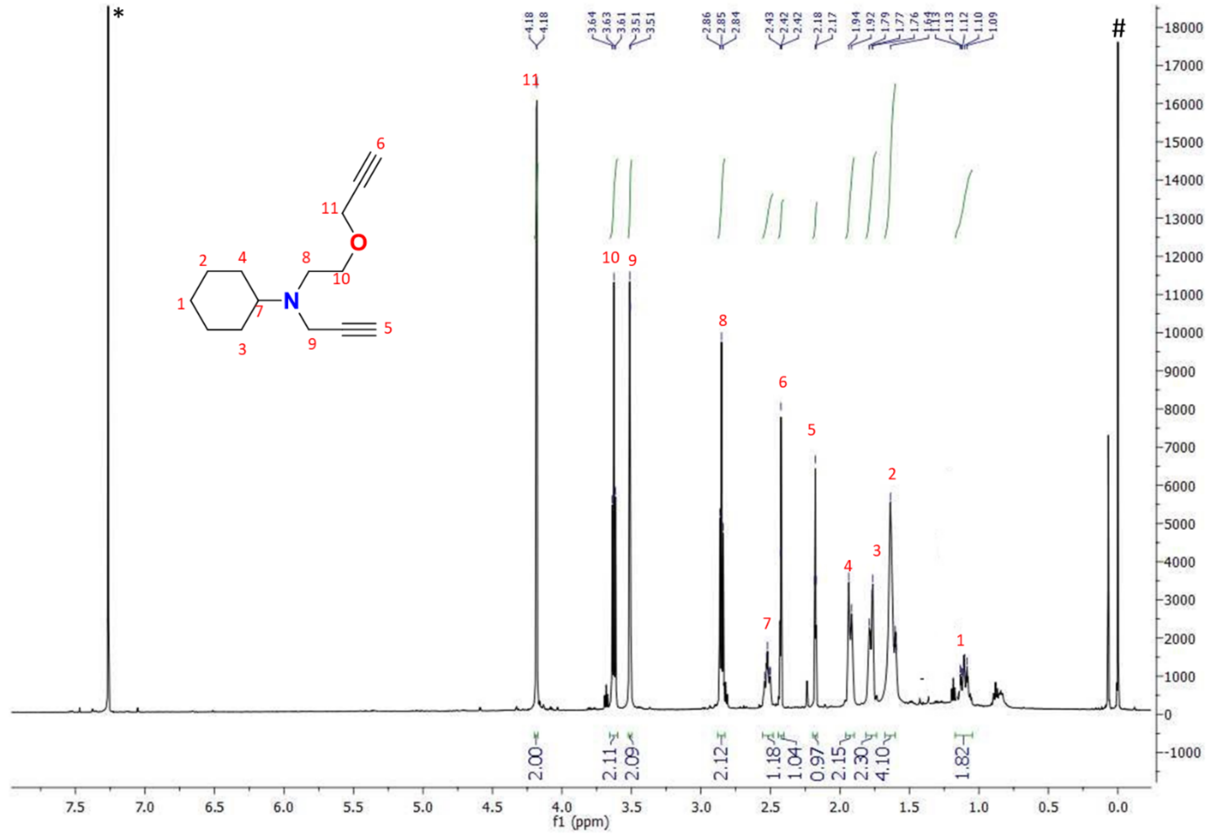

Figure S15. ^{1}H-NMR (500 MHz) of **1c*** in $CDCl_3$: δ (ppm) 1.12 (m, 2H), 1.76 (m, 4H), 1.79 (m, 2H), 1.94 (m, 2H), 2.18 (t, 1H), 2.42 (t, 1H), 2.85 (t, 2H), 3.51 (d, 2H), 3.63 (t, 2H), 4.18 (d, 2H), "#" and "*" represent the residual proton of internal standard tetramethyl silane and $CDCl_3$ respectively.

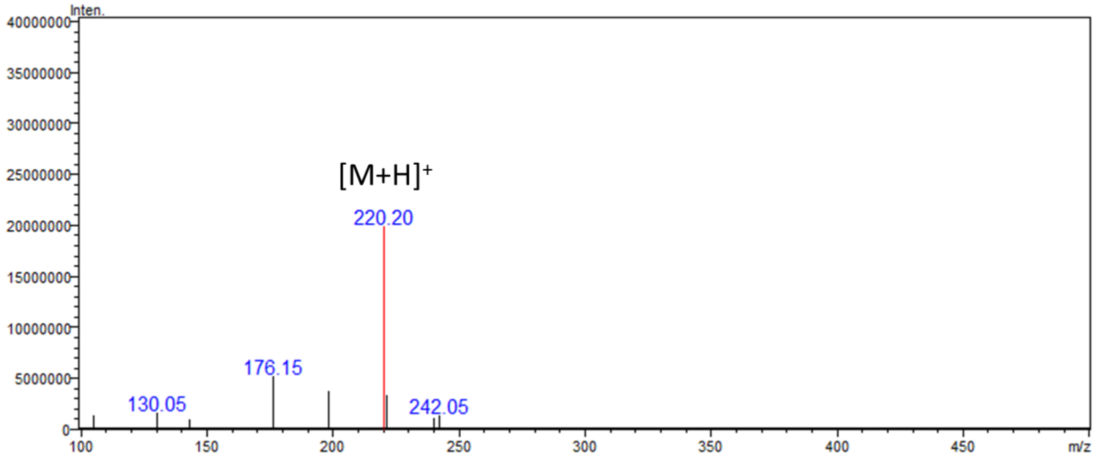

Figure S16. MS of **1c***. MS calculated $[M+H]^+$: 220.16 Da, observed $[M+H]^+$: 220.20 Da.

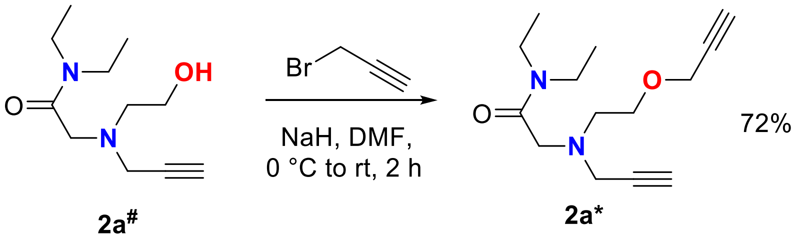

Scheme S11. The synthesis of bis-alkyne from diethyl amide substituted mono alkyne.

Based on above general procedure,

N,N-diethyl-2-((2-hydroxyethyl)(prop-2-yn-1-yl)amino)acetamide **2a#** (500 mg, 2.35 mmol), NaH (115 mg, 4.70 mmol, 2 equiv.), propargyl bromide (0.4 mL, 4.70 mmol, 2 equiv.) afford bis-alkyne **2a*;** brown viscous liquid; yield: 425 mg (72%).

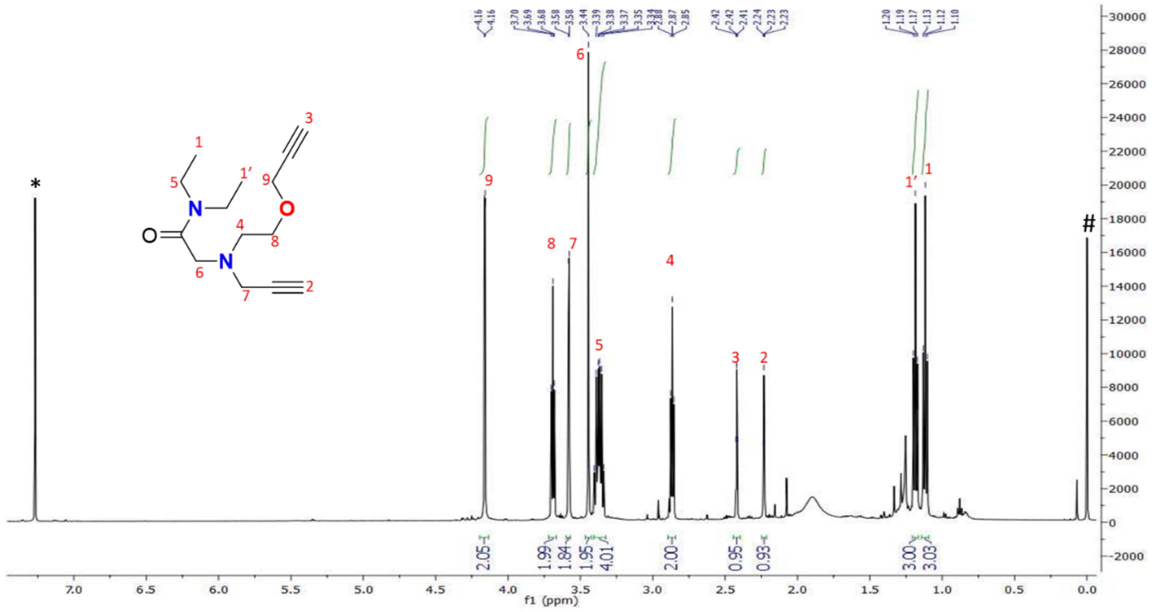

Figure S17. ^{1}H-NMR (500 MHz) of **2a*** in $CDCl_3$: δ (ppm) 1.12 (t, 3H), 1.19 (t, 3H), 2.23 (t, 1H), 2.42 (t, 1H), 2.87 (t, 2H), 3.37 (m, 4H), 3.44 (s, 2H), 3.58 (d, 2H), 3.69 (t, 2H), 4.16 (d, 2H), "#" and "*" represent the residual proton of internal standard tetramethyl silane and $CDCl_3$ respectively.

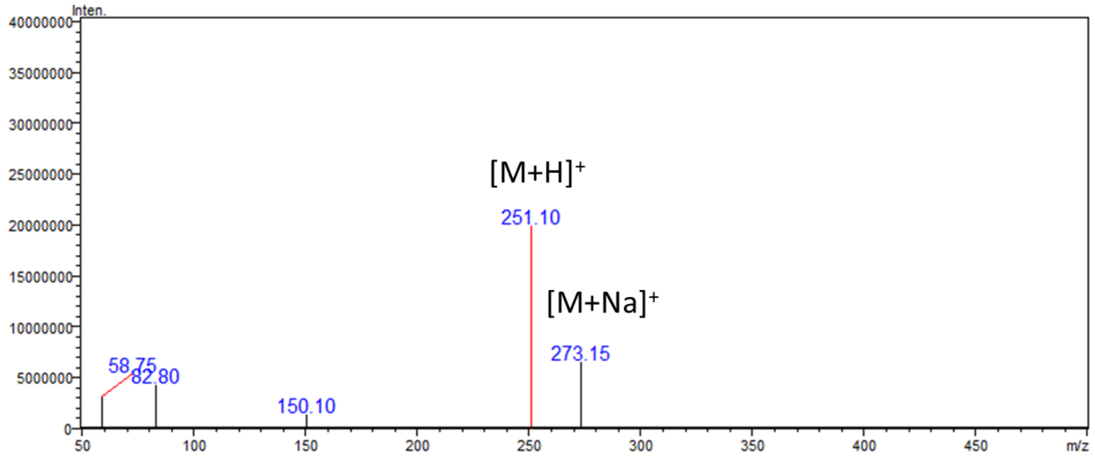

Figure S18. MS of **2a***. MS calculated $[M+H]^+$: 251.16 Da, observed $[M+H]^+$: 251.10 Da; calculated $[M+Na]^+$: 273.16 Da, observed $[M+H]^+$: 273.15 Da.

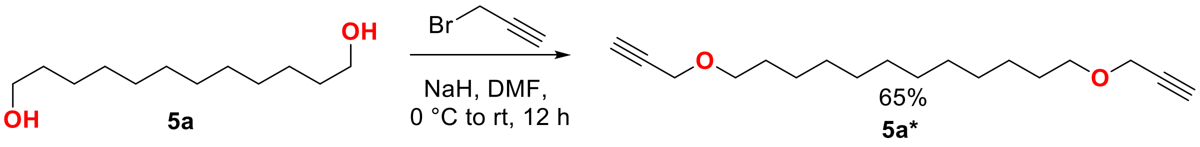

Scheme S12. The synthesis of bis-alkyne from dodecane 1,12-diol.

Based on above general procedure,

dodecane-1,12-diol (500 mg, 2.47 mmol) **5a**, NaH (240 mg, 9.88 mmol, 4 equiv.), propargyl bromide (0.8 mL, 9.88 mmol, 4 equiv.) afford bis-alkyne **5a***. light brown viscous liquid; yield: 450 mg (65%).

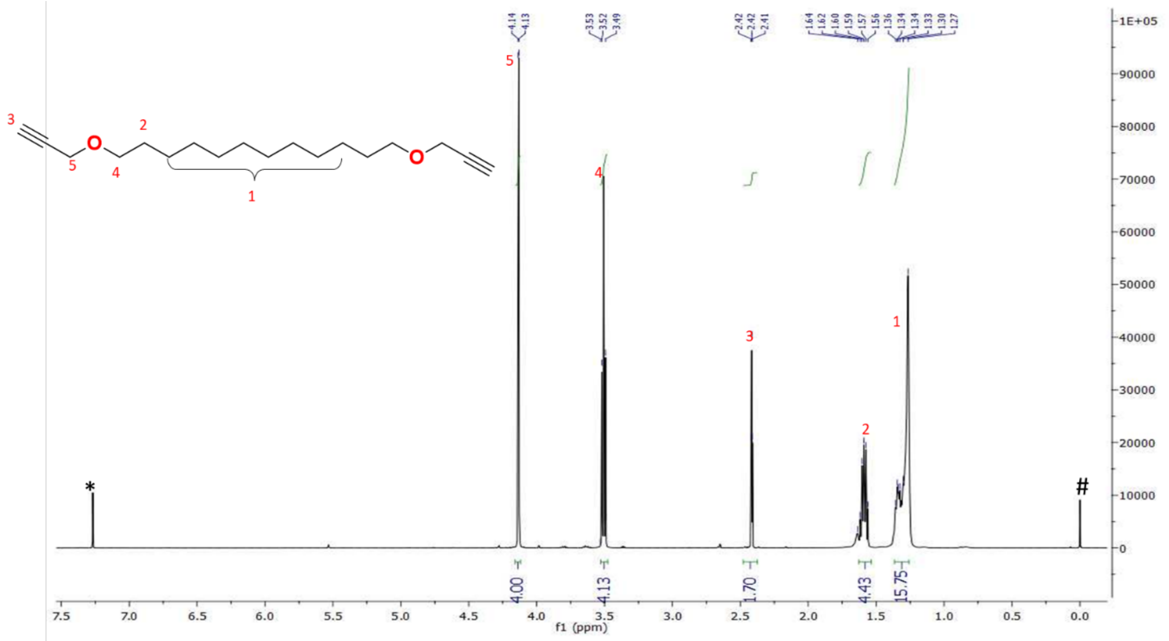

Figure S19. ^{1}H-NMR (500 MHz) of **5a*** in $CDCl_3$: δ (ppm) 1.27-1.36 (m, 16H), 1.60 (m, 4H), 2.42 (m, 2H), 3.52 (t, 4H), 4.14 (d, 4H), "#" and "*" represent the residual proton of internal standard tetramethyl silane and $CDCl_3$ respectively.

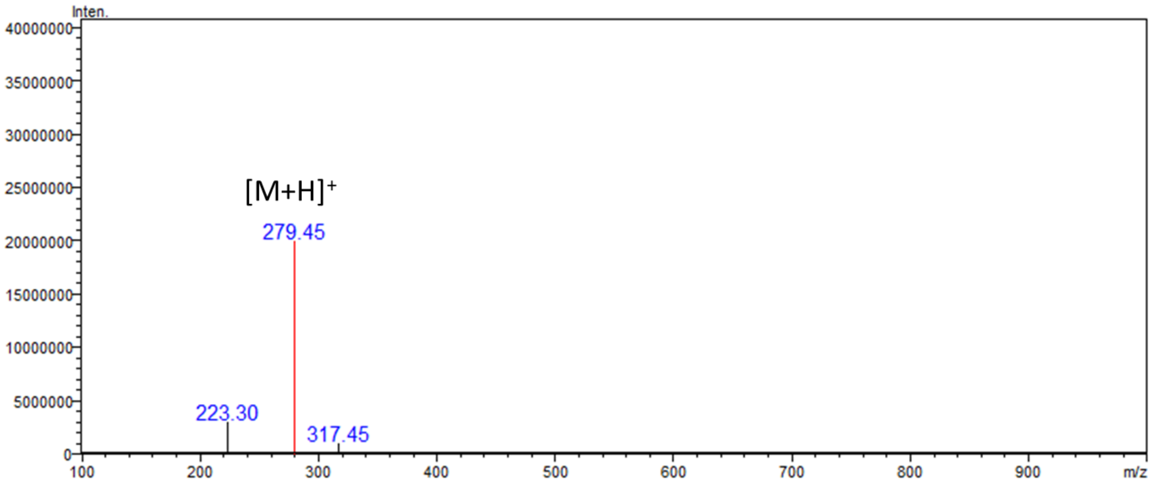

Figure S20. MS of **5a***. MS calculated $[M+H]^+$: 279.22 Da, observed $[M+H]^+$: 279.45 Da.

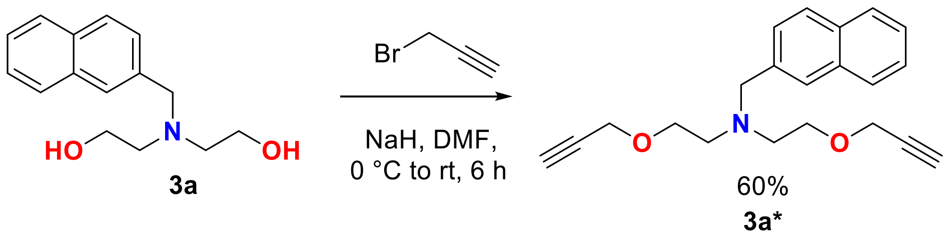

Scheme S13. The synthesis of bis-alkyne from 2-methyl naphthalene substituted diethanolamine.

Based on above general procedure,

2,2'-((naphthalen-2-ylmethyl)azanediyl)bis(ethan-1-ol) **3a** (700 mg, 2.85 mmol), NaH (275 mg, 11.41 mmol, 4 equiv.), propargyl bromide (0.9 mL, 11.41 mmol, 4 eqvuiv.) afford bis-alkyne **3a***. Pale brown viscous liquid; yield: 550 mg (60%).

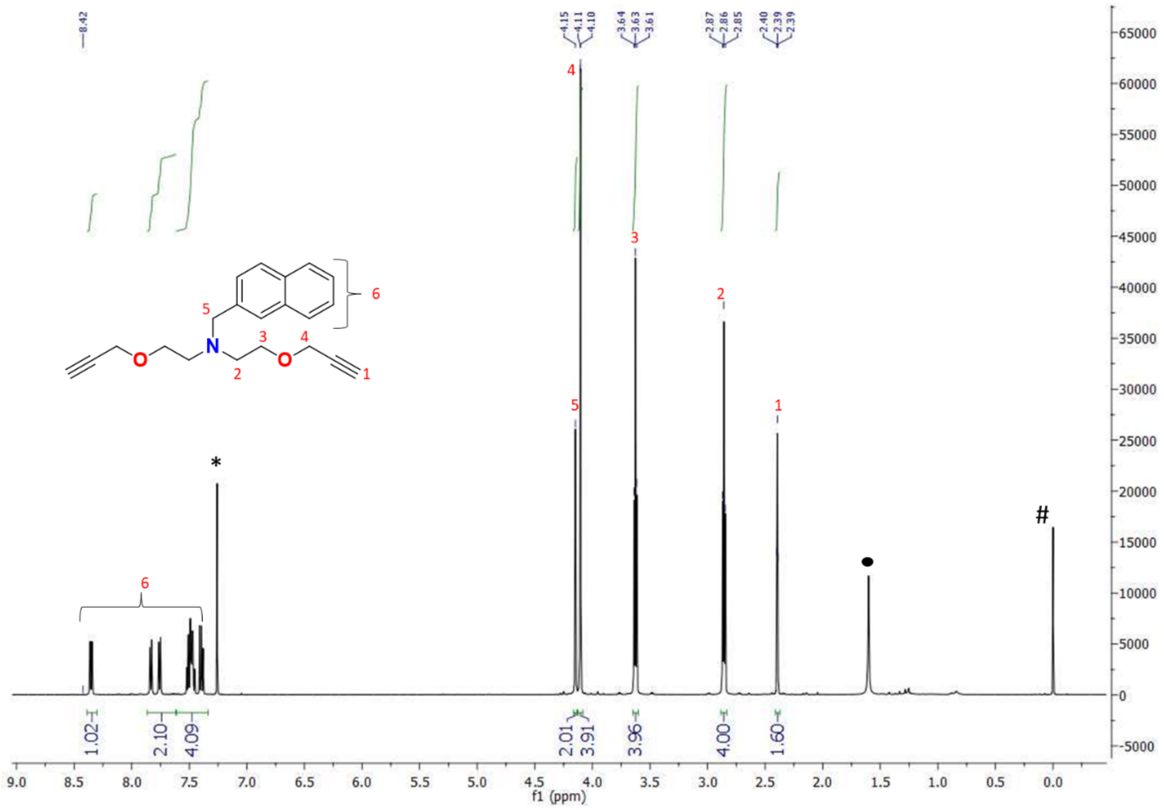

Figure S21. ^{1}H-NMR (500 MHz) of **3a*** in $CDCl_3$: δ (ppm) 2.39 (s, 2H), 2.86 (t, 4H), 3.63 (t, 4H), 4.11 (d, 4H), 4.15 (s, 2H), 7.45-8.5 (m, 7H), “#”, “ • ” and “*” represent the residual proton of internal standard tetramethyl silane, THF and $CDCl_3$ respectively.

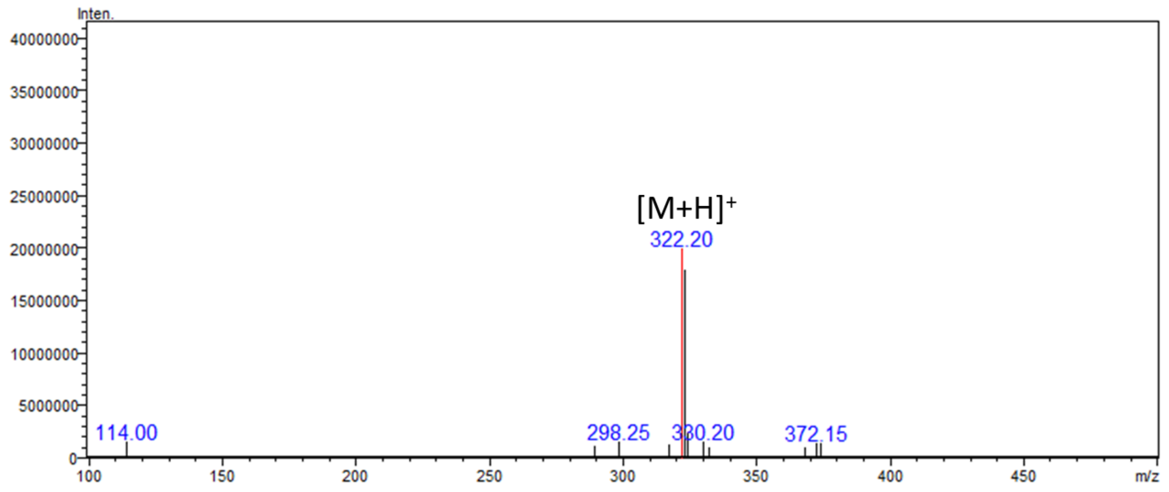

Figure S22. MS of **3a***. MS calculated $[M+H]^+$: 322.17 Da, observed $[M+H]^+$: 322.20 Da.

7. Synthesis of triazole based macrocycles: General procedure for azide-alkyne click

To stirred solution of diazide (180-560 mg, 1.19-3.62 mmol, 1 equiv.) and bis-alkyne (200-800 mg, 1.19-3.62 mmol, 1 equiv.) derivatives in degassed ACN (25-100 mL) CuI (0.12-0.36 mmol, 0.1 equiv.) and DIPEA (3.57-10.86 mmol, 3 equiv.) in de-gassed ACN (25 mL) were added dropwise over 10 min at room temperature under nitrogen atmosphere. The resulting mixture was stirred at this temperature for 16-24 h. The resulting reaction mixture was extracted with $CH_2Cl_2/CHCl_3$. The combined organic layers were washed with aqueous brine, dried over anhydrous Na_2SO_4 and concentrated under vacuo. The crude residue was purified through column chromatography with a 60-120 mesh silica gel by elution with 0-10 % MeOH in EtOAc to yield the respective pure macrocycles 1-8 containing bis-triazoles with good yields.

Macrocycle 1

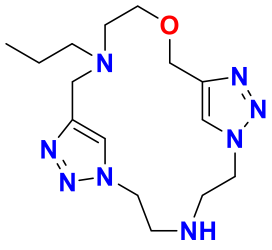

Based on above general procedure,

Diazide **1a*** (560 mg, 3.62 mmol) and di-alkyne (650 mg, 3.62 mmol) afford **MC1**. Colourless viscous liquid; yield: 900 mg (74%).

^{1}H-NMR (500 MHz) of **MC1** in $CDCl_3$: δ (ppm) 0.91 (t, 3H), 1.27 (m, 2H), 1.57 (t, 2H), 2.56 (t, 4H), 3.06 (d, 2H), 3.12 (t, 4H), 3.71 (t, 2H), 4.47 (s, 2H), 4.67 (s, 2H), 7.49 (s, 1H), 7.67 (s, 1H). "*" represents the residual proton of $CDCl_3$. ^{13}C NMR (125 MHz, CDCl3) δ (ppm) 11.72, 19.83, 48.76, 50.24, 53.27, 55.29, 57.88, 58.33, 64.69, 69.02, 124.04, 124.77, 144.28 and 145.30. LC-MS calculated $[M+H]^+$: 335.23 Da, observed $[M+H]^+$: 335.55 Da. HRMS(ESI) m/z calculated for $C_{15}H_{26}N_8O$ $[M+H]^+$: 335.2308 Da; found $[M+H]^+$: 335.2303 Da. FT-IR (ATR) $\tilde{\upsilon}$ = 3333 cm^{-1} (stretching, N-H), 2951 cm^{-1} (stretching, alkene C-H), 2102 (stretching, N=N-N) and 1649 cm^{-1} (stretching, C=C)

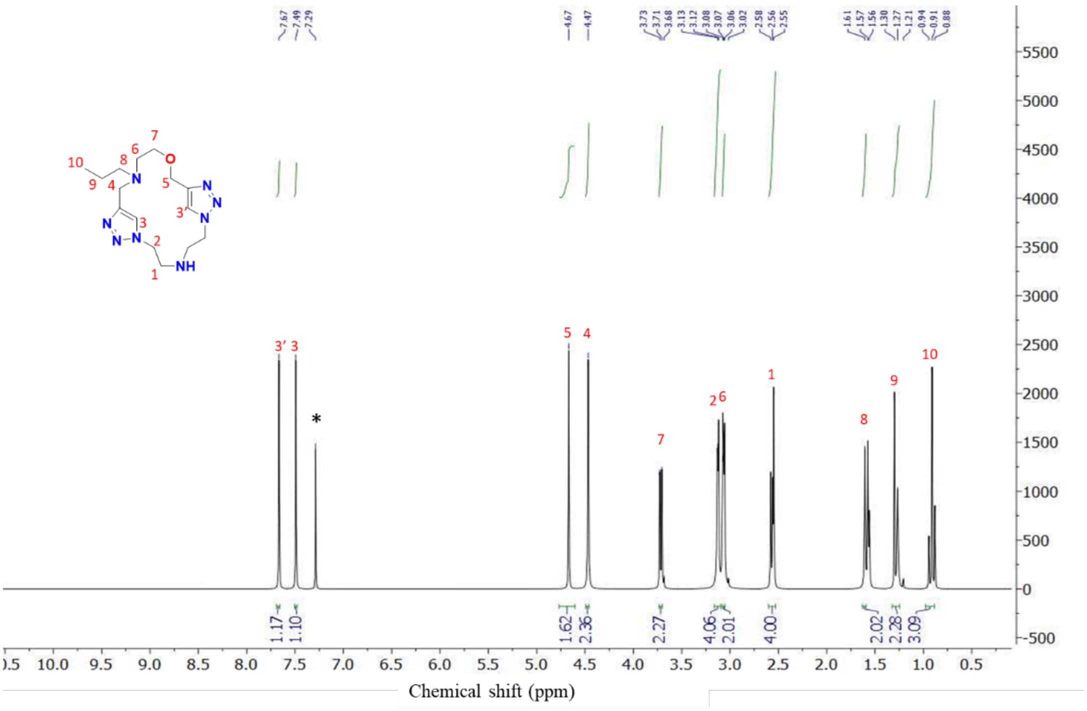

Figure S23. ^{1}H-NMR (500 MHz) of **MC1** in $CDCl_3$.

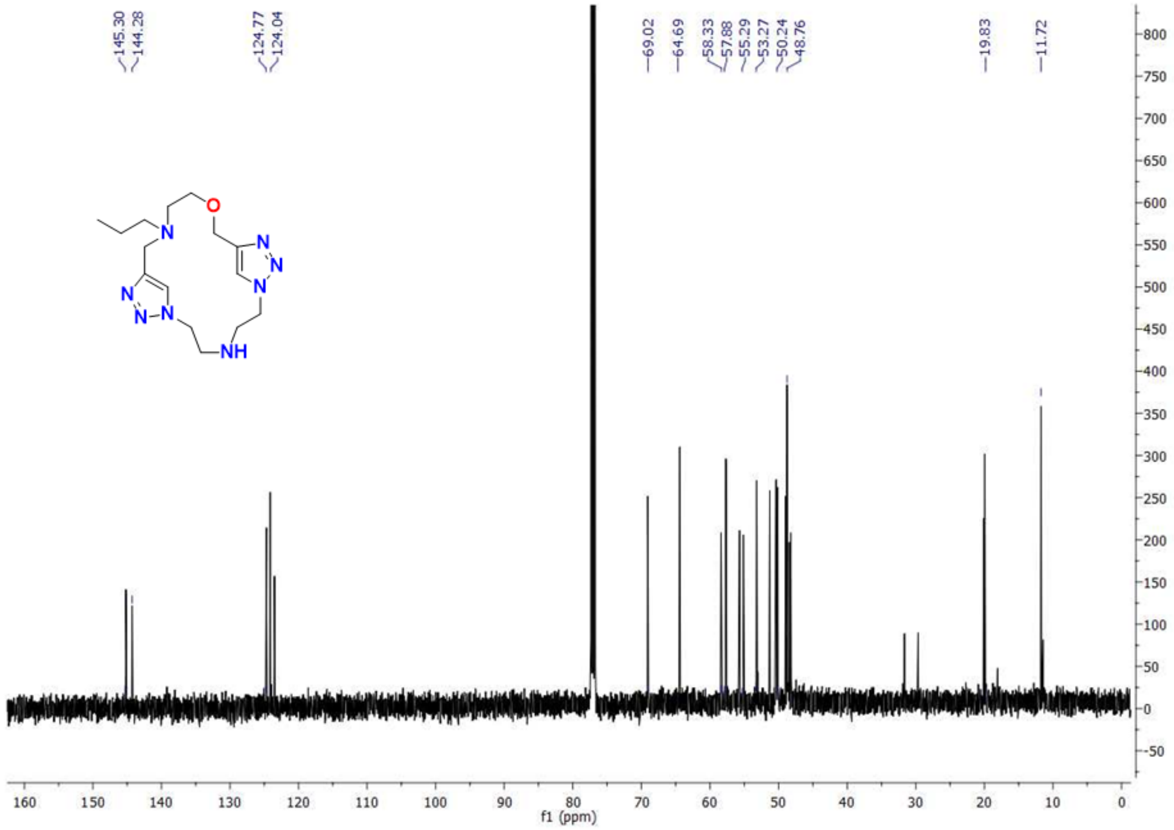

Figure S24. ^{13}C-NMR of **MC1** in $CDCl_3$.

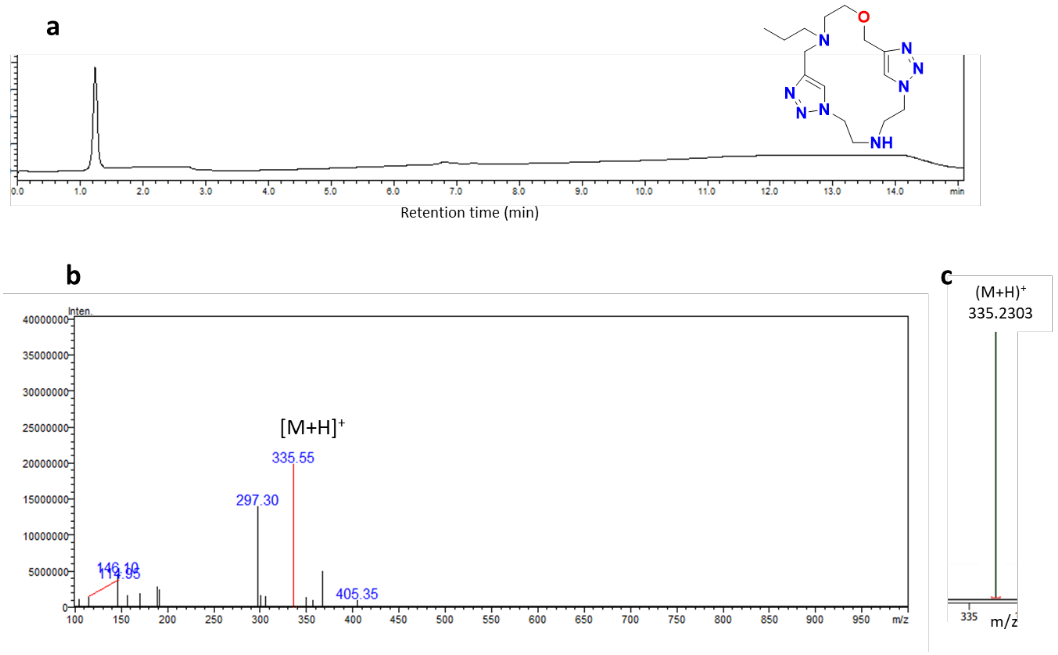

Figure S25. a: HPLC of **MC1** eluted in acetonitrile and water gradient recorded at 210 nm. **b**: Full range MS spectrum in positive ion mode. **c**: HRMS with $[M+H]^+$ peak.

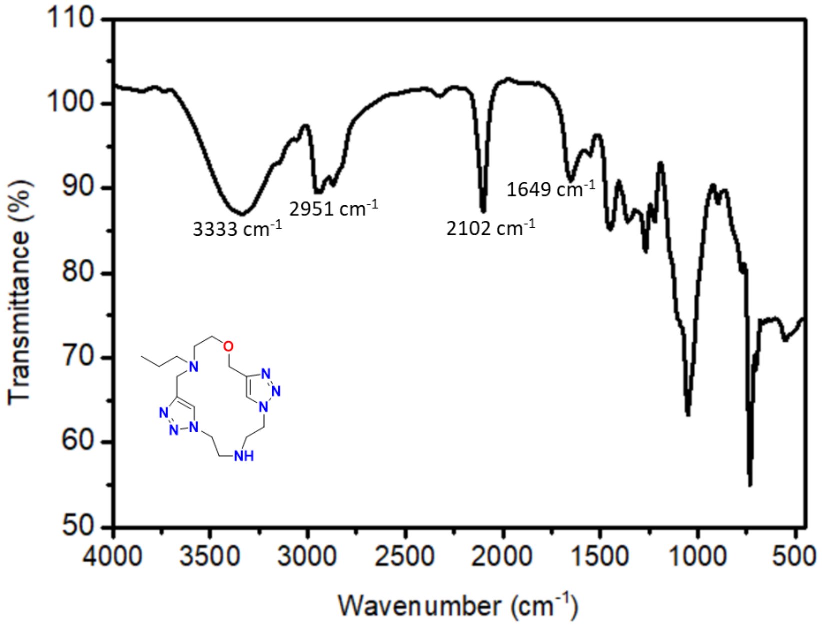

Figure S26. FT-IR spectrum of **MC1**.

Macrocycle 2

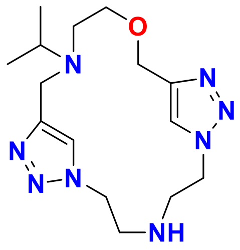

Based on above general procedure,

Diazide **1b*** (430 mg, 2.78 mmol) and di-alkyne (500 mg, 2.78 mmol) afford **MC2**. Colourless viscous liquid; yield: 750 mg (80%).

^{1}H-NMR (500 MHz) of **MC2** in $CDCl_3$: δ (ppm) 1.13 (m, 3H), 1.25 (m, 3H), 2.83 (t, 2H), 3.08 (m, 8H), 3.97 (t, 3H), 3.62 (t, 2H), 3.9 (s, 2H), 4.46 (t, 2H), 7.58 (s, 1H), 7.72 (s, 1H). “#” and “*” represent the residual proton of internal standard tetramethyl silane and $CDCl_3$ respectively. ^{13}C NMR (125 MHz, CDCl3) δ (ppm) 18.07, 48.86, 50.38, 51. 04, 53.25, 57.67, 63.49, 64.23, 69.01, 123..61, 124.74, 144.49 and 145.24. LC-MS calculated $[M+H]^+$: 335.23 Da, observed $[M+H]^+$: 335.20 Da, $[M+Na]^+$: 357.25. HRMS(ESI) m/z calculated for $C_{15}H_{26}N_8O$ $[M+H]^+$: 335.2308 Da; found $[M+H]^+$: 335.2473 Da. FT-IR (ATR) $\tilde{\upsilon}$ = 3368 cm$^-$

^{1} (stretching, N-H), 2951 cm^{-1} (stretching, alkene C-H), 2102 (stretching, N=N-N) and 1635 cm^{-1} (stretching, C=C)

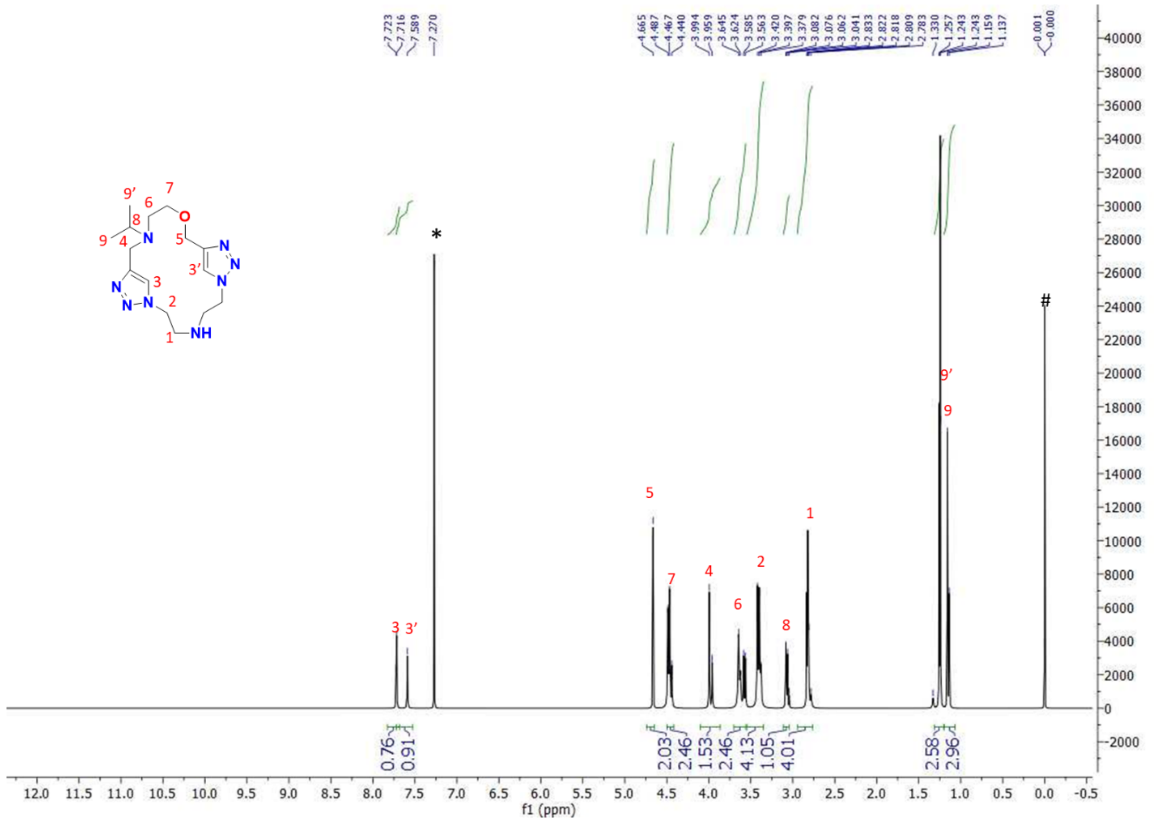

Figure S27. ^{1}H-NMR (500 MHz) of **MC2** in $CDCl_3$.

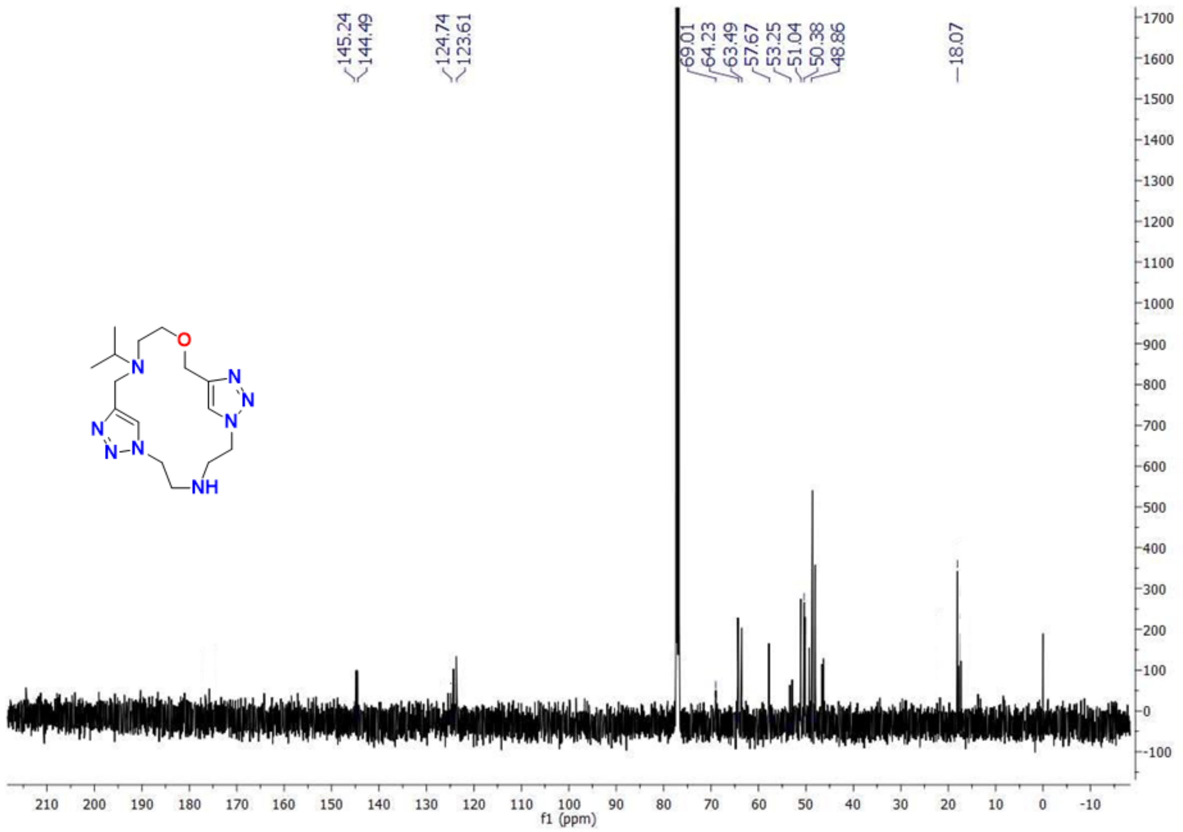

Figure S28. ^{13}C-NMR of **MC2** in $CDCl_3$.

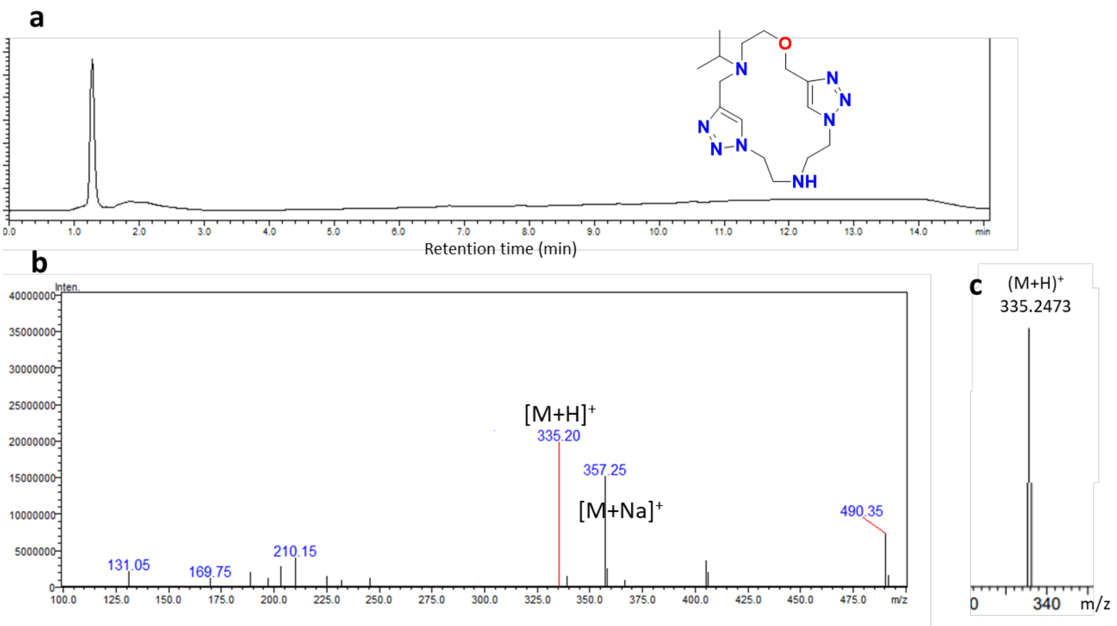

Figure S29. a: HPLC of **MC2** eluted in acetonitrile and water gradient recorded at 210 nm. **b**: Full range MS spectrum in positive ion mode. **c**: HRMS with $[M+H]^+$ peak.

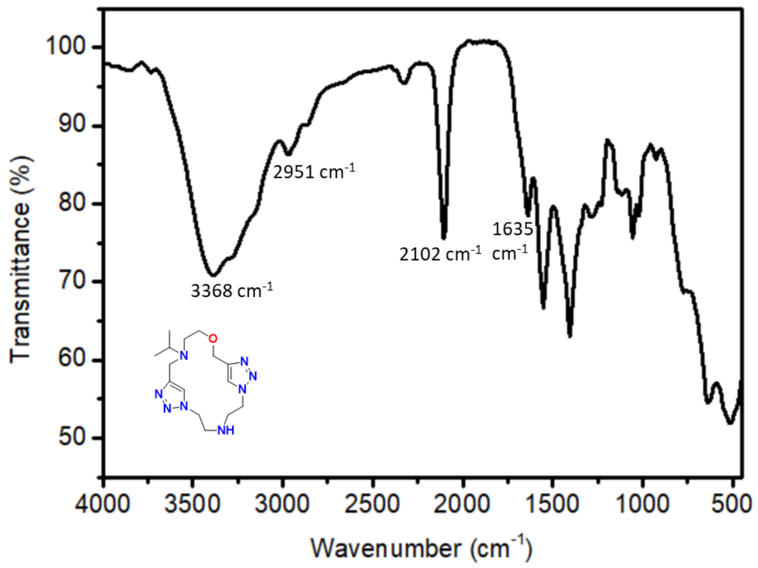

Figure S30. FT-IR spectrum of **MC2**.

Macrocycle 3

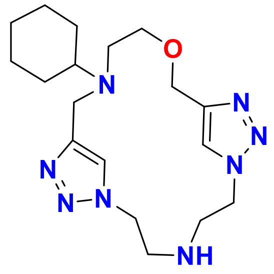

Based on above general procedure,

Diazide **1c*** (350 mg, 2.27 mmol) and di-alkyne (500 mg, 2.27 mmol) afford **MC3**. Brown viscous liquid; yield: 750 mg (88%).

^{1}H-NMR (500 MHz) of **MC3** in $CDCl_3$: δ (ppm) 0.84 (m, 2H), 1.25 (m, 4H), 2.24 (m, 4H), 2.43 (t, 4H), 2.83 (t, 4H), 3.10 (t, 2H), 3.49 (t, 3H), 3.66 (t, 2H), 4.18 (s, 2H), 4.64 (s, 2H), 7.4 (s, 1H), 7.58 (s, 1H). "*" represents the residual proton of $CDCl_3$. ^{13}C NMR (125 MHz, CDC13) δ (ppm) 20.15, 29.78, 31.82, 42.97, 44.63, 49.04, 50.26, 52.59, 58.66, 64.69, 67.89, 123.98, 124.73, 140.37, 140.02. LC-MS calculated $[M+H]^+$: 375.25 Da, observed $[M+H]^+$: 375.35 Da. HRMS(ESI) m/z calculated for $C_{18}H_{30}N_8O$ $[M+H]^+$: 375.2621 Da; found $[M+H]^+$: 375.2613 Da. FT-IR (ATR) $\upsilon^{\sim}$ = 3321 cm^{-1} (stretching, N-H), 2951 cm^{-1} (stretching, alkene C-H), 2095 (stretching, N=N-N) and 1664 cm^{-1} (stretching, C=C)

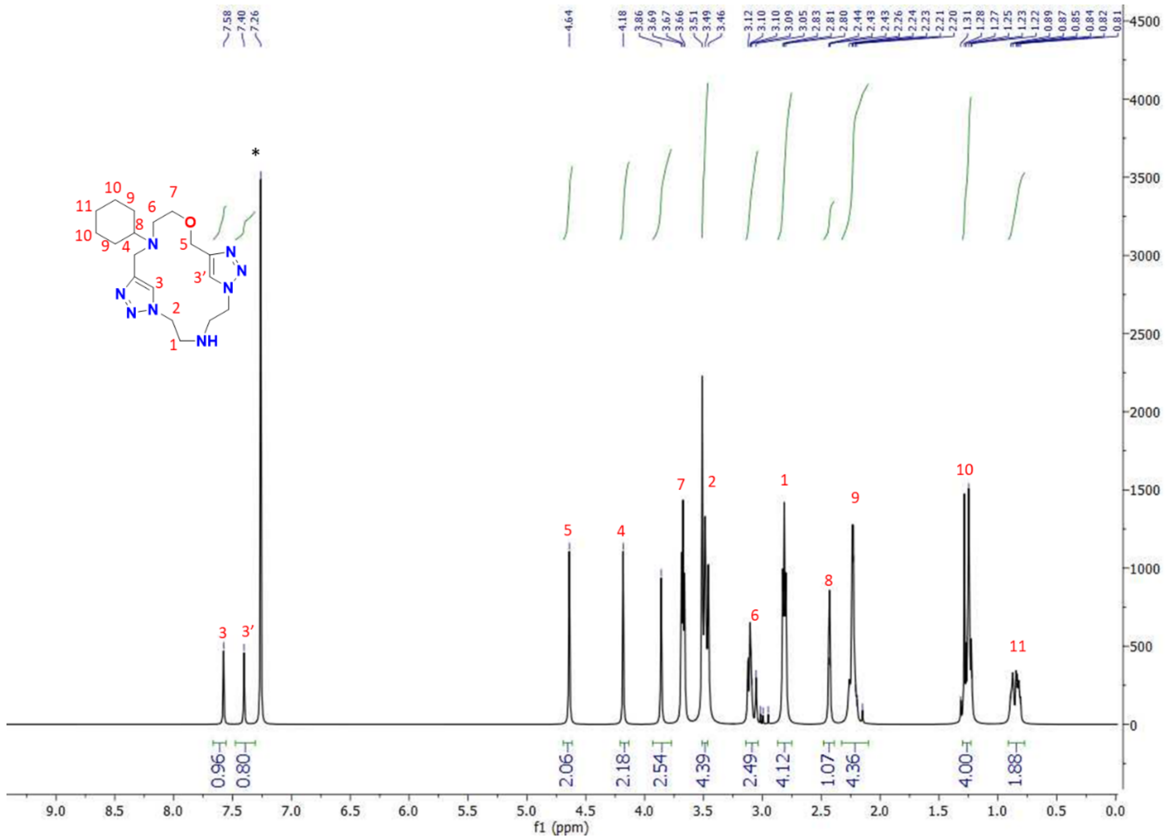

Figure S31. ^{1}H-NMR (500 MHz) of**MC3** in $CDCl_3$.

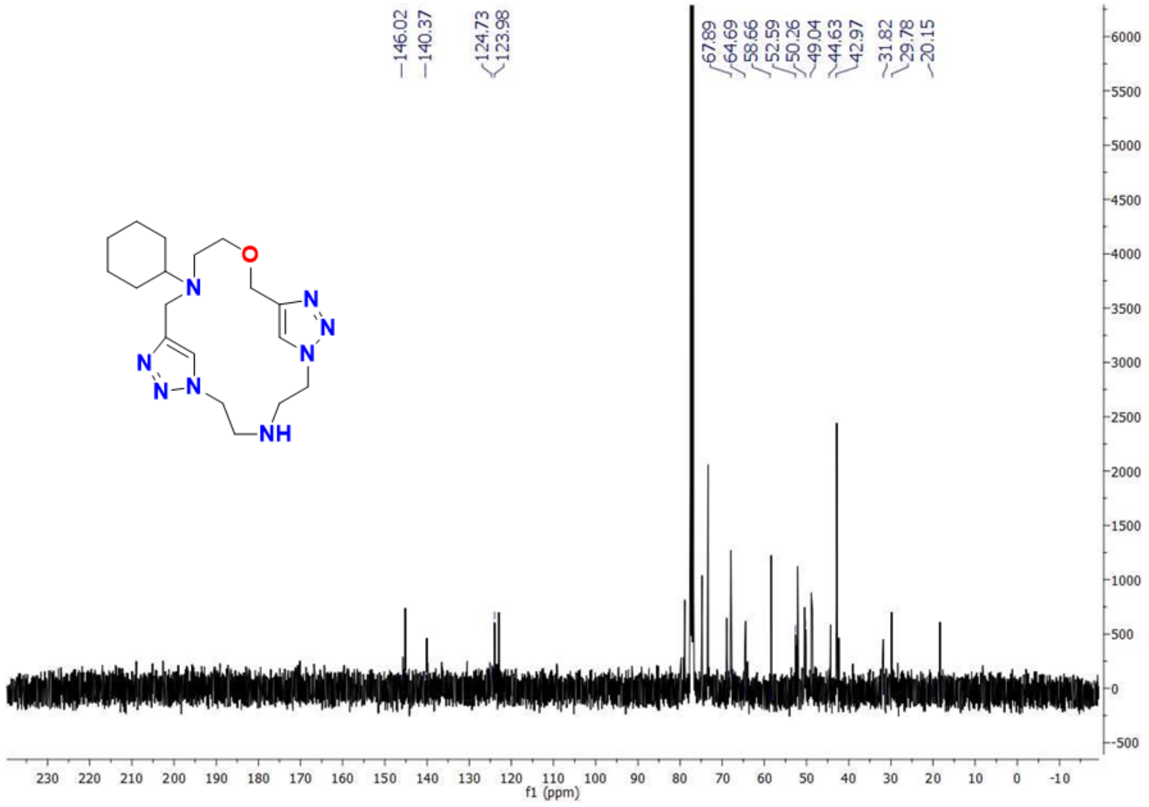

Figure S32. ^{13}C-NMR of **MC3** in $CDCl_3$.

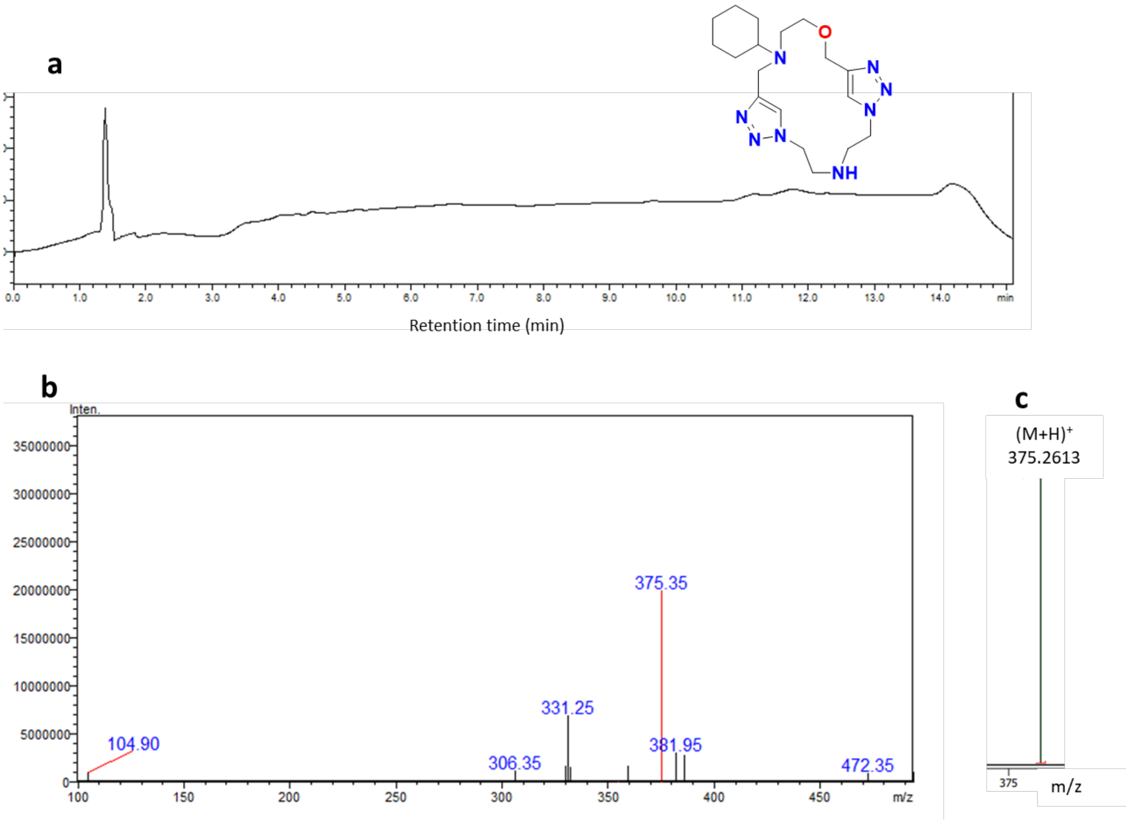

Figure S33 a: HPLC of **MC3** eluted in acetonitrile and water gradient recorded at 210 nm. **b**: Full range MS spectrum in positive ion mode. **c:** HRMS with $[M+H]^+$ peak.

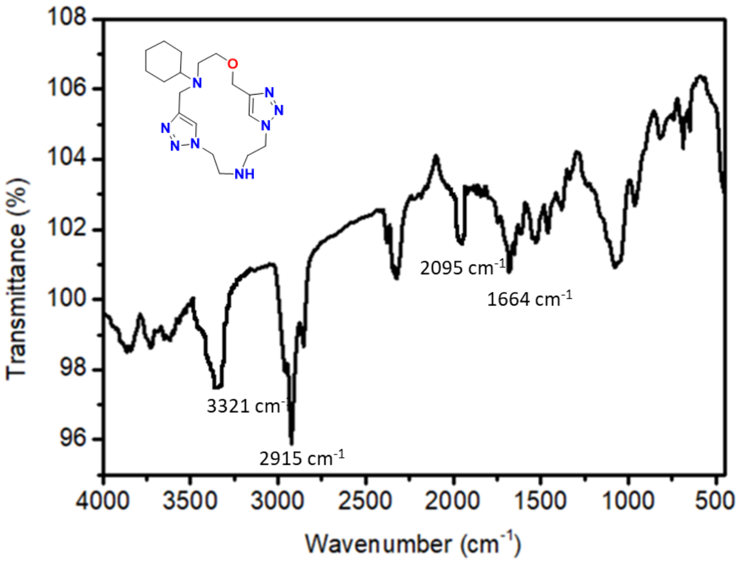

Figure S34. FT-IR spectrum of **MC3**.

Macrocycle 4

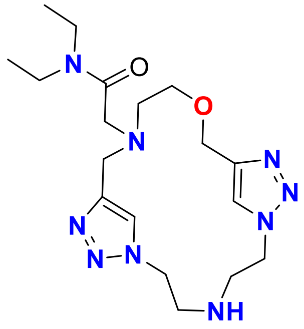

Based on above general procedure,

Diazide **2a*** (186 mg, 1.19 mmol) and di-alkyne (300 mg, 1.19 mmol) afford **MC**. Light brown viscous liquid; yield: 420 mg (86%).

^{1}H-NMR (500 MHz) of **MC4** in $CDCl_3$: δ (ppm) 1.29 (t, 6H), 2.73 (t, 2H), 3.14 (t, 2H), 3.35 (t, 2H), 3.62 (q, 4H), 4.10 (s, 2H), 4.28 (t, 3H), 4.57 (s, 2H), 7.38 (s, 1H), 7.50 (s, 1H). "#" and "*" represent the residual proton of internal standard tetramethyl silane and $CDCl_3$ respectively. ^{13}C NMR (125 MHz, CDCl13) δ (ppm) 14.39, 45.20, 47.37, 49.62, 50.37, 52.53, 52.89, 56.56, 58.04. LC-MS calculated $[M+H]^+$: 406.26 Da, observed $[M+H]^+$: 335.55 Da. HRMS(ESI) m/z calculated for $C_{18}H_{31}N_9O_2$ $[M+H]^+$: 428.2498 Da; found $[M+H]^+$: 428.2501 Da. FT-IR (ATR) υ~ = 3389 cm^{-1} (stretching, N-H), 2937 cm^{-1} (stretching, alkene C-H), 2109 (stretching, N=N-N), 1670 (stretching, C=O) and 1600 cm^{-1} (stretching, C=C)

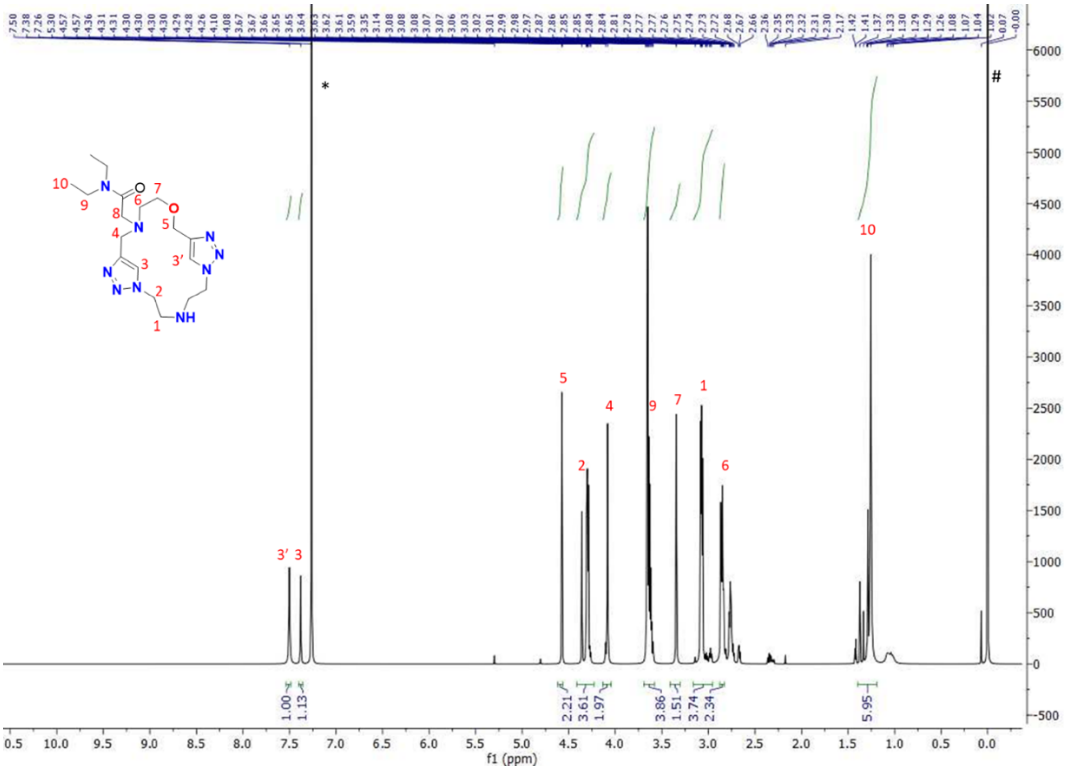

Figure S35. ^{1}H-NMR (500 MHz) of **MC4** in $CDCl_3$.

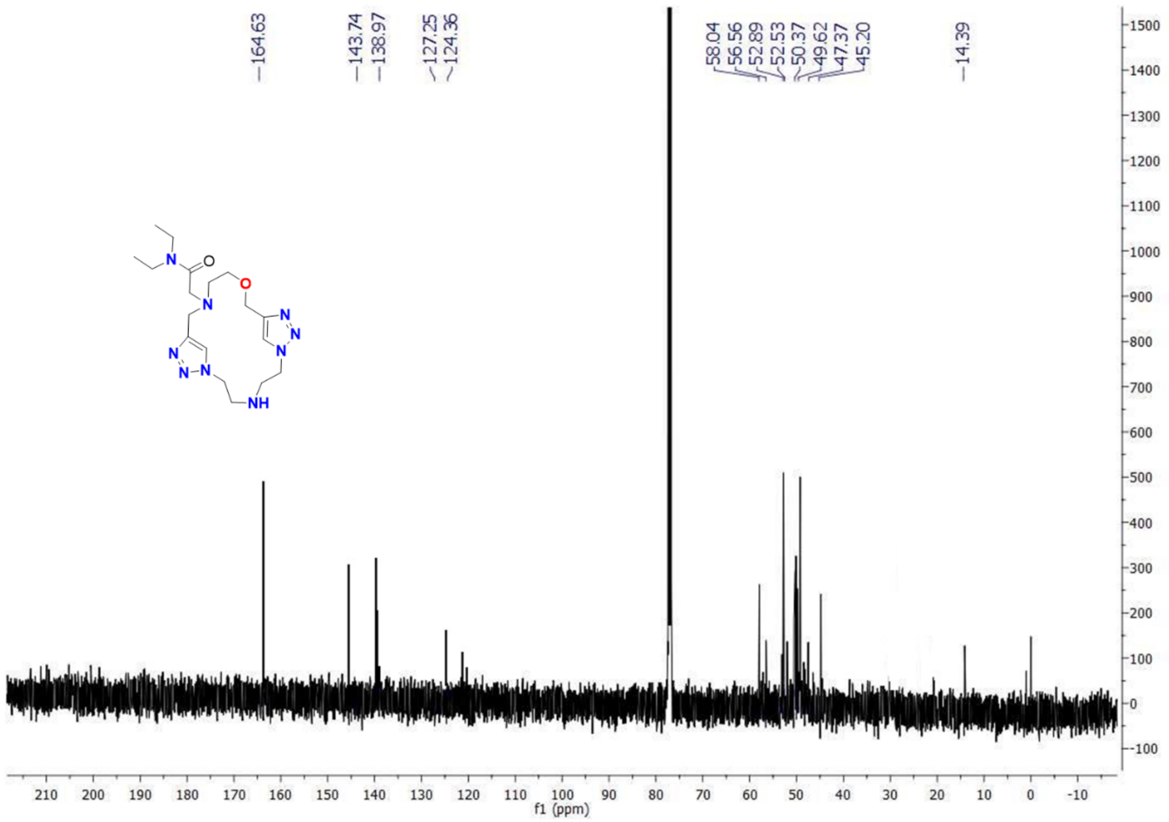

Figure S36. ^{13}C-NMR of **MC4** in $CDCl_3$.

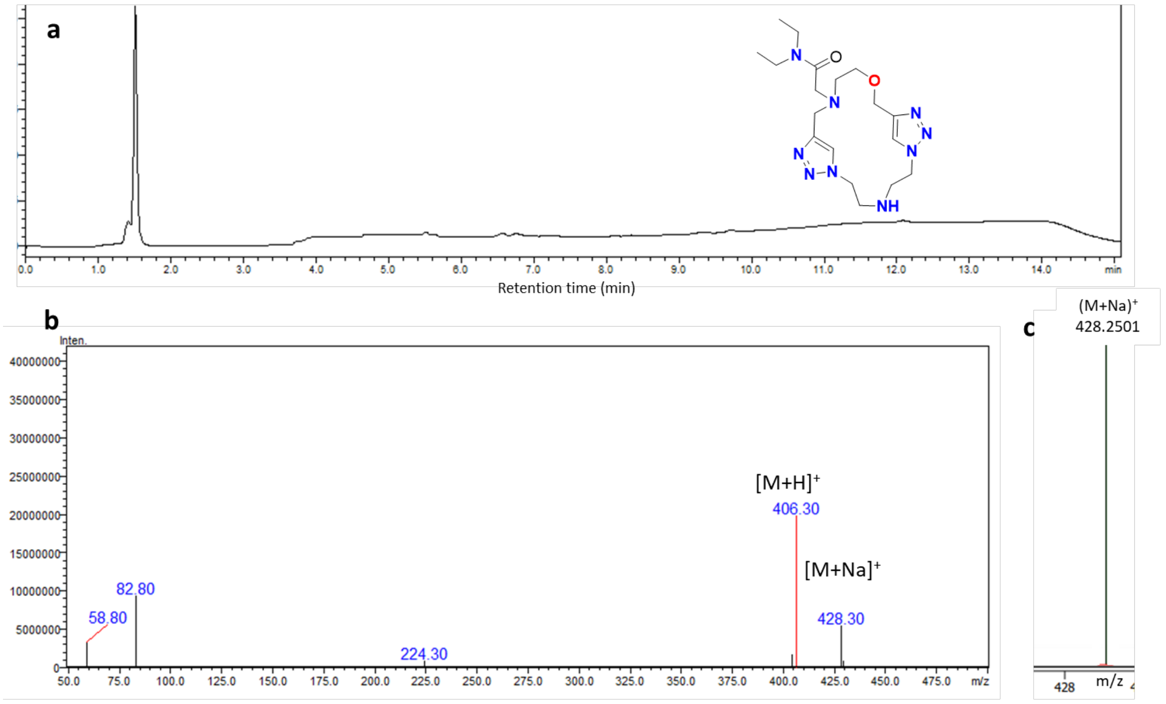

Figure S37. a: HPLC of **MC4** eluted in acetonitrile and water gradient recorded at 210 nm. **b**: Full range MS spectrum in positive ion mode. **c**: HRMS with $[M+H]^+$ peak.

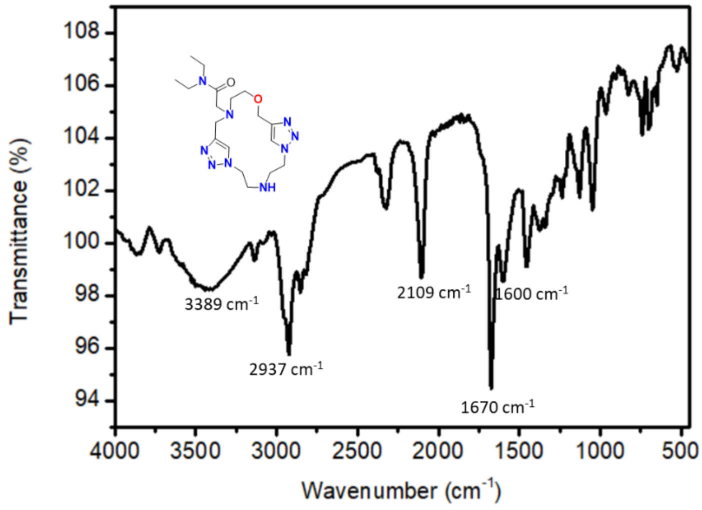

Figure S38. FT-IR spectrum of **MC4**.

Macrocycle 5

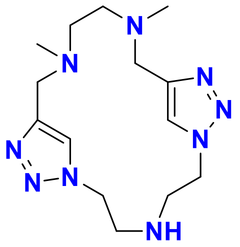

Based on above general procedure,

Diazide **3a*** (189 mg, 1.27 mmol) and di-alkyne (200 mg, 1.27 mmol) afford **MC5**. Dark brown viscous liquid; yield: 350 mg (86%).

^{1}H-NMR (500 MHz) of **MC5** in $CDCl_3$: δ (ppm) 2.6 (s, 6H), 2.60 (s, 4H), 3.38 (t, 3H), 3.74 (s, 4H). 4.45 (t, 4H). "#" and "*" represent the residual proton of internal standard tetramethyl silane and $CDCl_3$ respectively. ^{13}C NMR (125 MHz, CDCl3) δ (ppm) 42.62, 44.10, 48.51,52.52, 54.03, 123.98, 144.49. LC-MS calculated $[M+H]^+$: 320.23 Da, observed $[M+H]^+$: 320.35 Da, $[M+Na]^+$: 342.45. HRMS(ESI) m/z calculated for $C_{14}H_{25}N_9O$ $[M+H]^+$: 320.2311 Da; found $[M+H]^+$: 320.2306 Da. FT-IR (ATR) $\tilde{\upsilon}$ = 3333 cm^{-1} (stretching, N-H), 2979 cm^{-1} (stretching, alkene C-H), 2112 (stretching, N=N-N) and 1642 cm^{-1} (stretching, C=C)

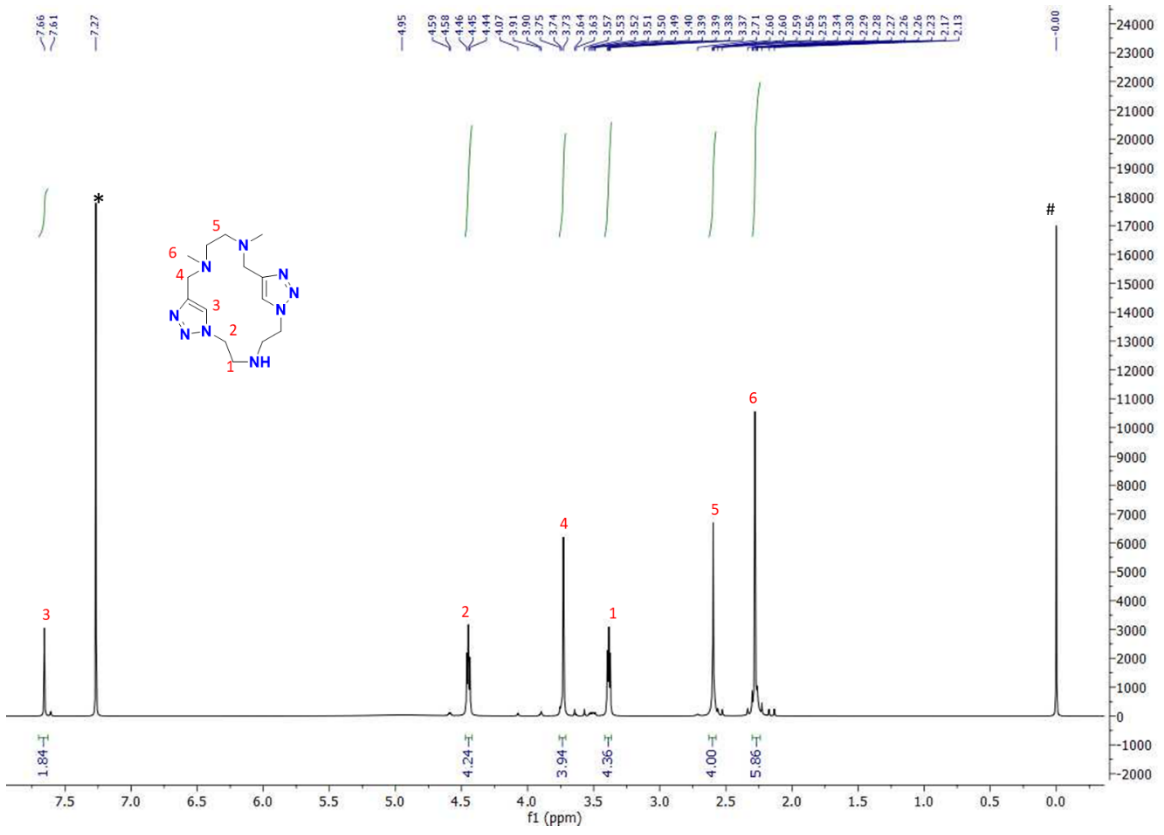

Figure S39. ^{1}H-NMR (500 MHz) of **MC5** in $CDCl_3$.

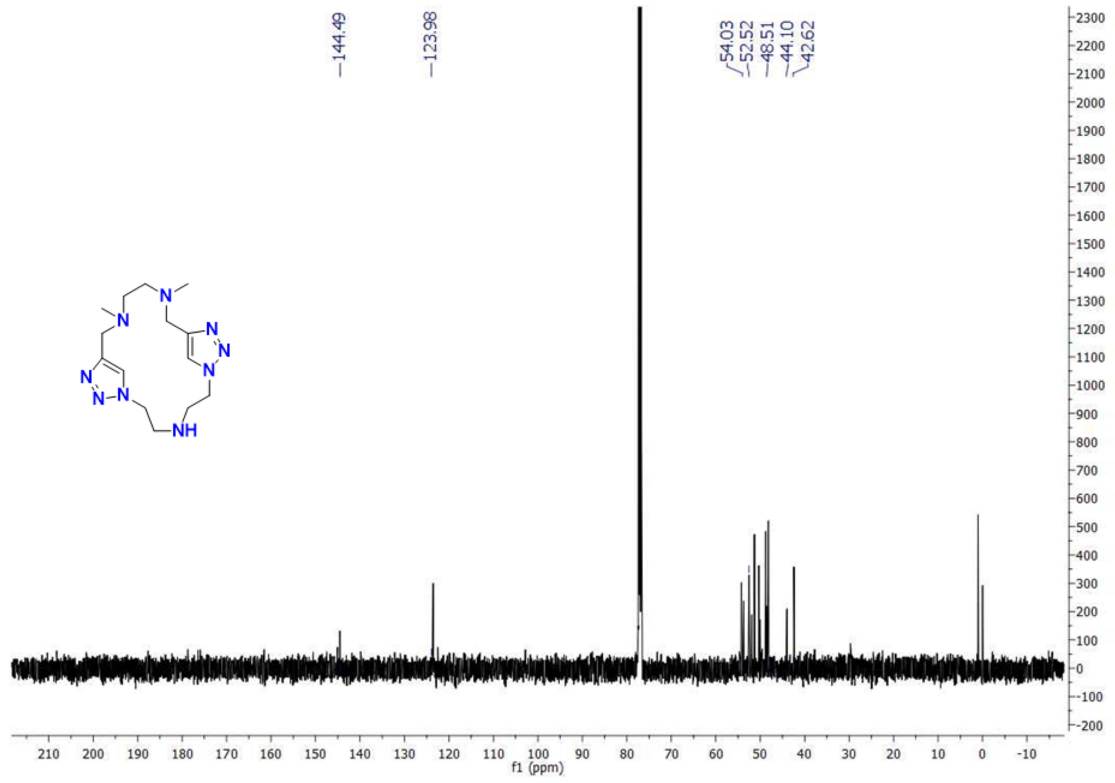

Figure S40. ^{13}C-NMR of **MC5** in $CDCl_3$.

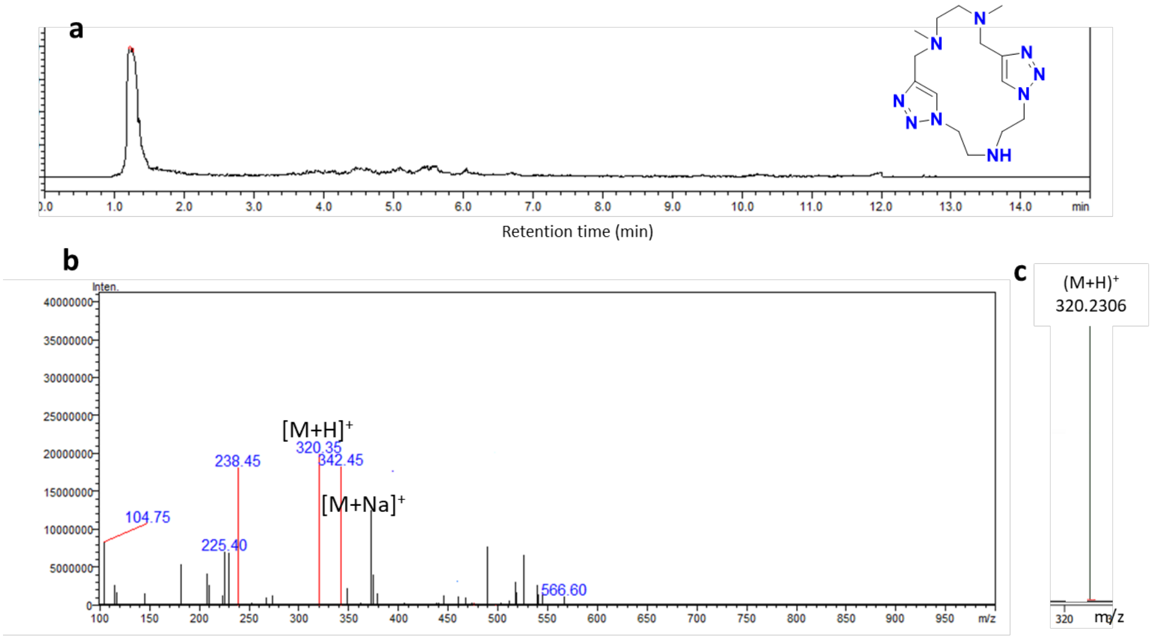

Figure S41. a: HPLC of **MC5** eluted in acetonitrile and water gradient recorded at 210 nm. **b**: Full range MS spectrum in positive ion mode. **c**: HRMS with $[M+H]^+$ peak.

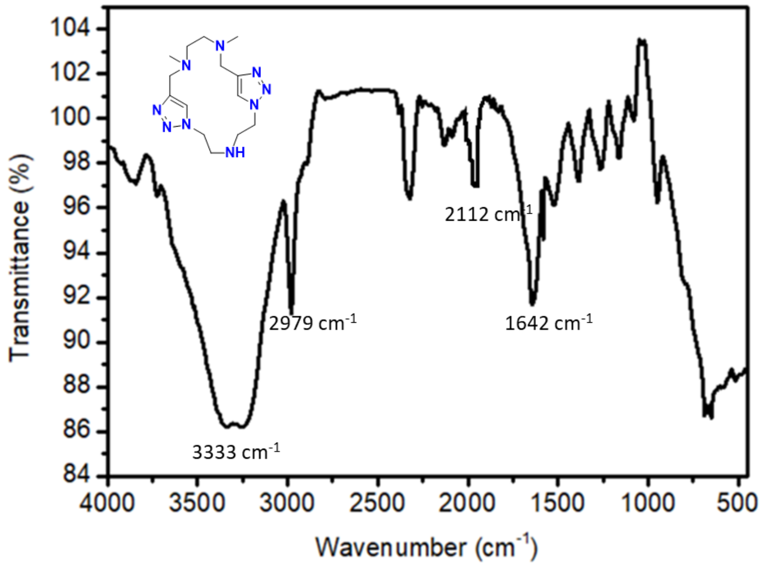

Figure S42. FT-IR spectrum of **MC5**.

Macrocycle 6

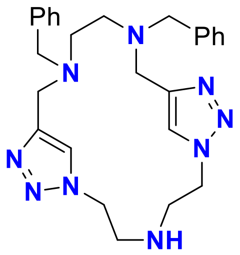

Based on above general procedure,

Diazide **3b*** (245 mg, 1.58 mmol) and di-alkyne (500 mg, 1.58 mmol) afford **MC6**. Dark brown viscous liquid; yield: 625 mg (83%).

^{1}H-NMR (500 MHz) of **MC6** in $CDCl_3$: δ (ppm) 2.53 (s, 4H), 2.75 (t, 4H), 3.75 (s, 4H), 3.79 (s, 4H), 3.99 (t, 4H), 7.34 (m, 10H), 7.54 (s, 2H). ^{13}C NMR (125 MHz, CDC13) δ (ppm) 44.48, 46.61, 49.21, 50.99, 59.86, 124.74, 129.10, 129.49, 137.93, 143.01, 146.65. LC-MS calculated $[M+H]^+$: 472.29 Da, observed $[M+H]^+$: 472.20 Da, $[M+Na]^+$: 494.15 Da. HRMS(ESI) m/z calculated for $C_{26}H_{33}N_9$ $[M+H]^+$: 472.2938 Da; found $[M+H]^+$: 472.3018 Da. FT-IR (ATR) $\upsilon^{\sim}$ = 3333 cm^{-1} (stretching, N-H), 2922 cm^{-1} (stretching, alkene C-H), 2112 (stretching, N=N-N) and 1669 cm^{-1} (stretching, C=C)

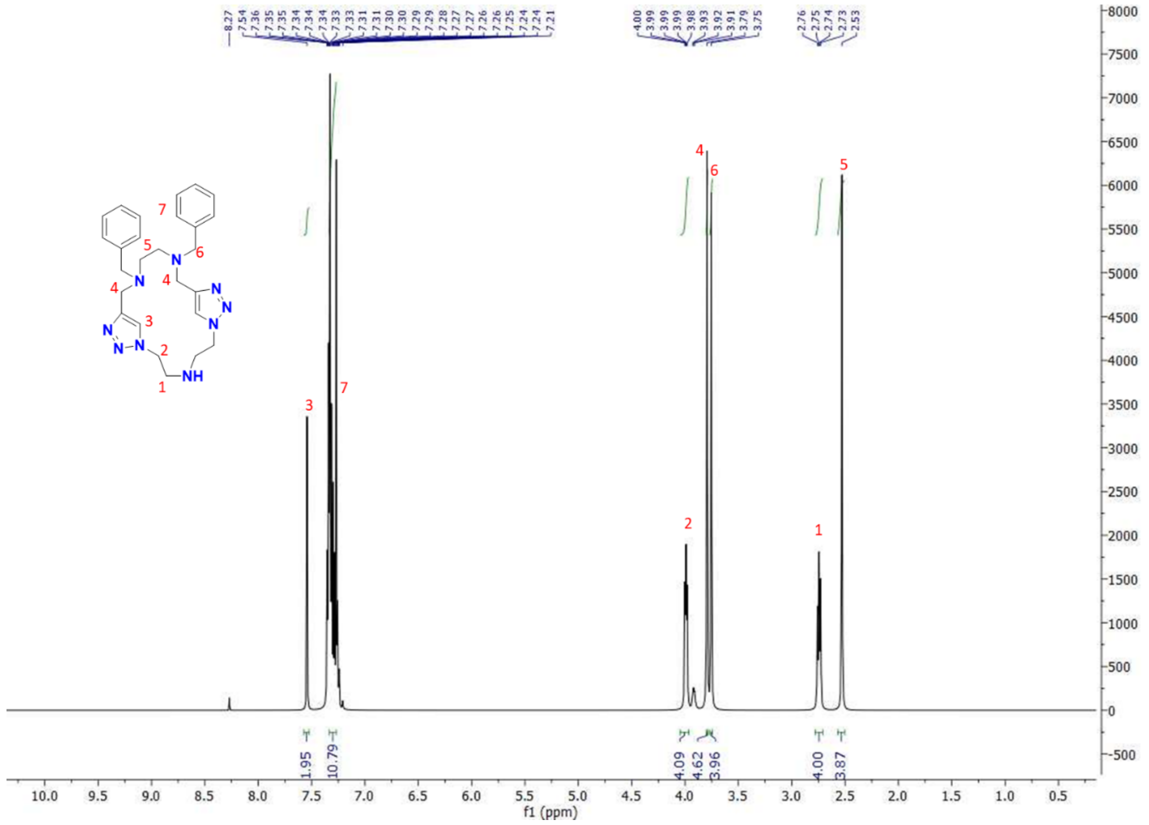

Figure S43. ^{1}H-NMR (500 MHz) of **MC6** in $CDCl_3$.

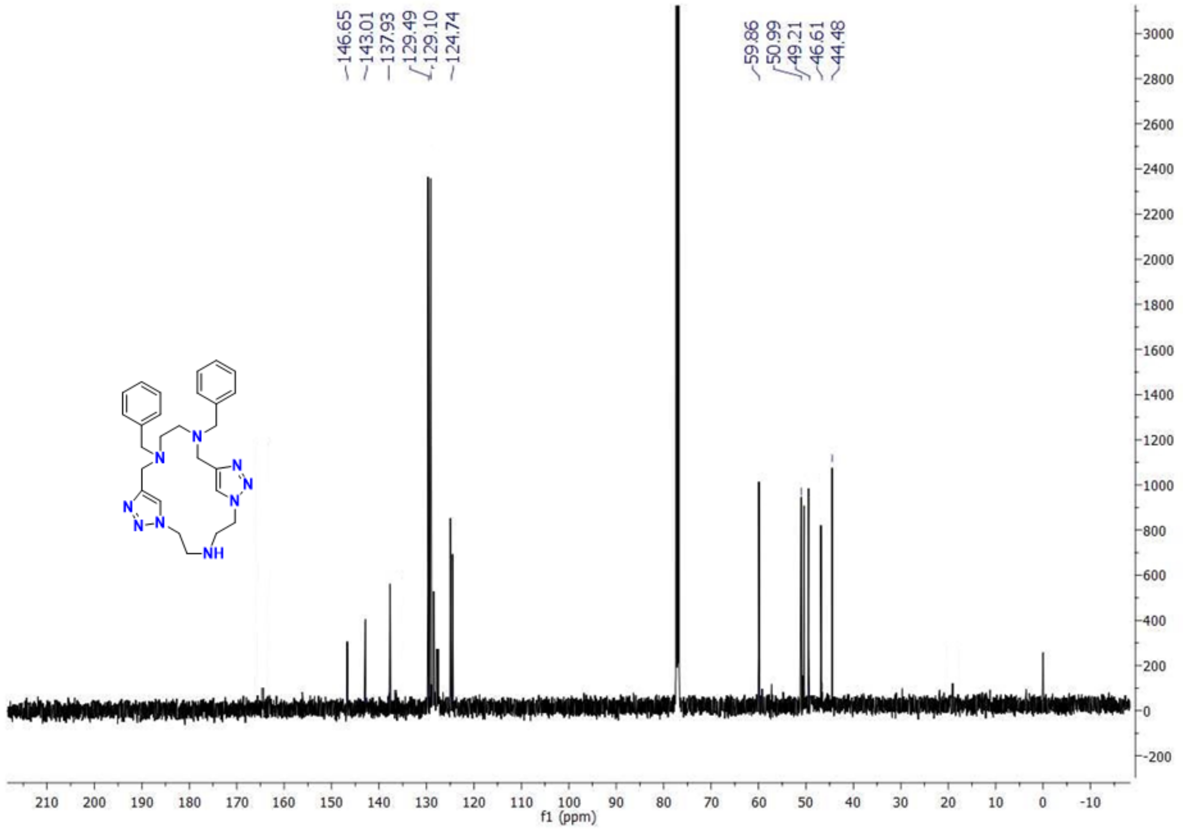

Figure S44. ^{13}C-NMR of **MC6** in $CDCl_3$.

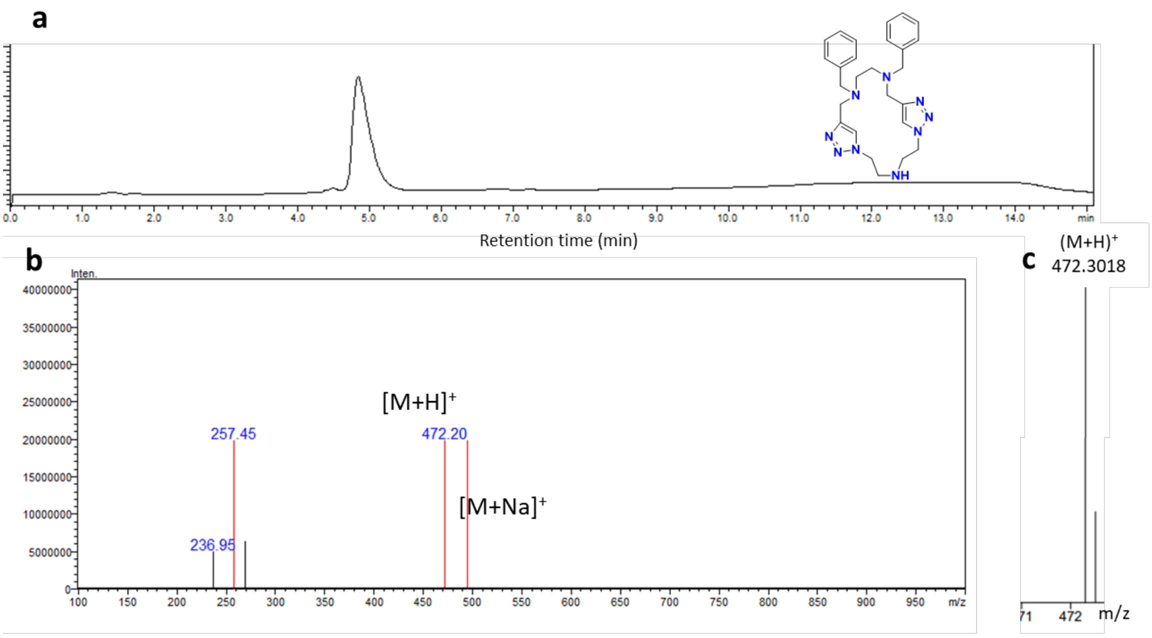

Figure S45. a: HPLC of **MC6** eluted in acetonitrile and water gradient recorded at 254 nm. **b**: Full range MS spectrum in positive ion mode. **c**: HRMS with $[M+H]^+$ peak.

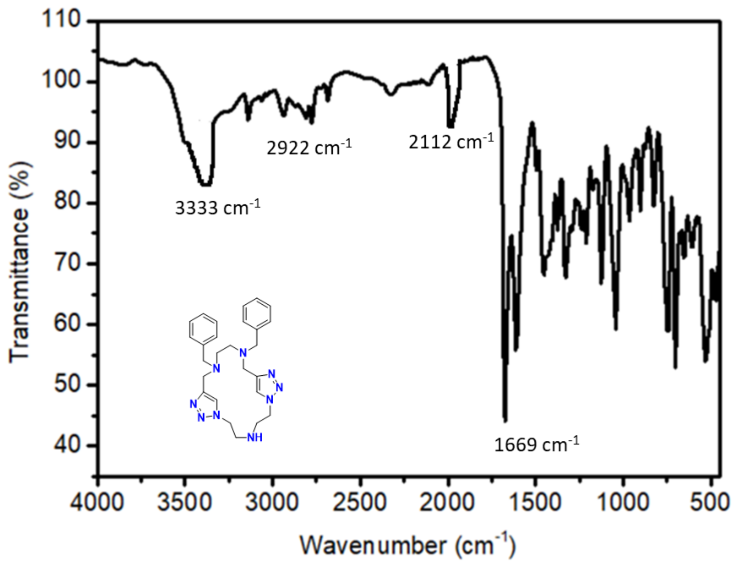

Figure S46. FT-IR spectrum of **MC6**.

Macrocycle 7

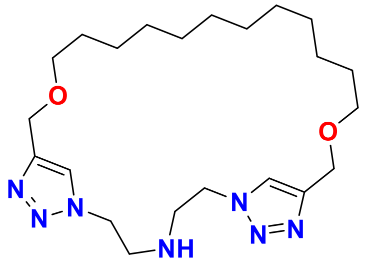

Based on above general procedure,

Diazide **5a*** (445 mg, 2.87 mmol) and di-alkyne (800 mg, 2.87 mmol) afford **MC7**. Pale yellow viscous liquid; yield: 950 mg (76%).

^{1}H-NMR (500 MHz) of **MC7** in $CDCl_3$: δ (ppm) 0.95 (m, 12H), 1.38 (m,4H), 1.648 (m, 4H), 2.97 (t, 4H), 3.43 (t, 4H), 3.68 (t, 4H), 4.69 (s, 4H), 7.54 (s, 4H). "*" represents the residual proton of $CDCl_3$. ^{13}C NMR (125 MHz, CDCl3) δ (ppm) 26.12, 26.48, 27.97, 28.36, 29.10, 29.51, 49.63, 58.40, 64.97, 70.50, 122.85, 148.89. LC-MS calculated $[M+H]^+$: 434.32 Da, observed $[M+H]^+$: 434.30 Da, $[M+Na]^+$: 456.45. HRMS(ESI) m/z calculated for $C_{22}H_{39}N_7O_2$ $[M+H]^+$: 434.3243 Da; found $[M+H]^+$: 434.3232 Da. FT-IR (ATR) $\tilde{\upsilon}$ = 3345 cm^{-1} (stretching, N-H), 2915 cm^{-1} (stretching, alkene C-H), 2095 (stretching, N=N-N) and 1660 cm^{-1} (stretching, C=C)

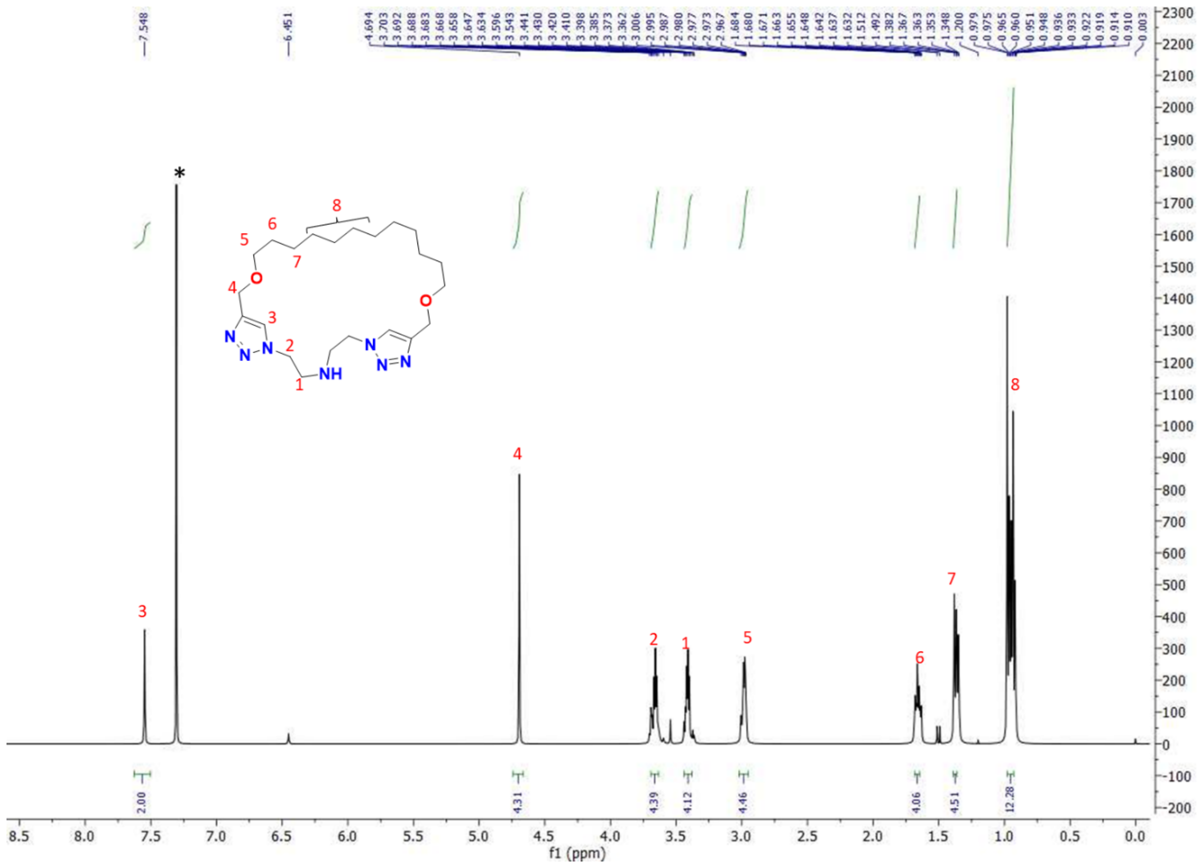

Figure S47. ^{1}H-NMR (500 MHz) of **MC7** in $CDCl_3$.

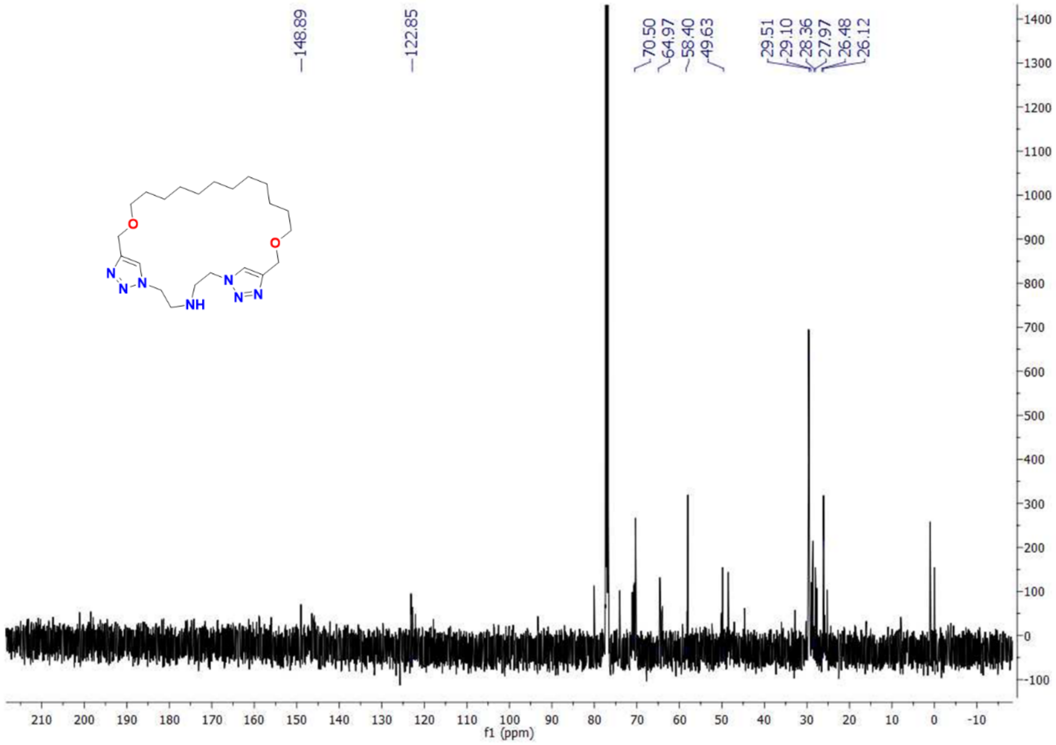

Figure S48. ^{13}C-NMR of **MC8** in $CDCl_3$.

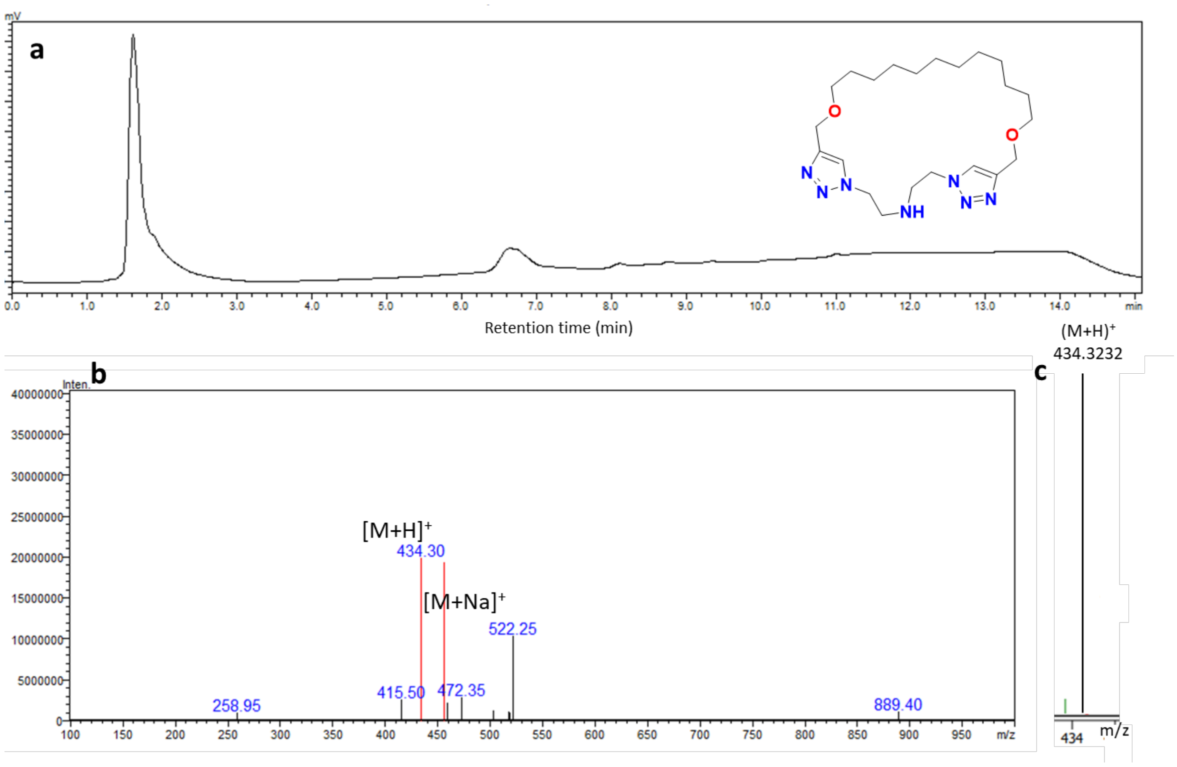

Figure S49. a: HPLC of **MC7** eluted in acetonitrile and water gradient recorded at 210 nm. **b**: Full range MS spectrum in positive ion mode. **c**: HRMS with $[M+H]^+$ peak.

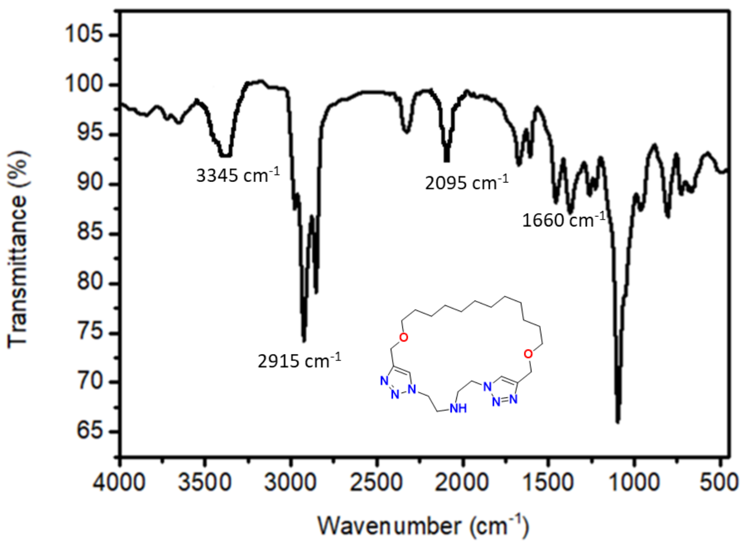

Figure S50. FT-IR spectrum of **MC7**.

Macrocycle 8

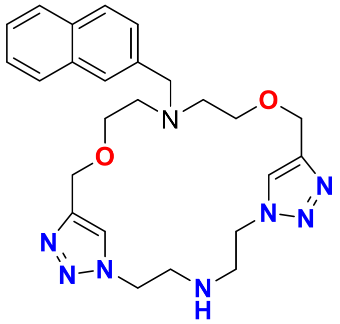

Based on above general procedure,

Diazide **3a*** (240 mg, 1.55 mmol) and di-alkyne (500 mg, 1.55 mmol) afford **MC8**. Light brown viscous liquid; yield: 570 mg (77%).

^{1}H-NMR (500 MHz) of **MC8** in $CDCl_3$: δ (ppm) 2.97 (t, 4H), 3.10 (t, 4H), 3.64 (t, 4H), 3.69 (t, 4H), 4.13 (s, 2H), 4.30 (t, 4H), 4.58 (s, 4H), 7.30 (s, 2H), 7.37-7.84 (m, 7H). "#" and "*" represent the residual proton of internal standard tetramethyl silane and $CDCl_3$ respectively. ^{13}C NMR (125 MHz, CDC13) δ (ppm) 48.87, 50.72, 54.76, 58.79, 64.61, 69.38, 123.63, 124.73, 125.48, 125.77, 127.27, 127.64, 128.37,132.39, 132.78, 133.83, 134.97, 145.61. LC-MS calculated $[M+H]^+$: 477.27 Da, observed $[M+H]^+$: 477.25 Da, $[M+Na]^+$: 499.35. HRMS(ESI) m/z calculated for $C_{25}H_{32}N_8O_2$ $[M+H]^+$: 477.2757 Da; found $[M+H]^+$: 477.2793 Da. FT-IR (ATR) $\upsilon^{\sim}$ = 3368 cm^{-1} (stretching, N-H), 2944 cm^{-1} (stretching, alkene C-H), 2109 (stretching, N=N-N) and 1657 cm^{-1} (stretching, C=C)

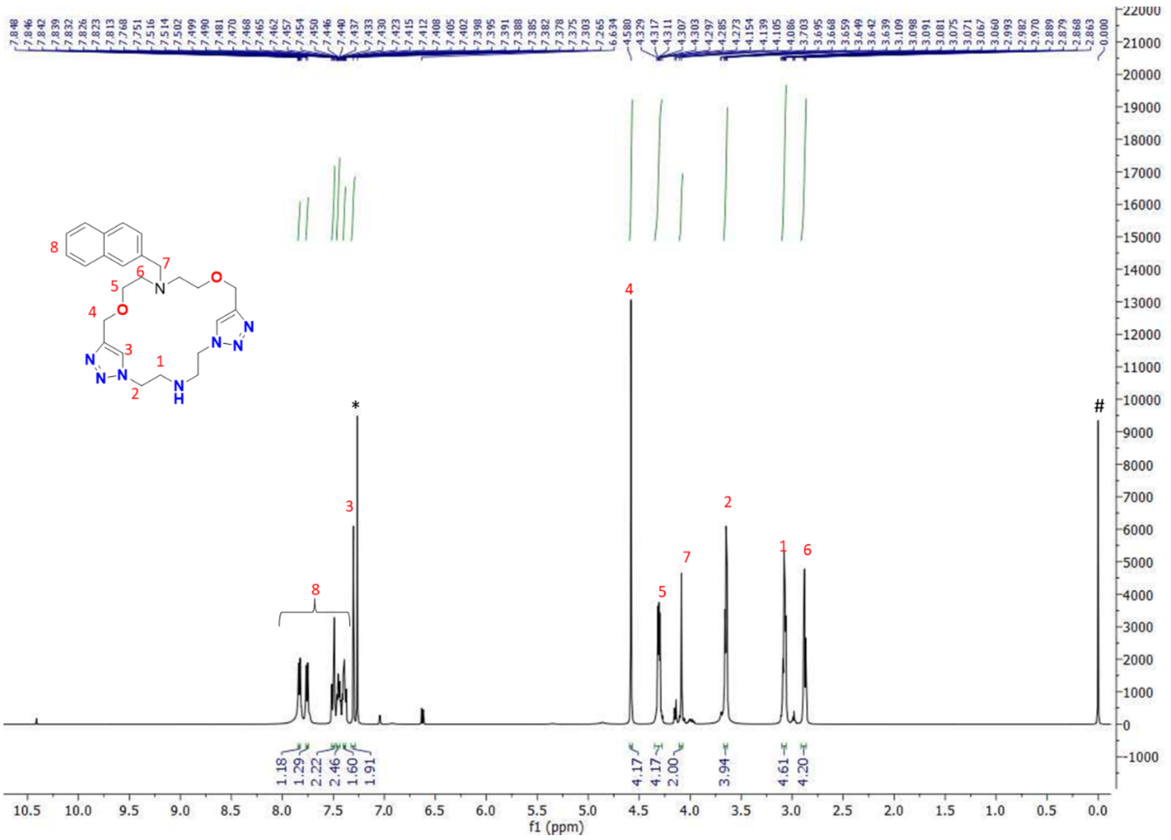

Figure S51. ^{1}H-NMR (500 MHz) of **MC8** in $CDCl_3$.

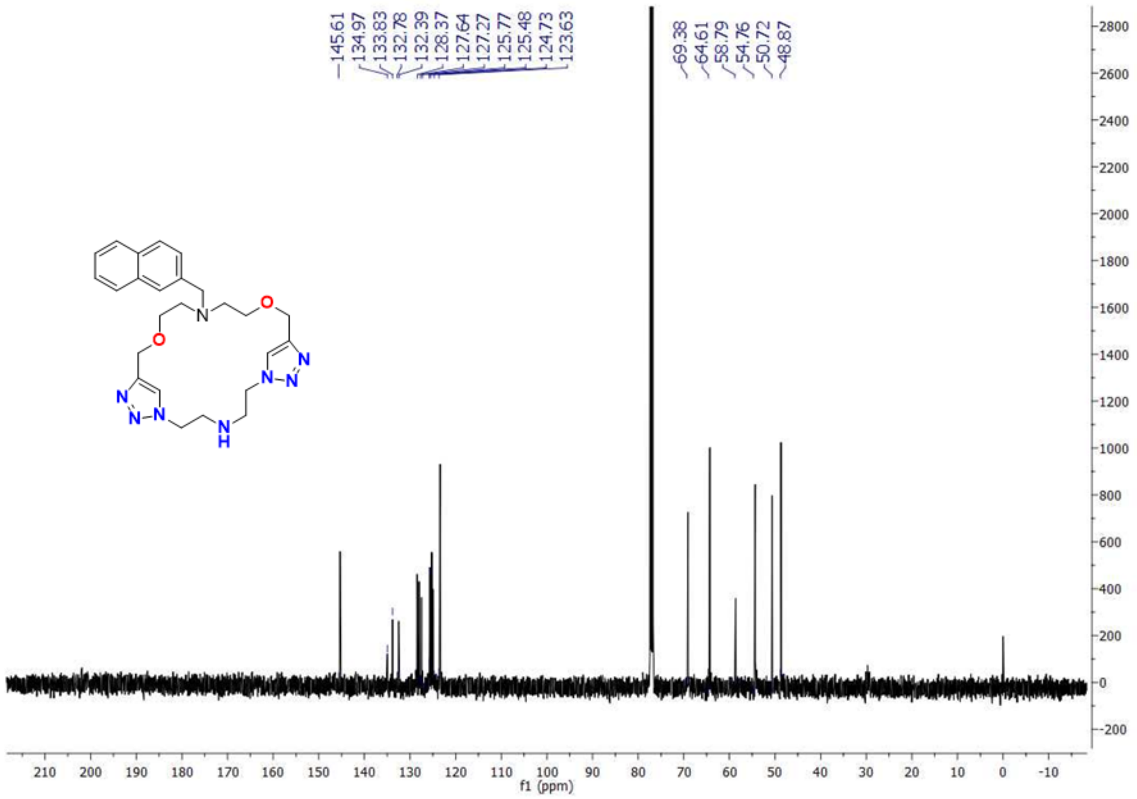

Figure S52. ^{13}C-NMR of **MC8** in $CDCl_3$.

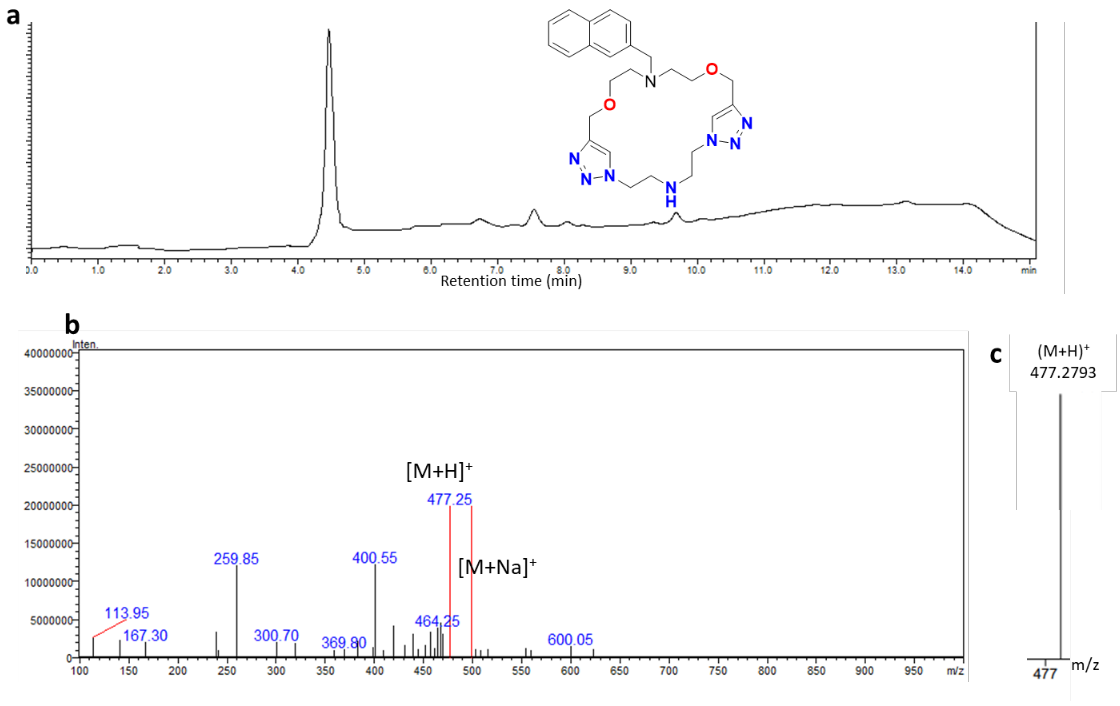

Figure S53. a: HPLC of **MC8** eluted in acetonitrile and water gradient recorded at 254 nm. **b**: Full range MS spectrum in positive ion mode. **c**: HRMS with $[M+H]^+$ peak.

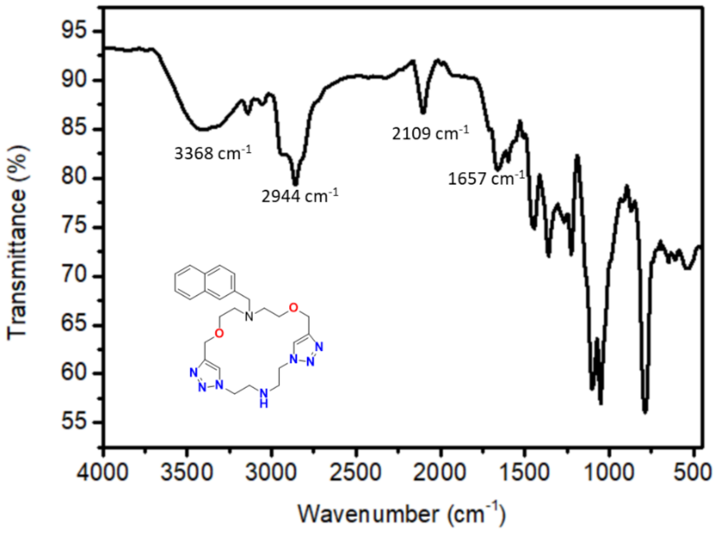

Figure S54. FT-IR spectrum of **MC8**.

8. Bioactive properties of the macrocycles

8.1 Physicochemical properties

The druggability of the synthesized macrocycles are tested by calculating the physicochemical properties by using the webtool SWISSADME [32] the properties are compared with the conventional guidelines for drug.

Table S1. Physicochemical properties of the macrocycles: MW – Molecular weight, logP – lipophilicity, PSA – Polar surface area, HBD – Hydrogen bond donors, HBA – Hydrogen bond acceptors, RB – Rotatable bonds.

Property	Lipinski's Guidelines	MC1	MC2	MC3	MC4	MC5	MC6	MC7	MC8
**MW**	**≤500**	334.42	334.42	374.48	405.5	319.41	471.60	433.59	476.57
**logP**	**≤5**	-0.18	-0.24	0.47	-0.85	-0.88	1.52	2.25	1.26
**PSA (A^o)**	**≤140**	85.92	85.92	85.92	106.23	79.93	79.93	91.91	95.15
**No. of HBD**	**≤5**	1	1	1	1	1	1	1	1
**No. of HBA**	**≤10**	7	7	7	8	7	7	7	8

8.2 Fluorescence spectroscopy for protein interaction studies

8.2.1 Experimental Procedure

10 μM BSA was prepared by dissolving 6.6 mg of BSA in 10 mL of phosphate buffer of pH 7.4. The macrocycles were dissolved in DMSO to make the stock solution of 2 mM concentration. 2 mL of the BSA control solution was taken in the fluorescence cuvette to take the emission at zero concentration of the compound. The titration was carried out by varying the concentration of macrocycle from 2 μM to 100 μM. An equilibration time of 3 minutes is given for each measurement after the addition of the solution. Binding constant were estimated from the Stern-Volmer plot by plotting I_o/I vs concentration of the macrocycle. The slope of the graph was attributed as the binding constant.

8.2.2 Fluorescence spectra for the interaction of BSA and macrocycles

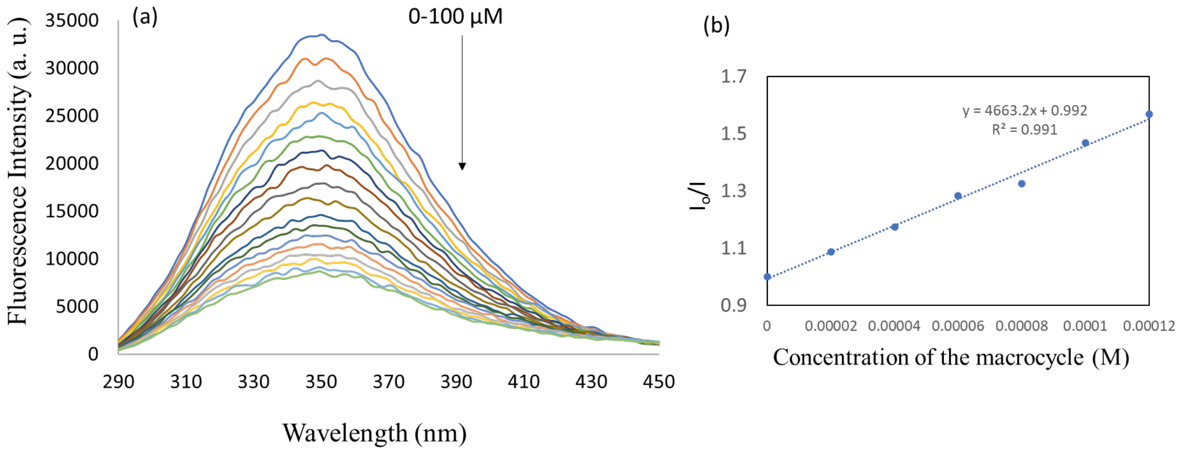

Figure S55. a. Fluorescence spectrum of macrocycle **MC1** on interaction with BSA. **b.** The plot of I_o/I vs concentration of macrocycle. The slope of the graph is attributed as binding constant, which is 4.6×10^3 M^{-1}.

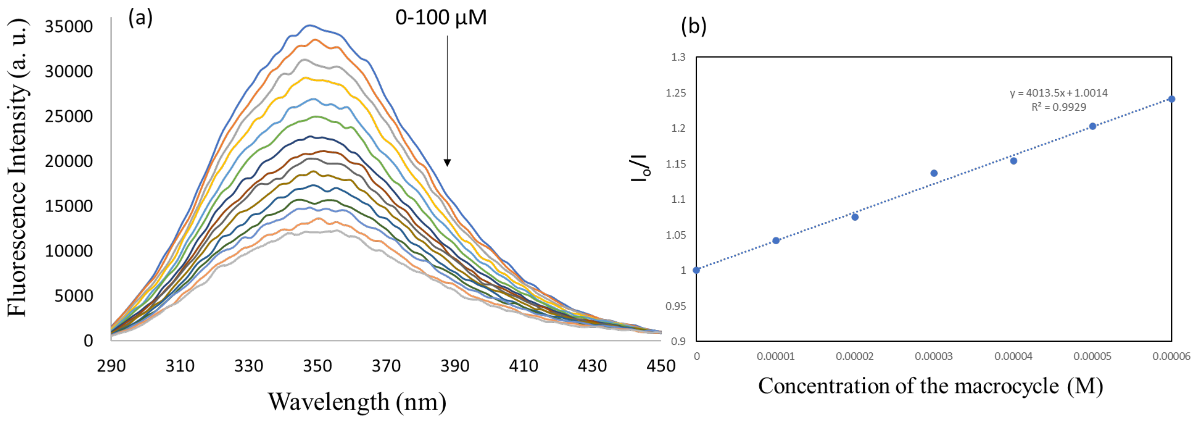

Figure S56. a. Fluorescence spectrum of macrocycle **MC2** on interaction with BSA. **b.** The plot of I_o/I vs concentration of macrocycle. The slope of the graph is attributed as binding constant, which is 4.0×10^3 M^{-1}.

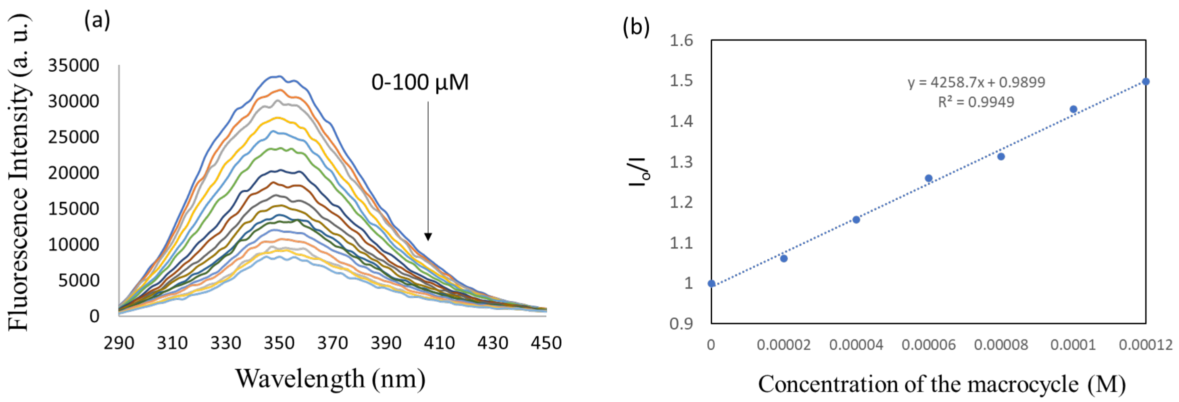

Figure S57. a. Fluorescence spectrum of macrocycle **MC3** on interaction with BSA. **b.** The plot of I_o/I vs concentration of macrocycle. The slope of the graph is attributed as binding constant, which is 4.2×10^3 M^{-1}.

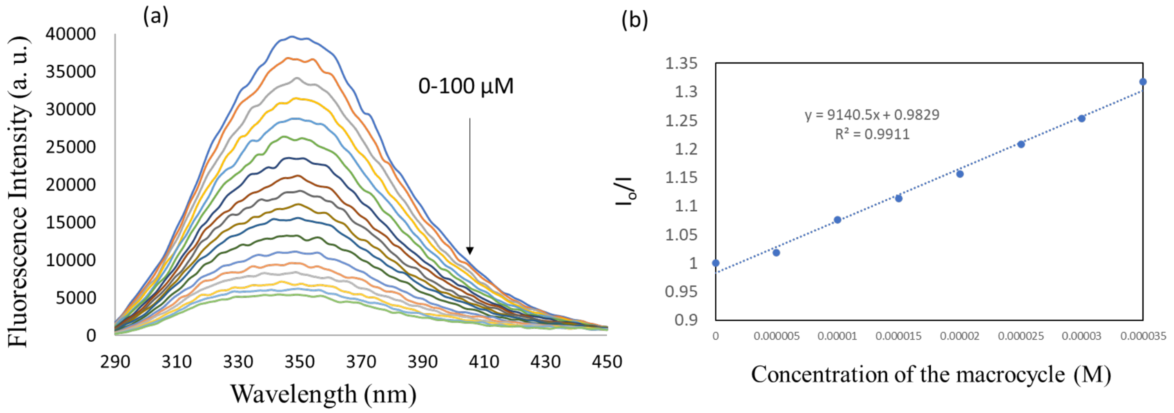

Figure S58. a. Fluorescence spectrum of macrocycle **MC4** on interaction with BSA. **b.** The plot of I_o/I vs concentration of macrocycle. The slope of the graph is attributed as binding constant, which is 9.1×10^3 M^{-1}.

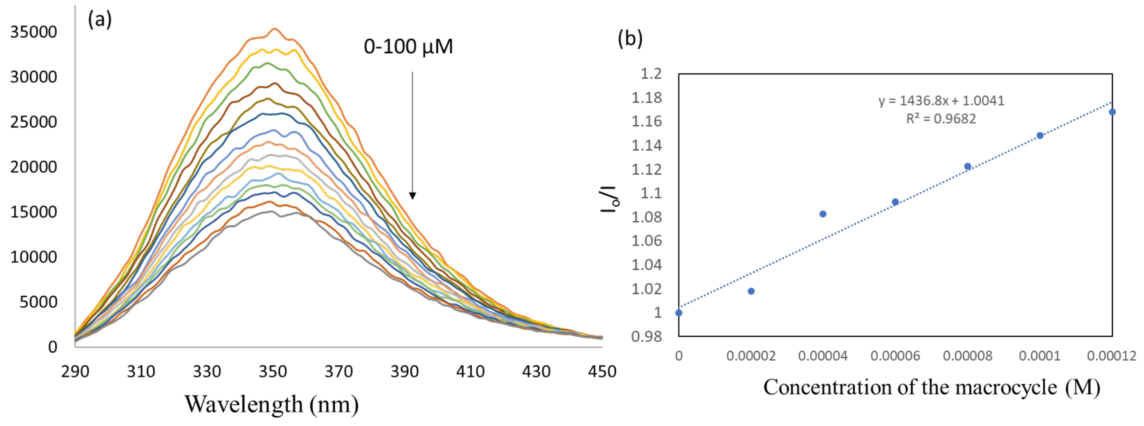

Figure S59. a. Fluorescence spectrum of macrocycle **MC5** on interaction with BSA. **b.** The plot of I_o/I vs concentration of macrocycle. The slope of the graph is attributed as binding constant, which is 1.4×10^3 M^{-1}.

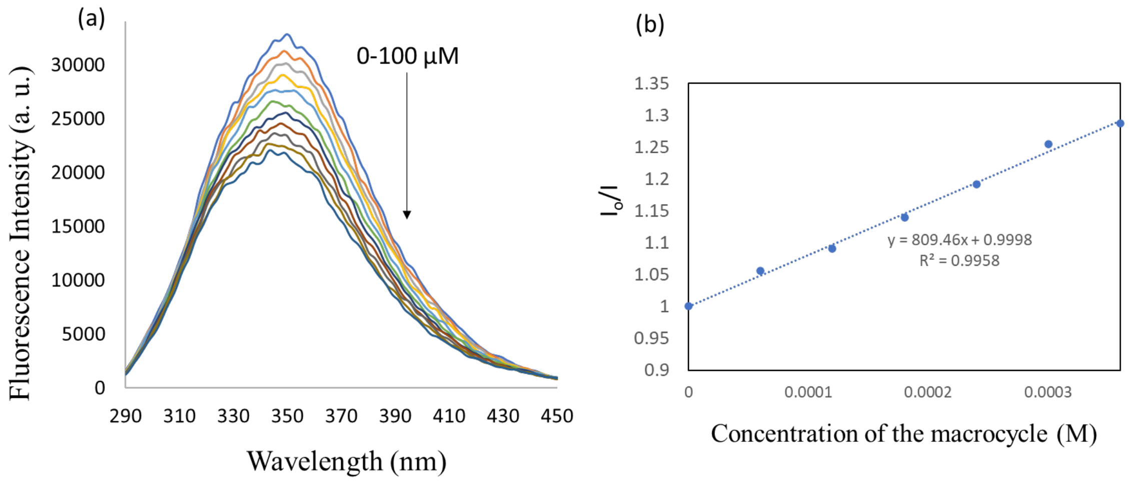

Figure S60. a. Fluorescence spectrum of macrocycle **MC6** on interaction with BSA. **b.** The plot of I_o/I vs concentration of macrocycle. The slope of the graph is attributed as binding constant, which is 0.8×10^3 M^{-1}.

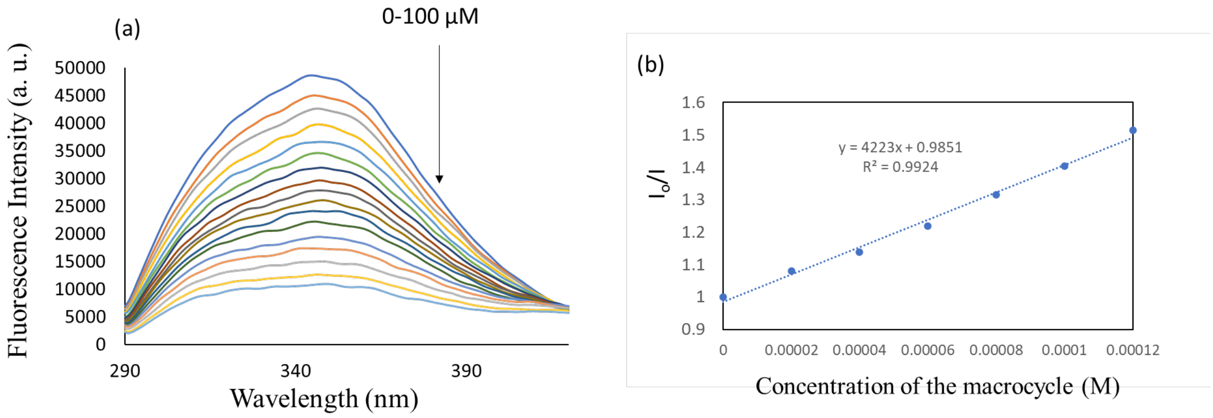

Figure S61. a. Fluorescence spectrum of macrocycle **MC1** on interaction with HSA. **b.** The plot of I_o/I vs concentration of macrocycle. The slope of the graph is attributed as binding constant, which is 4.2×10^3 M^{-1}.

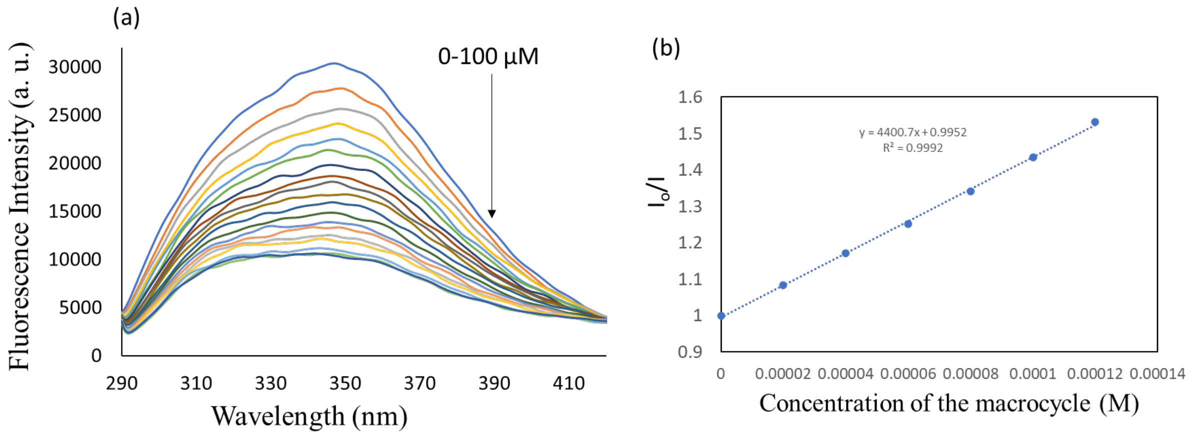

Figure S62. a. Fluorescence spectrum of macrocycle **MC2** on interaction with HSA. **b.** The plot of I_o/I vs concentration of macrocycle. The slope of the graph is attributed as binding constant, which is 4.4×10^3 M^{-1}.

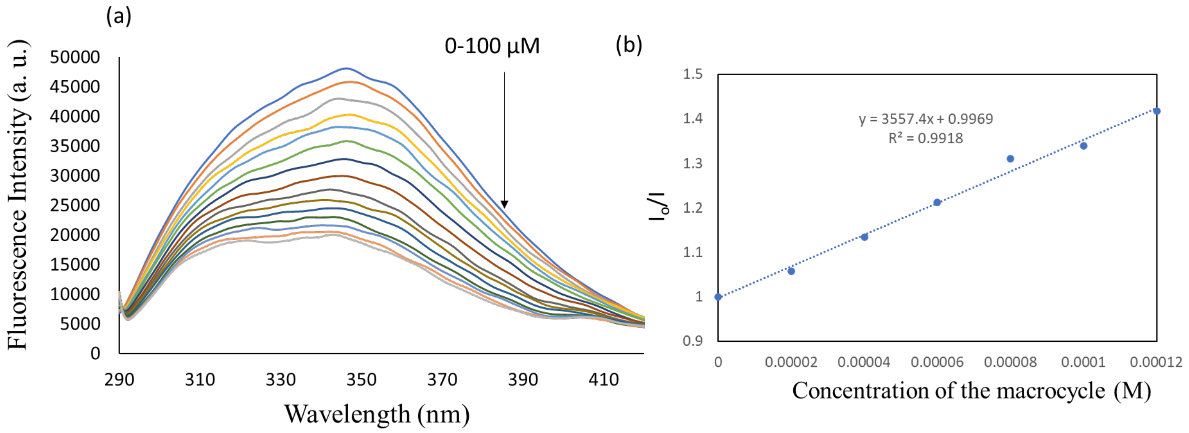

Figure S63. a. Fluorescence spectrum of macrocycle **MC3** on interaction with HSA. **b.** The plot of I_o/I vs concentration of macrocycle. The slope of the graph is attributed as binding constant, which is 3.5×10^3 M^{-1}.

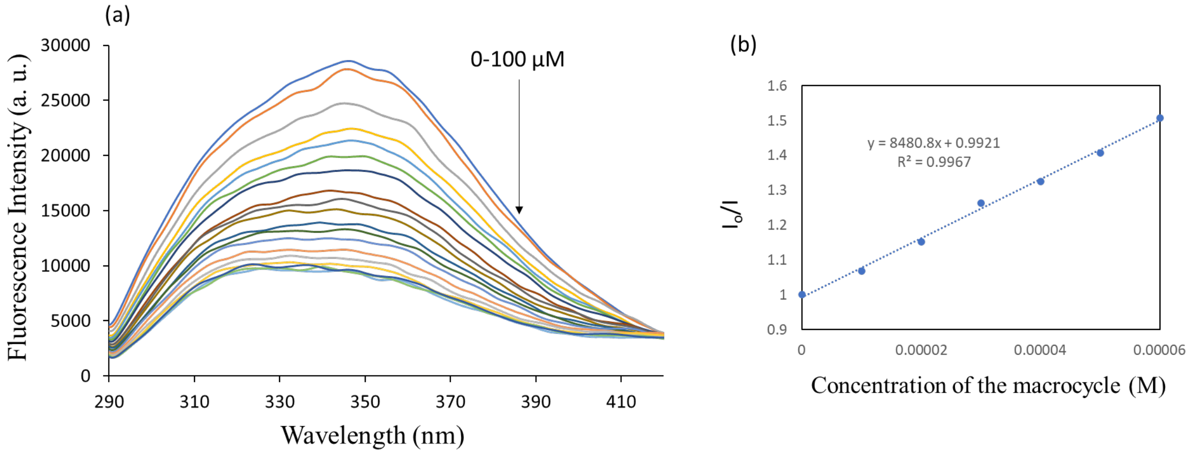

Figure S64. a. Fluorescence spectrum of macrocycle **MC4** on interaction with HSA. **b.** The plot of I_o/I vs concentration of macrocycle. The slope of the graph is attributed as binding constant, which is 8.4×10^3 M^{-1}.

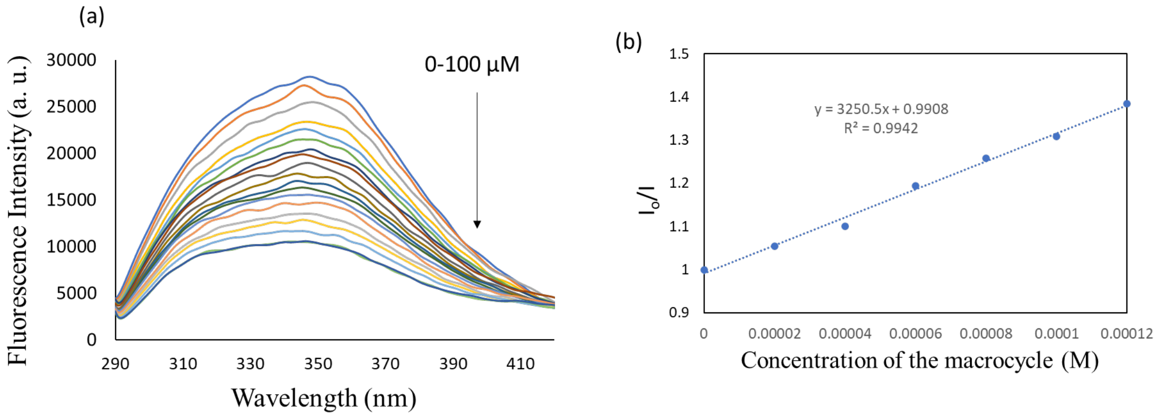

Figure S65. a. Fluorescence spectrum of macrocycle **MC5** on interaction with HSA. **b.** The plot of I_o/I vs concentration of macrocycle. The slope of the graph is attributed as binding constant, which is 3.2×10^3 M^{-1}.

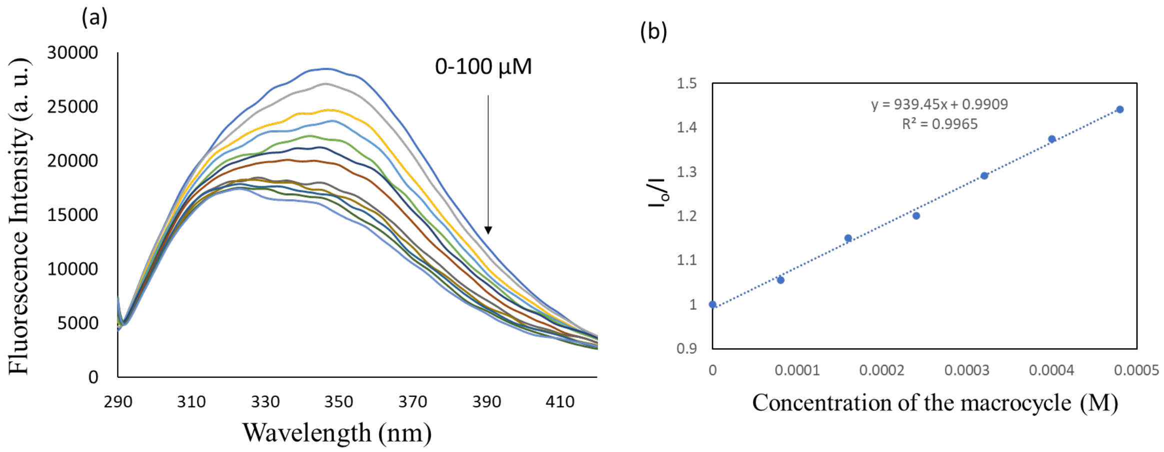

Figure S66. a. Fluorescence spectrum of macrocycle **MC6** on interaction with HSA. **b.** The plot of I_o/I vs concentration of macrocycle. The slope of the graph is attributed as binding constant, which is 0.92×10^3 M^{-1}.

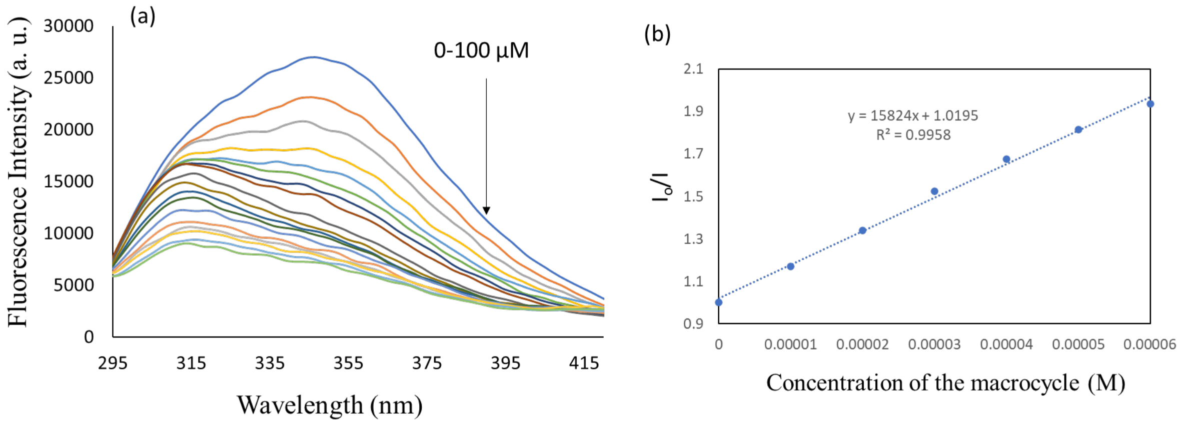

Figure S67. a. Fluorescence spectrum of macrocycle **MC7** on interaction with HSA. **b.** The plot of I_o/I vs concentration of macrocycle. The slope of the graph is attributed as binding constant, which is 15.8×10^3 M^{-1}.

8.3. NMR studies for protein-macrocycle interaction

A comparative study of the ^1H NMR spectra of BSA (**Figure S68 a**), BSA mixed with **MC2 (Figure S68 b**) and the spectrum of **MC2** (**Figure S68 c**) was implemented. BSA resulted in spectrum with massive broadened peaks mostly in the region of 0.5 to 3.75 ppm due to high number of protons in it. The spectrum of BSA with **MC2** showed significant changes from the BSA spectrum due to the association of **MC2** with it as expected from a literature (*P. Singh, H. Singh, V. Castro-Aceituno, S. Ahn, Y. Kim, D. C. Yang, RSC Adv.,* ***2017****, 7, 15397-15407*) Correlating the peaks of **b** and **c,** we could observe a shift in the proton signals of the macrocycle (**Figure S68**). A set of protons of **MC2** in the range of 1.5 to 5 ppm showed an upfield shift and the protons in the aromatic region showed a downfield shift. As the BSA peaks are interfering with the peaks at the range of 1 – 4 ppm, we have concentrated on the shift of aromatic protons. As shown in the figure, the two peaks at 7.75 and 7.77 showed a significant change in the chemical shift, which is attributed to the change in the chemical environment of the macrocycle in presence of protein. Thus, this result thus validates the effective binding of macrocycle with the BSA protein.

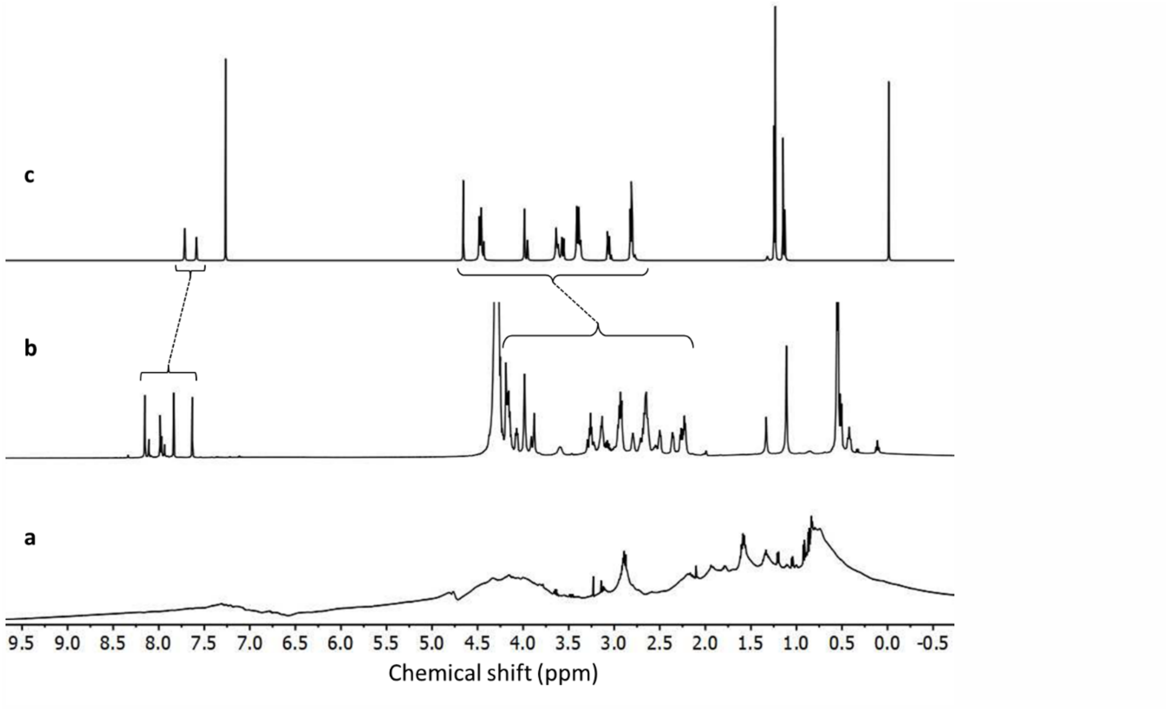

Figure S68. a. ^{1}H-NMR of BSA **b.** ^{1}H-NMR of **MC2** mixed with BSA **c.** ^{1}H-NMR of **MC2**.

8.4. Molecular docking studies

8.4.1 Methodology

Autodock Vina[40] was used to perform the molecular docking studies with Perl Script for integration executables. The pdb structure of the proteins BSA (4F5V, Chain A) and HSA (1BM0, Chain A) were retrieved from the protein data bank (http://www.rcsb.org). Autodock tools 4 [41]was used to add hydrom atom and to compute gasteiger charges. The grid used for the docking were of the size (60x 60x 60) and centred at (x52.09, y22.81, z66.1) for BSA and size (60x 60x 60) and centred at (x29.60, y31.78, z23.48) for HSA. The ligands used were energy minimized using Argus lab 4.0.1 [42], the docked structures were visualized in Pymol and the amino acid residue interaction were studied by using Discovery studio [43].

8.4.2 Determination of binding energy and docked conformation from molecular docking

Table S2. Binding energy of the synthesized macrocycles on interaction with BSA and HSA.

Macrocycle	Binding energy (kcal/mol)	
	BSA	HSA
**MC1**	-7.3	-7.9

**MC2**	-7.5	-7.3
**MC3**	-8.7	-8.3
**MC4**	-8.7	-8.7
**MC5**	-7.7	-7.5
**MC6**	-9.3	-9.1
**MC7**	-9.6	-10.8

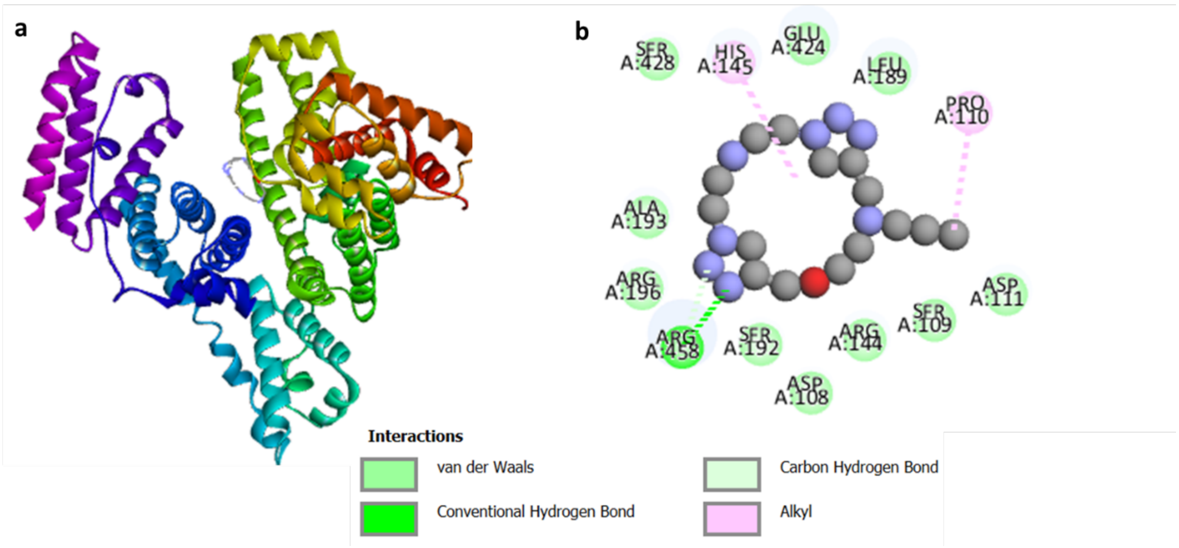

Figure S69. a. The best docked conformation of the **MC1** with the BSA. **b.** 2D diagram of aminoacid interaction of BSA complexed with **MC1**.

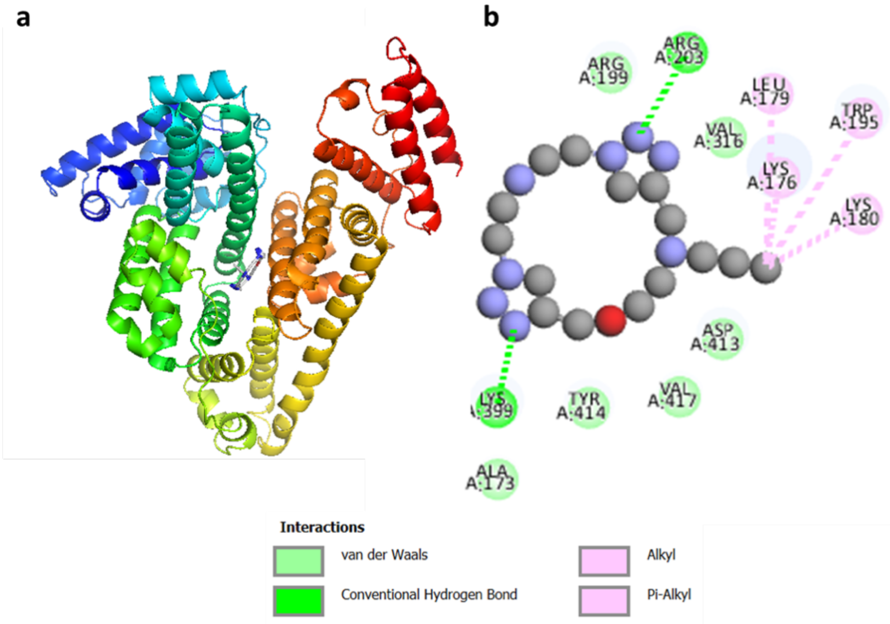

Figure S70. a. The best docked conformation of the **MC1** with the HSA. **b.** 2D diagram of aminoacid interaction of HSA complexed with **MC1**.

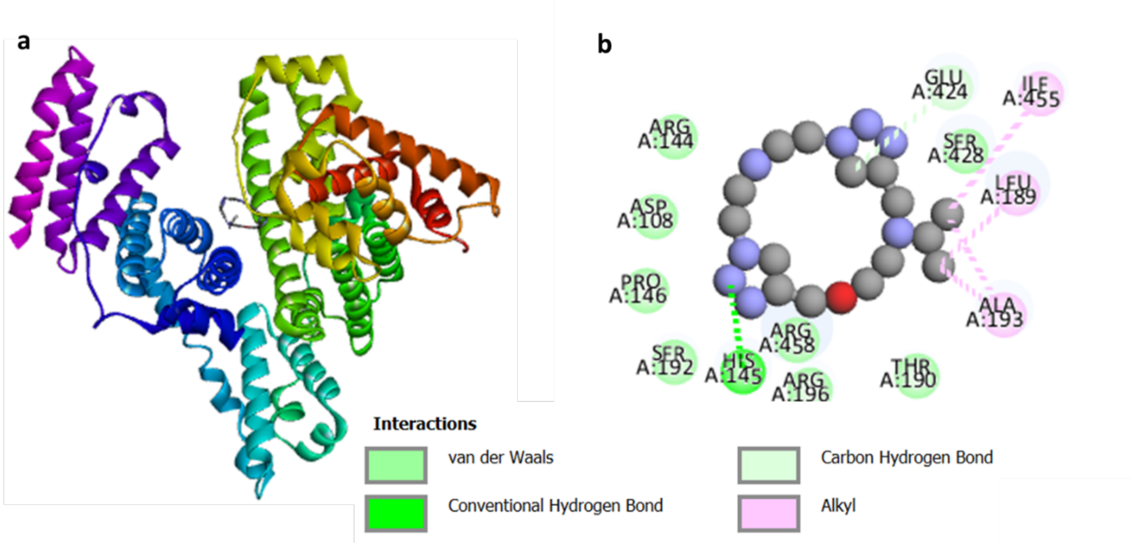

Figure S71. a. The best docked conformation of the **MC2** with the BSA. **b.** 2D diagram of aminoacid interaction of BSA complexed with **MC2**.

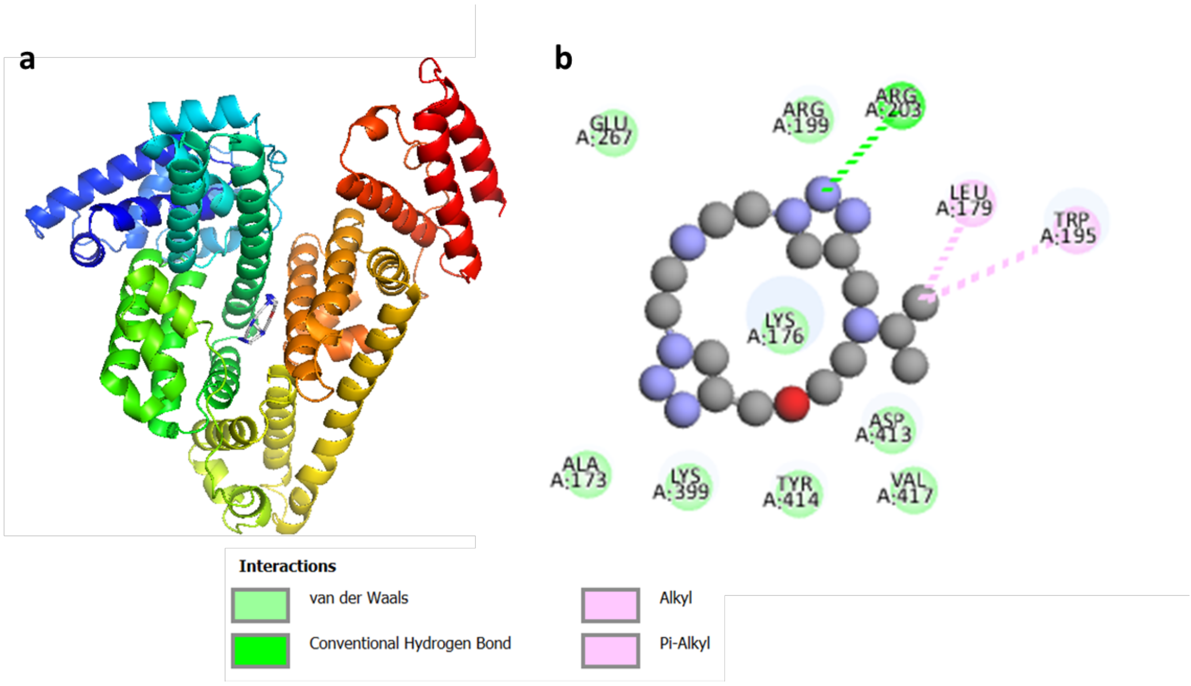

Figure S72. a. The best docked conformation of the **MC2** with the HSA. **b.** 2D diagram of aminoacid interaction of HSA complexed with **MC2**.

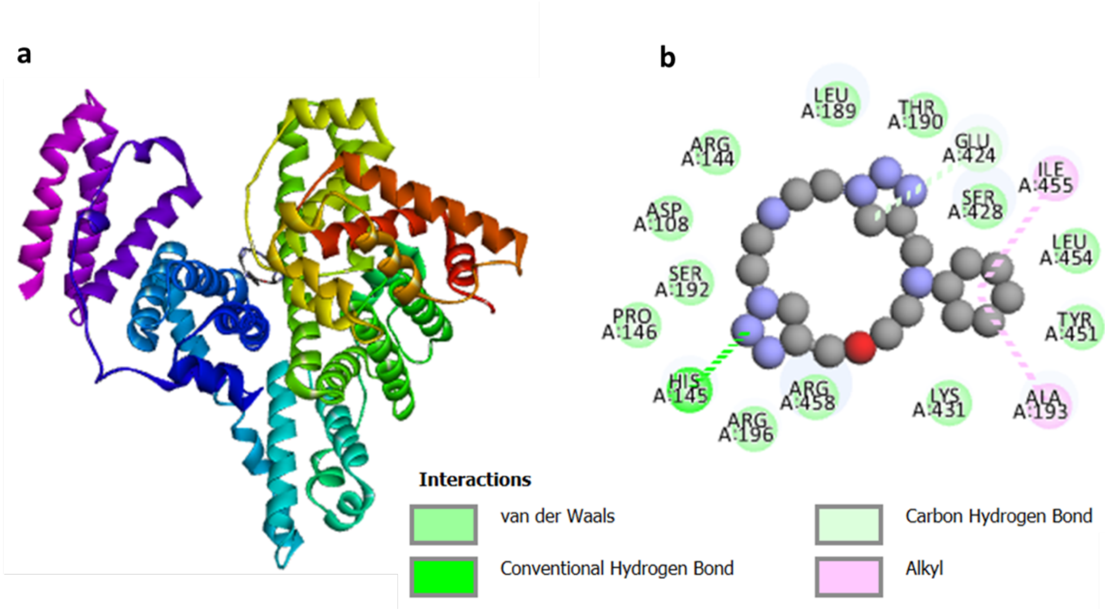

Figure S73. a. The best docked conformation of the **MC3** with the BSA. **b.** 2D diagram of aminoacid interaction of BSA complexed with **MC3**.

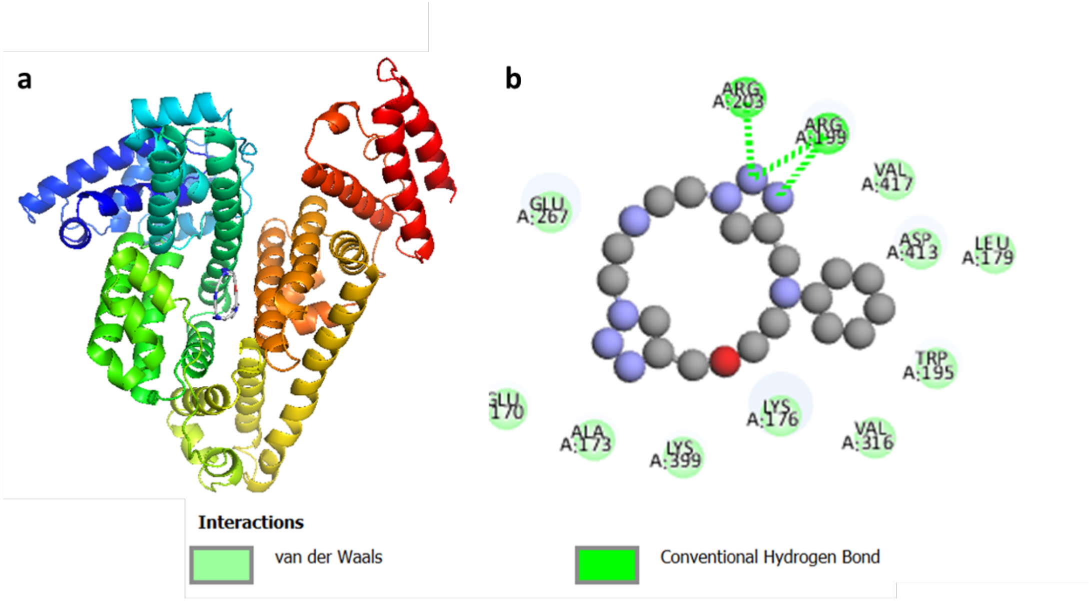

Figure S74. a. The best docked conformation of the **MC3** with the HSA. **b.** 2D diagram of aminoacid interaction of HSA complexed with **MC3**.

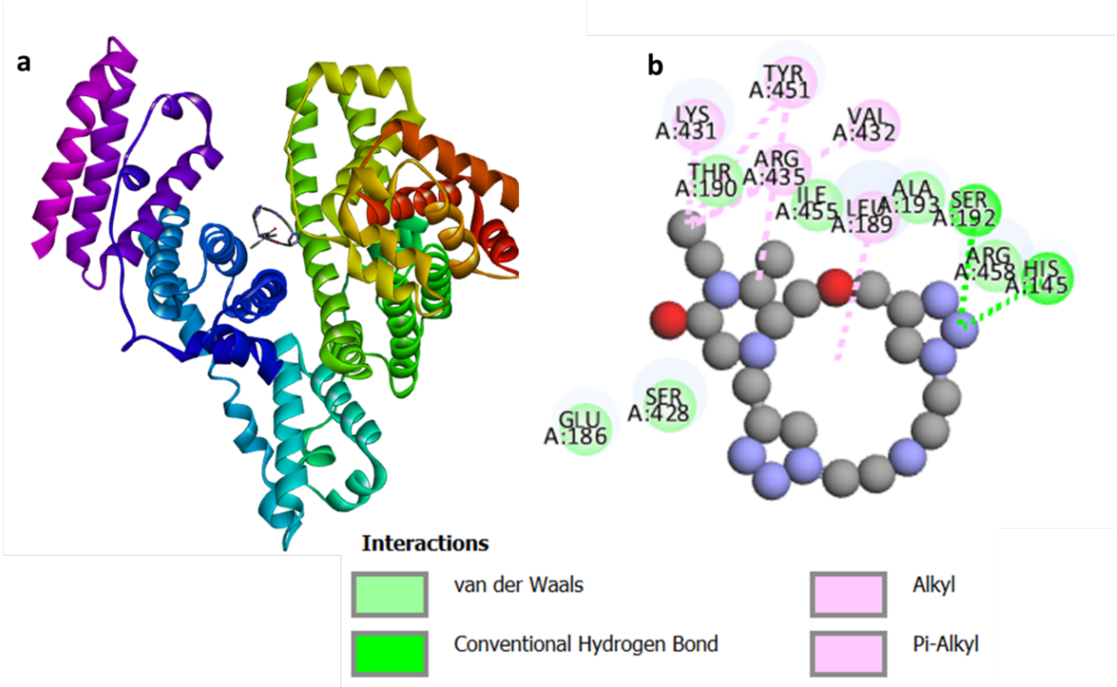

Figure S75. a. The best docked conformation of the **MC4** with the BSA. **b.** 2D diagram of aminoacid interaction of BSA complexed with **MC4**.

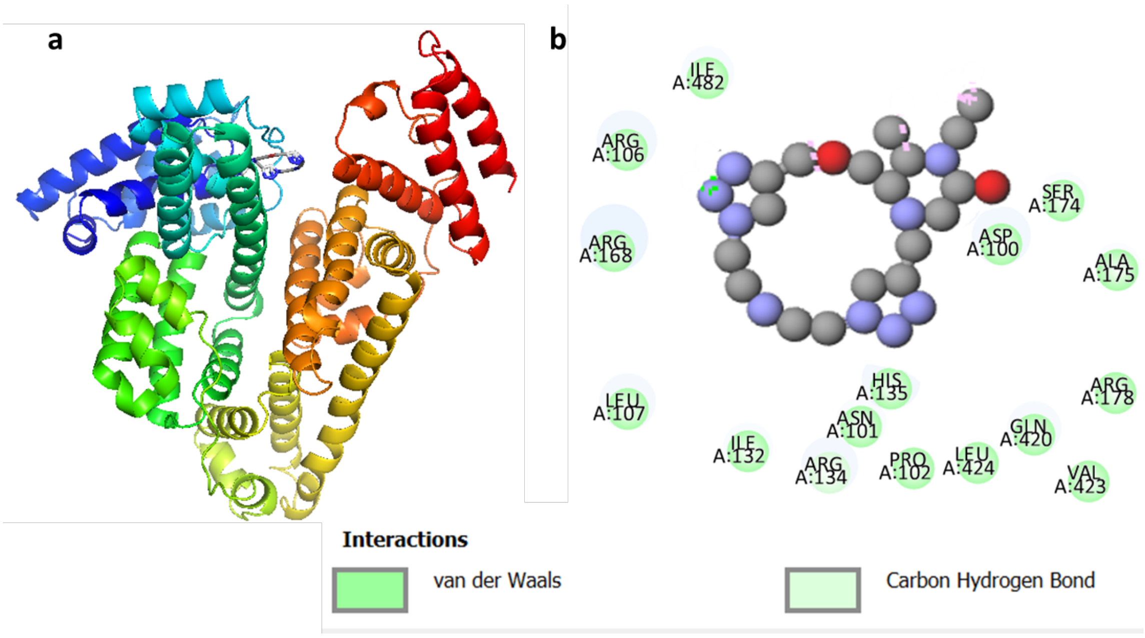

Figure S76. a. The best docked conformation of the **MC4** with the HSA. **b.** 2D diagram of aminoacid interaction of HSA complexed with **MC4**.

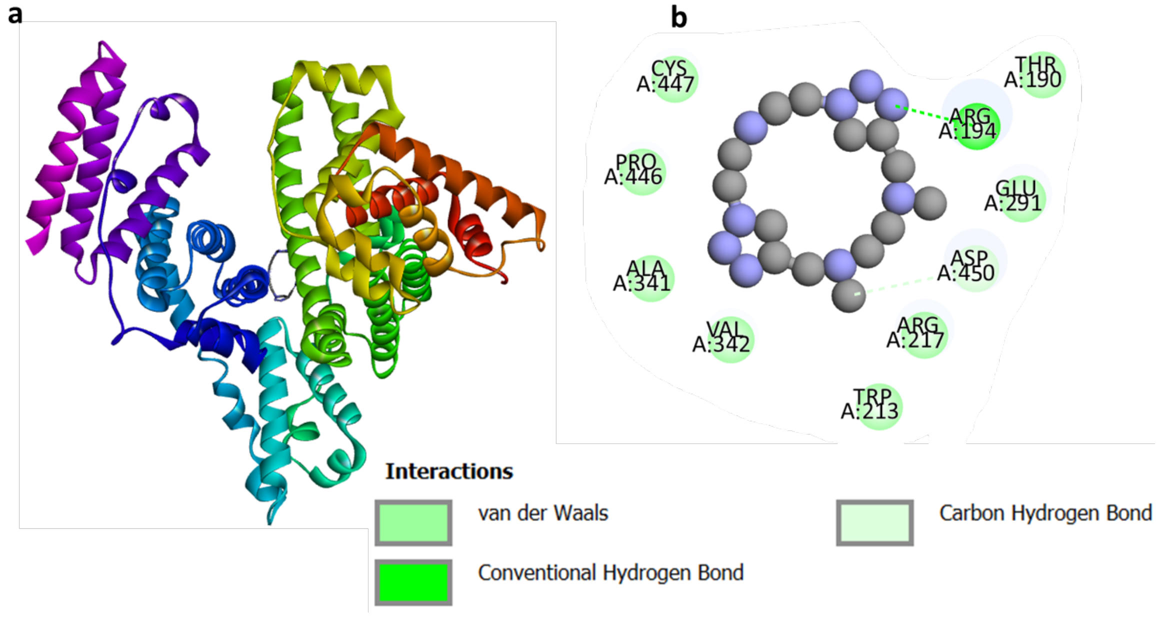

Figure S77. a. The best docked conformation of the **MC5** with the BSA. **b.** 2D diagram of aminoacid interaction of BSA complexed with **MC5**.

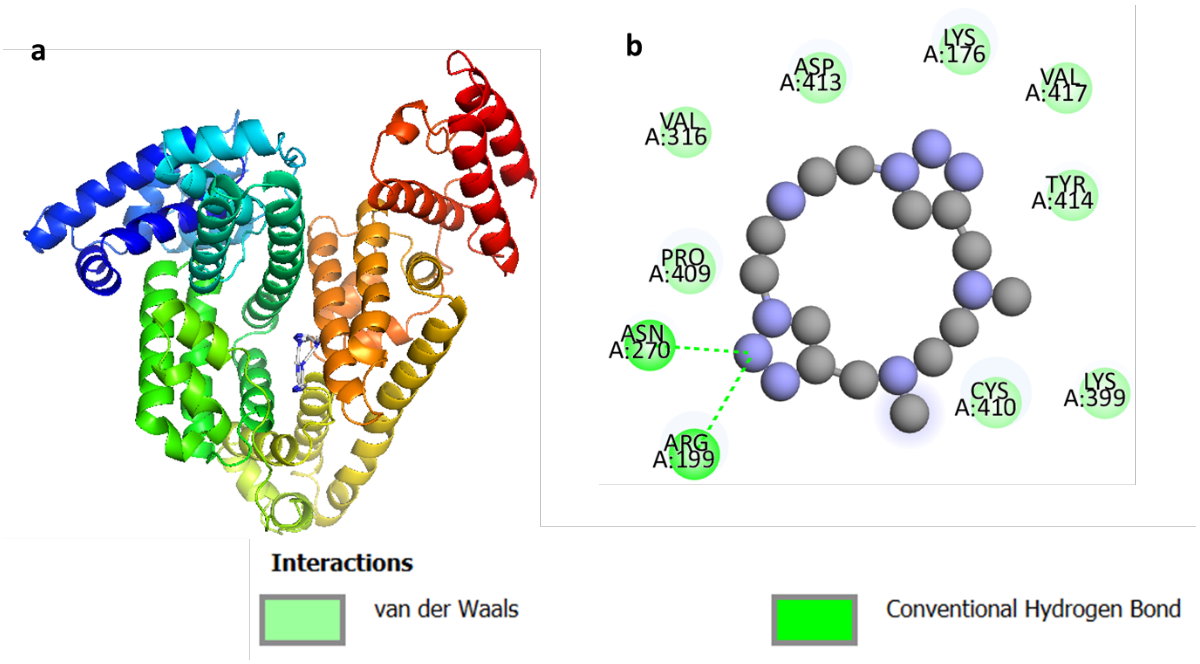

Figure S78. a. The best docked conformation of the **MC5** with the HSA. **b.** 2D diagram of aminoacid interaction of HSA complexed with **MC5**.

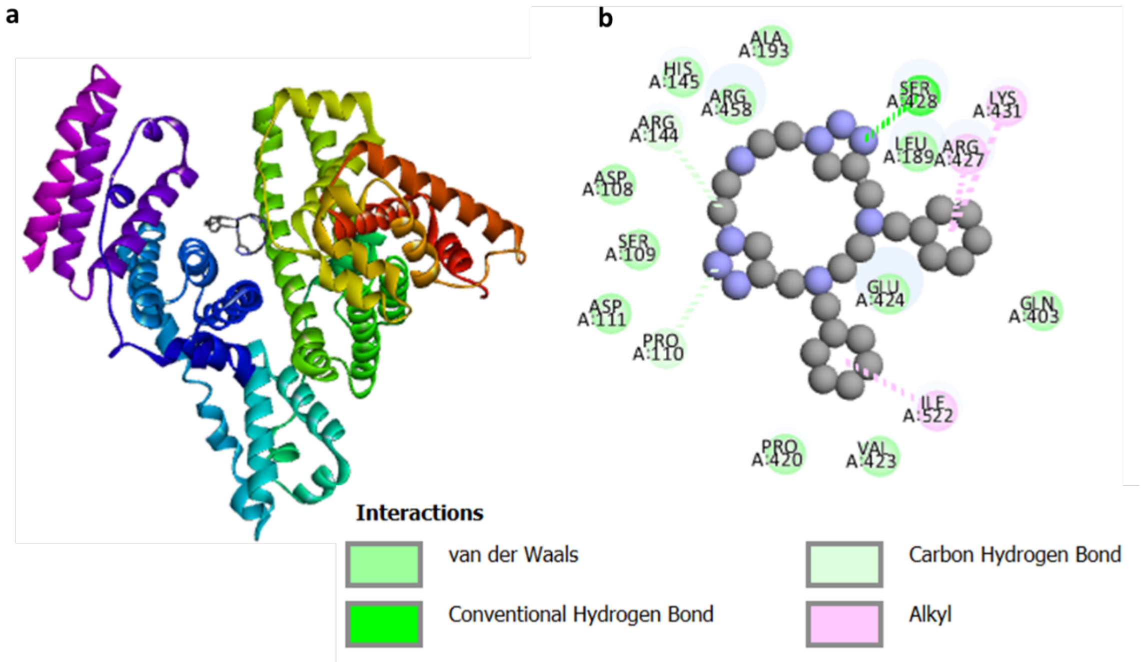

Figure S79. a. The best docked conformation of the **MC6** with the BSA. **b.** 2D diagram of aminoacid interaction of BSA complexed with **MC6**.

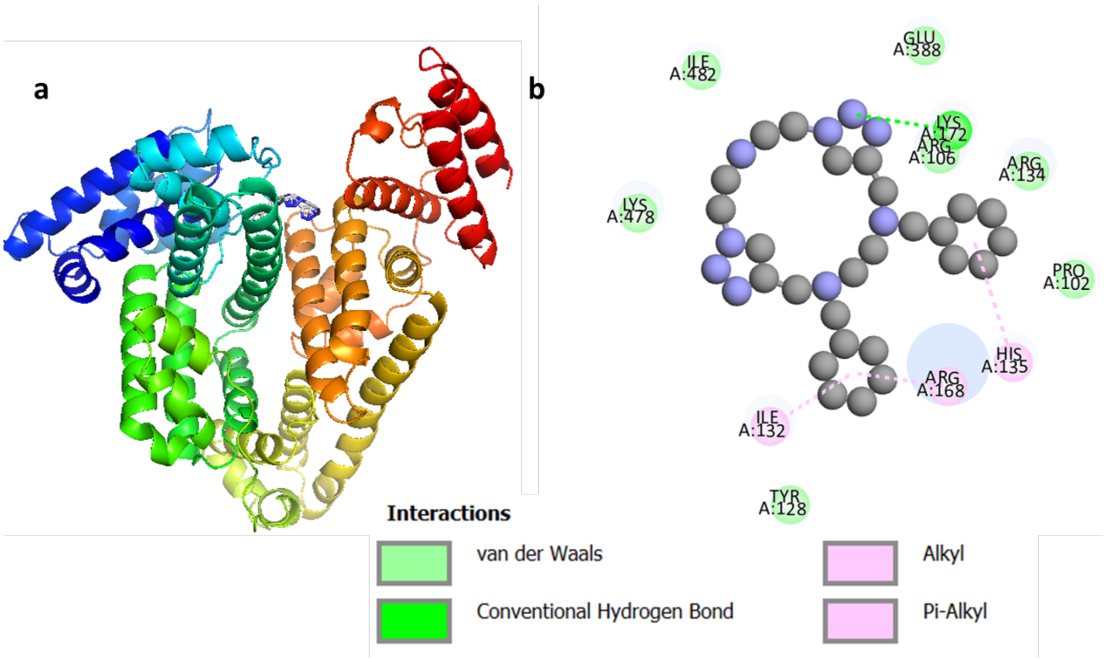

Figure S80. a. The best docked conformation of the **MC6** with the HSA. **b.** 2D diagram of aminoacid interaction of HSA complexed with **MC6**.

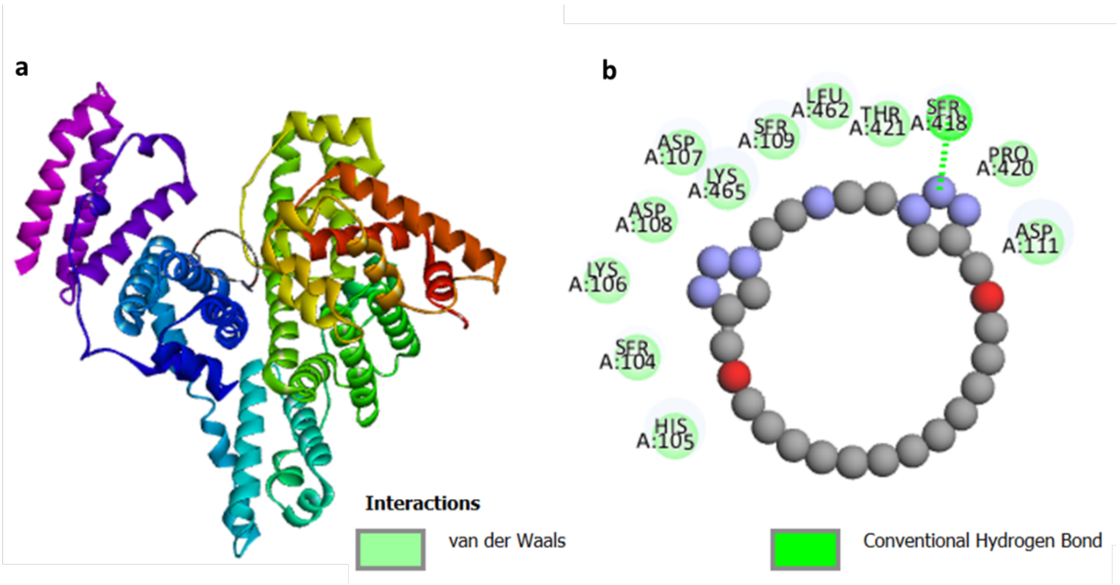

Figure S81. a. The best docked conformation of the **MC7** with the BSA. **b.** 2D diagram of aminoacid interaction of BSA complexed with **MC7**.

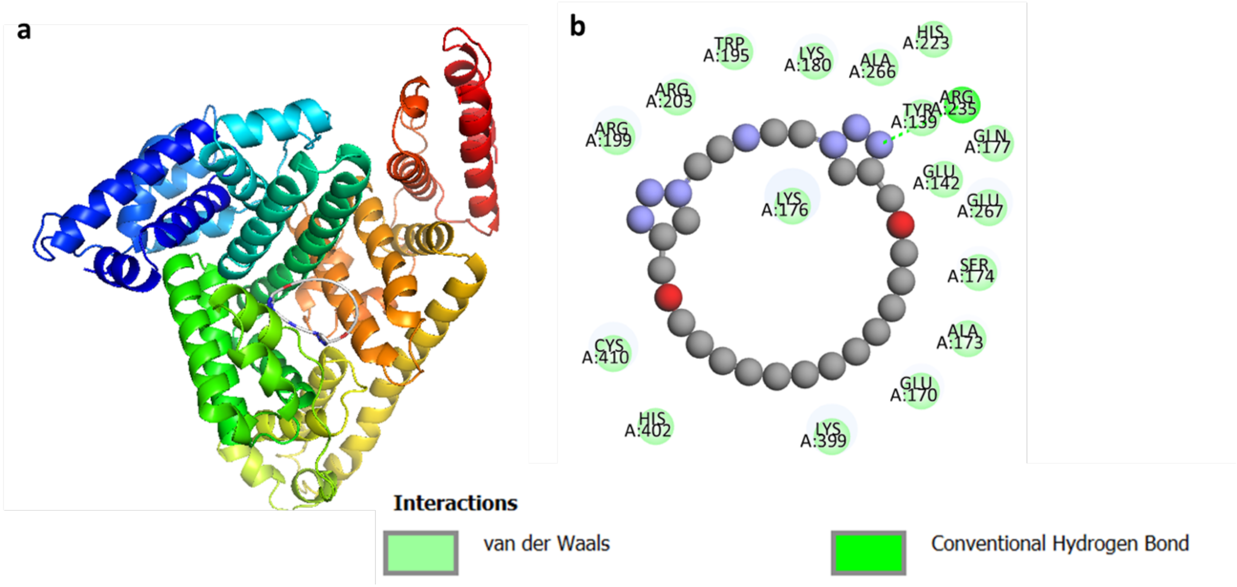

Figure S82. a. The best docked conformation of the **MC7** with the HSA. **b.** 2D diagram of aminoacid interaction of HSA complexed with **MC7**.
